# GS-441524-Diphosphate-Ribose Derivatives as Nanomolar Binders and Fluorescence Polarization Tracers for SARS-CoV-2 and Other Viral Macrodomains

**DOI:** 10.1021/acschembio.4c00027

**Published:** 2024-04-22

**Authors:** Kewen Peng, Shamar D. Wallace, Saket R. Bagde, Jialin Shang, Ananya Anmangandla, Sadhan Jana, J. Christopher Fromme, Hening Lin

**Affiliations:** † Department of Chemistry and Chemical Biology, Cornell University, Ithaca, New York 14853, United States; ‡ Department of Molecular Biology and Genetics, Weill Institute for Cell and Molecular Biology, Cornell University, Ithaca, New York 14853, United States; § Howard Hughes Medical Institute, Department of Chemistry and Chemical Biology, Department of Molecular Biology and Genetics, Cornell University, Ithaca, New York 14853, United States

## Abstract

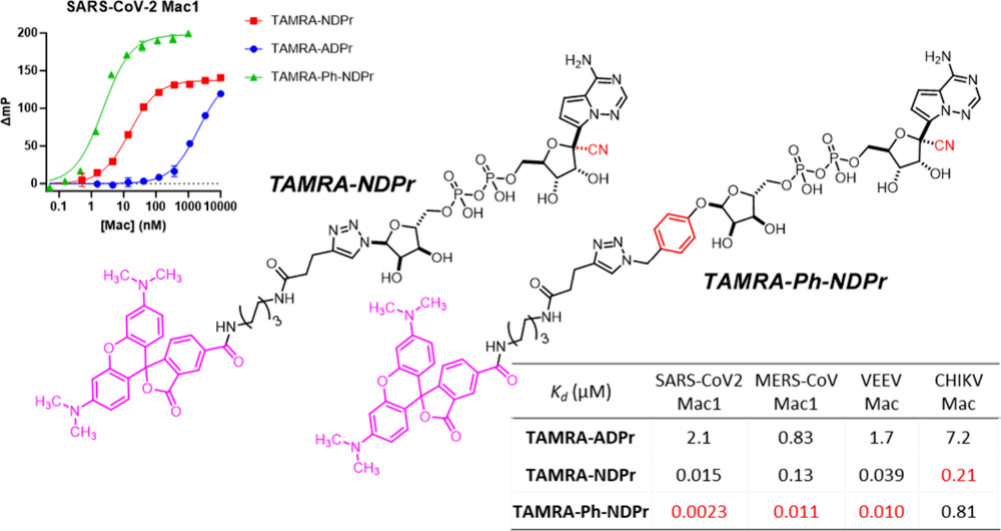

Viral macrodomains that can bind to or hydrolyze protein adenosine diphosphate ribosylation (ADP-ribosylation) have emerged as promising targets for antiviral drug development. Many inhibitor development efforts have been directed against the severe acute respiratory syndrome coronavirus 2 macrodomain 1 (SARS-CoV-2 Mac1). However, potent inhibitors for viral macrodomains are still lacking, with the best inhibitors still in the micromolar range. Based on **GS-441524**, a remdesivir precursor, and our previous studies, we have designed and synthesized potent binders of SARS-CoV-2 Mac1 and other viral macrodomains including those of Middle East respiratory syndrome coronavirus (MERS-CoV), Venezuelan equine encephalitis virus (VEEV), and Chikungunya virus (CHIKV). We show that the 1′-CN group of **GS-441524** promotes binding to all four viral macrodomains tested while capping the 1″-OH of **GS-441524**-diphosphate-ribose with a simple phenyl ring further contributes to binding. Incorporating these two structural features, the best binders show 20- to 6000-fold increases in binding affinity over ADP-ribose for SARS-CoV-2, MERS-CoV, VEEV, and CHIKV macrodomains. Moreover, building on these potent binders, we have developed two highly sensitive fluorescence polarization tracers that only require nanomolar proteins and can effectively resolve the binding affinities of nanomolar inhibitors. Our findings and probes described here will facilitate future development of more potent viral macrodomain inhibitors.

## Introduction

**Figure 1:**
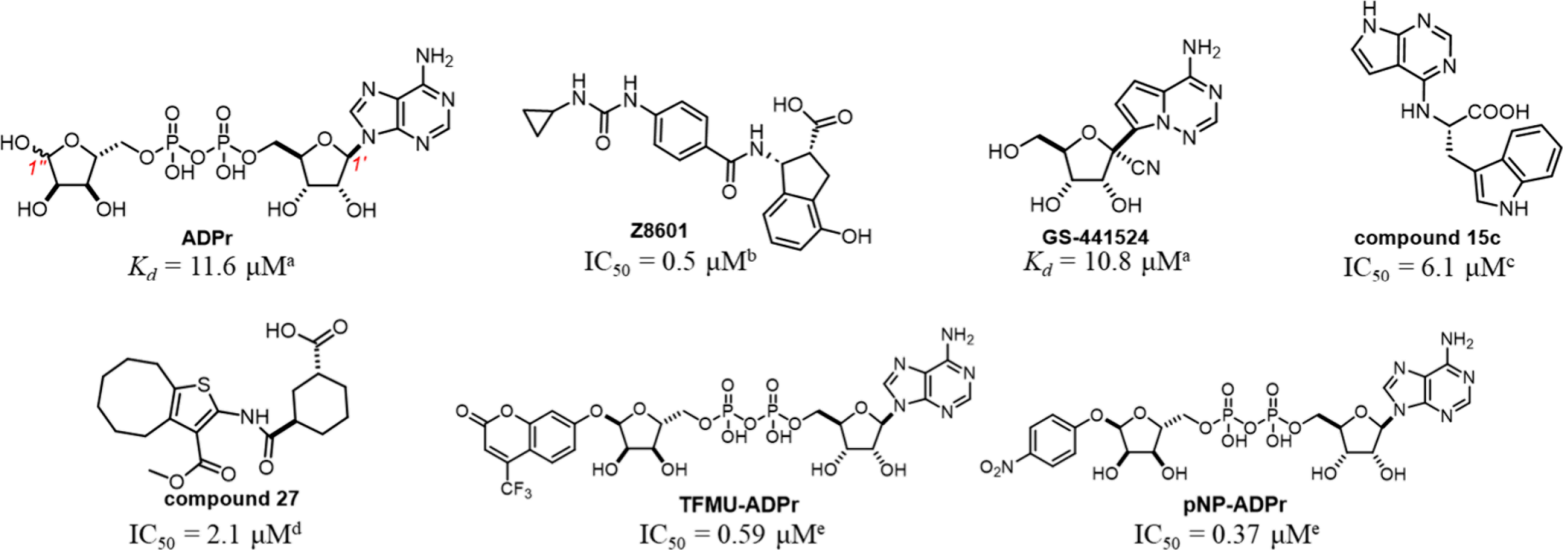
Structures and binding affinities of reported SARS-CoV-2 Mac1 binders. ^a^Determined using ITC. ^b^Determined using an HTRF-based displacement assay where ADPr’s IC_50_ was ∼1 μM. ^c^Determined using an AlphaScreen assay. ^d^Determined by a FRET-based assay where ADPr’s IC_50_ was 1.6 μM. ^e^Determined using an FP assay with **TAMRA-ADPr** as tracer where ADPr’s IC_50_ was ∼15 μM.

Macrodomains are a class of conserved protein domains present in various cells and some viruses with diverse biological functions. They have been characterized as “readers” or “erasers” of protein adenosine diphosphate ribosylation (ADP-ribosylation). They can bind to the adenosine diphosphate ribose (ADPr, Figure 1) attached to proteins and in some cases can remove these post-translational modifications by hydrolyzing the C1″-ester bond between ADPr and the modified Asp or Glu residues.^1−3^ Viral macrodomains, present in all coronaviruses and several other viruses, are interesting targets for antiviral therapies because they can counter host cell immune response by binding to or removing ADP-ribosylation that is crucial for the antiviral signaling pathway in host cells.^4−6^ A single inactivating mutation of macrodomain 1 (Mac1) of severe acute respiratory syndrome coronavirus 2 (SARS-CoV-2) attenuated viral replication in a mouse model,^7,8^ validating macrodomains as promising antiviral targets.

**Figure 2:**
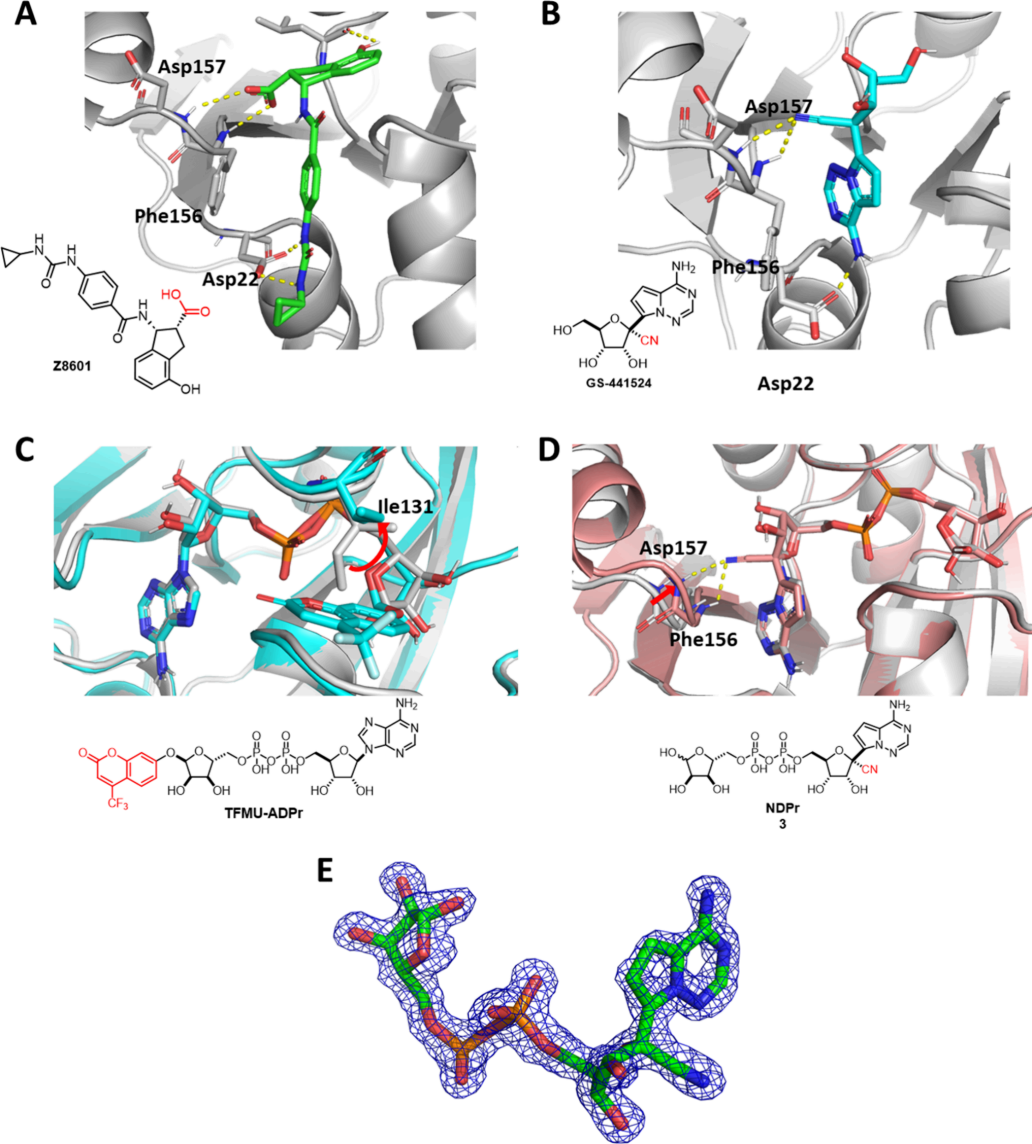
Structures of SARS-CoV-2 Mac1 with different small-molecule inhibitors. (A) Co-crystal structure of SARS-CoV-2 Mac1 with **Z8601** (PDB ID: 5SPD). The urea group of **Z8601** forms hydrogen-bonding interactions with Asp22 while its carboxylic acid moiety occupies the “oxyanion” site formed by the backbone NHs of Phe156 and AsP157. (B) Co-crystal structure of SARS-CoV-2 Mac1 with **GS-441524** (PDB ID: 7BF6). The 1′-CN group of **GS-441524** interacts with the backbone NHs of Phe156 and AsP157. (C) The co-crystal structure of SARS-CoV-2 Mac1 with **TFMU-ADPr** (cyan, PDB ID: 8GIA) is superimposed with that of SARS-CoV-2 Mac1 in complex with ADPr (gray, PDB ID: 6YWL) showing good overall alignment. The Ile131 side chain shows a significant conformational change induced by the TFMU moiety of **TFMU-ADPr**. (D) The co-crystal structure of SARS-CoV-2 Mac1 with **NDPr** (salmon, PDB ID: 9AZX) is superimposed with that of SARS-CoV-2 Mac1 in complex with ADPr (gray, PDB ID: 6YWL). The backbone NHs of Phe156 and Asp157 are shown as sticks. The key interacting structural motif of each inhibitor discussed in the main text is highlighted in red. (E) Electron density of the NDPr ligand bound to SARS-CoV-2 Mac1 in the crystal structure. To generate unbiased density, a simulated-annealing refinement was performed using a model omitting the ligand. The resulting 2Fo-Fc density map is shown, contoured at 1.25σ.

Since the COVID-19 pandemic, there has been an upsurge in the discovery of chemical entities targeting SARS-CoV-2 Mac1 as novel antiviral drugs. Through a combined fragment-screening and linking strategy, Gahbauer et al.^9,10^ identified **Z8601** (Figure 1) as a SARS-CoV-2 Mac1 inhibitor that is more potent than ADPr. Structural analysis (Figure 2A) showed that the urea motif of **Z8601** mimics the adenine amino group in ADPr and interacts with Asp22 of SARS-CoV-2 Mac1 while the carboxylic acid occupies an “oxyanion subsite” enclosed by the backbone NHs of Phe156 and Asp157. Schroder et al.^11^ discovered that **GS-441524**, a metabolite of the anti-SARS-CoV-2 drug remdesivir,^12−14^ binds SARS-CoV-2 Mac1 with an affinity comparable to that of ADPr. Interestingly, they found that the cyano group of **GS-441524** similarly occupies the oxyanion subsite by interacting with the backbone NHs of Phe156 and Asp157 (Figure 2B). Sherrill and colleagues^15^ designed several pyrrolopyrimidine-based amino acid derivatives as SARS-CoV-2 Mac1 inhibitors, among which **compound15c** (Figure 1) is the most potent. Molecular docking of **compound15c** suggested that the pyrrolopyrimidine core mimics the adenine ring of ADPr while the carboxylic acid is placed at the oxyanion subsite. In a very recent manuscript, **compound27** (Figure 1)^16^ was identified as a moderate SARS-CoV-2 Mac1 inhibitor that is effective in cells. The co-crystal structure of this compound with SARS-CoV-2 Mac1 indicates that its carboxylic acid can also interact with the oxyanion subsite. Thus, it appears that the oxyanion subsite of SARS-CoV-2 Mac1 is a “hot spot” for binding that is not utilized by ADPr but can be actively incorporated into the inhibitor design.

Previously, to help identify SARS-CoV-2 Mac1 inhibitors, we developed a fluorescence polarization (FP) assay using a tracer molecule **TAMRA-ADPr** that works for several viral and human macrodomains.^17^ After binding to a macrodomain, the FP of **TAMRA-ADPr** is increased, which can be read by using a plate reader. If a compound competes with the tracer in macrodomain binding, the FP signal would decrease. Intriguingly, using this assay, we discovered that **TFMU-ADPr** and **pNP-ADPr** (Figure 1), originally designed as fluorescent enzymatic substrates for poly(ADP-ribosyl)glycohydrolase (PARG),^18^ are submicromolar binders of SARS-CoV-2 Mac1, which are >20-fold more potent than ADPr. The co-crystal structure of **TFMU-ADPr** and SARS-CoV-2 Mac1 revealed that the TFMU ring at the exit of the binding pocket induces significant conformational change of the side chain of Ile131 (Figure 2C) and likely the hydrophobic interaction between the TFMU ring and the Ile131 side chain contributes to the increased binding affinity.^17^

Here, we designed and synthesized several novel ADPr-based viral macrodomain inhibitors and FP tracers that incorporate the two important structural motifs proven to enhance SARS-CoV-2 Mac1 binding, an H-bonding acceptor to occupy the oxyanion site and an aromatic ring at the 1″-OH position to interact with Ile131 of SARS-CoV-2 Mac1. We obtained nanomolar (including single-digit nanomolar) binders of multiple viral macrodomains, including those of SARS-CoV-2, Middle East respiratory syndrome coronavirus (MERS-CoV), Venezuelan equine encephalitis virus (VEEV), and Chikungunya virus (CHIKV). We show that the affinity boost resulting from occupying the oxyanion site applies to all four viral macrodomains. Additionally, we show that a simple phenyl ring attached to the 1″-OH position can promote binding to SARS-CoV-2 Mac1, MERS-CoV Mac1, and VEEV Mac, but not CHIKV Mac. Importantly, the binding affinity contributions from the above two structural modifications are additive and compounds possessing both motifs proved to be the most potent. These findings also enable us to create FP tracers that are much more potent than **TAMRA-ADPr** and can effectively resolve the binding affinities of these nanomolar inhibitors which **TAMRA-ADPr** cannot discriminate. Our work provides insights for future design of potent viral macrodomain inhibitors, and the improved FP tracers will be effective tools to evaluate the more potent inhibitors.

## Results and Discussion

**Figure 3:**
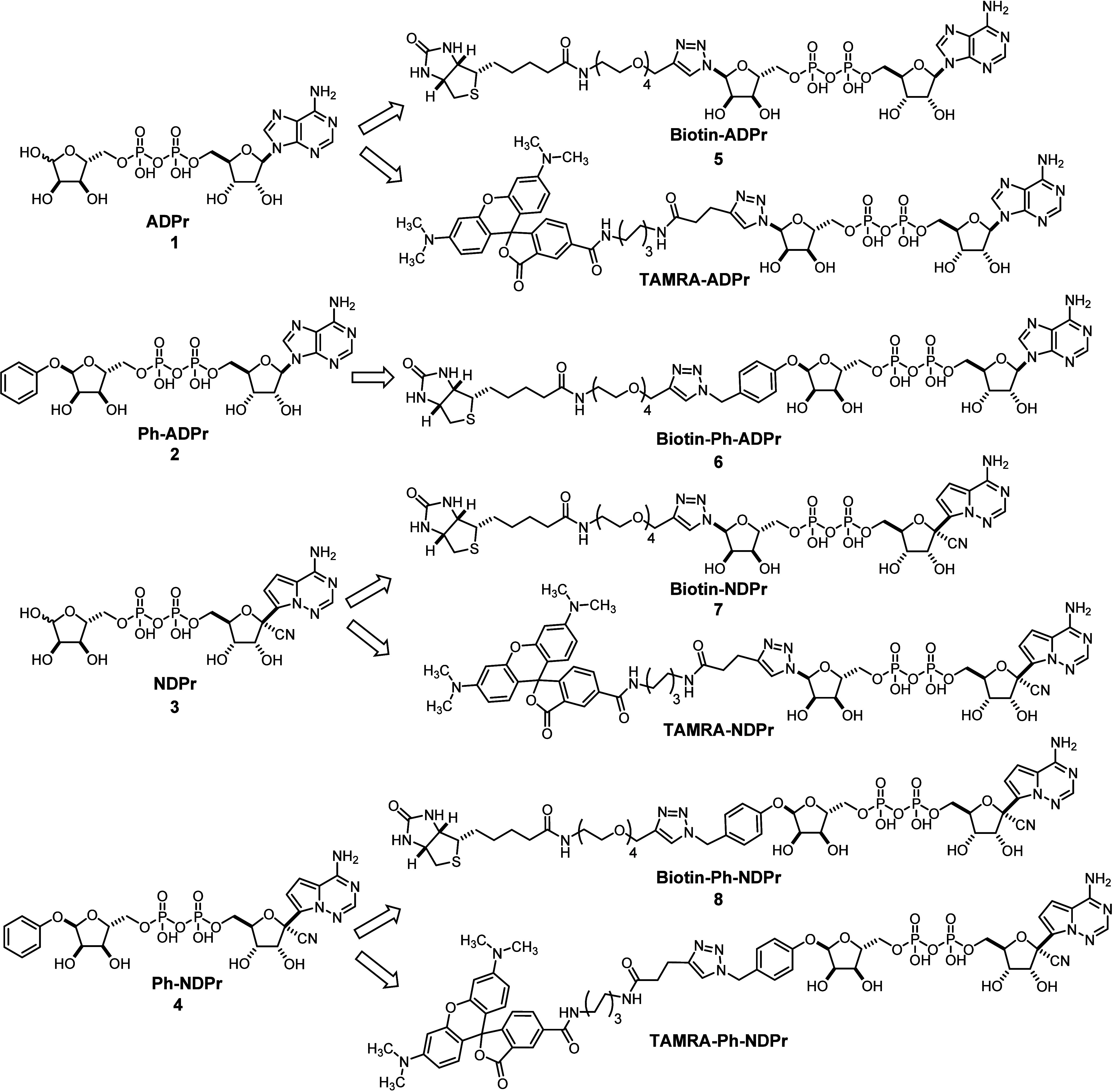
Chemical structures of ADPr (**1**), designed inhibitors (**2**–**4**), their biotinylated versions for biolayer interferometry (**5**–**8**), and TAMRA-labeled tracers for FP assays.

**TFMU-ADPr** and **pNP-ADPr** are more potent binders for SARS-CoV-2 Mac1 than ADPr due to the hydrophobic interaction introduced by the aromatic rings in **TFMU-ADPr** and **pNP-ADPr**. Therefore, we decided to install a simple phenyl ring to create **Ph-ADPr** (**2**, Figure 3). On the other hand, we were interested in **GS-441524** (hereafter referred to as Nuc or N in compound naming for simplicity), which mimics adenosine but has a 1′-CN group that endows this compound with moderate SARS-CoV-2 Mac1 binding capability. We wanted to explore whether converting **GS-441524** into the corresponding **NDPr** (**3**, Figure 3) would further increase its binding affinity for SARS-CoV-2 Mac1. Finally, we designed a third compound, **Ph-NDPr** (**4**, Figure 3), which has an additional 1″-OPh group compared to **NDPr**. In addition, four biotin-labeled compounds: **biotin-ADPr**, **biotin-Ph-ADPr**, **biotin-NDPr**, and **biotin-Ph-NDPr** (**5**–**8**, Figure 3) were synthesized to enable the determination of binding kinetics of the designed ligands toward different viral macrodomains using biolayer interferometry.

**Scheme 1:**
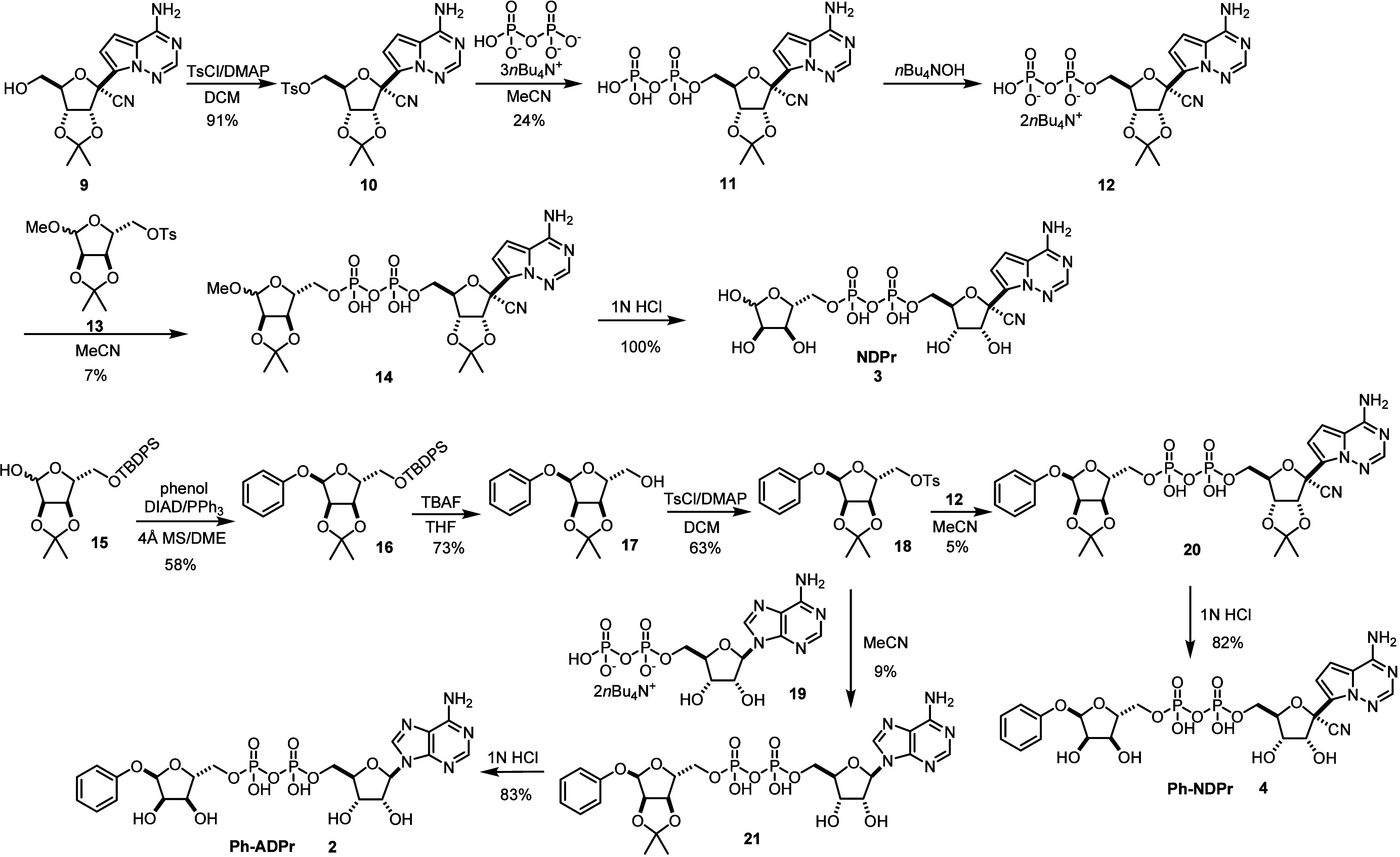
Synthetic Routes for **NDP** (**3**), **Ph-ADPr** (**2**), and **Ph-NDPr** (**4**)

Synthetic routes to **NDPr**, **Ph-ADPr**, and **Ph-NDPr** are depicted in Scheme 1. Previously reported 2′,3′-isopropylidene protected nucleoside **9**(19) was reacted with 4-toluenesulfonyl chloride to give the 5′-*O*-tosyl nucleoside **10**, which was then treated with tris(tetra-*n*-butylammonium) hydrogen pyrophosphate in acetonitrile using a reported procedure,^20^ giving 5′-diphosphate nucleoside **11** in moderate yield. **11** was converted to its di(tetra-*n*-butylammonium) salt **12** and reacted with protected 5′-OTs-*D*-ribose **13** in acetonitrile to furnish protected **NDPr** (**14**). Finally, **NDPr** was obtained by the deprotection of **14** in dilute HCl at 4 °C overnight. **Ph-ADPr** and **Ph-NDPr** were prepared using similar methods, but the key shared intermediate **18** was prepared in three steps from 5′-OTBDPS protected ribose **15**. The Mistunobu reaction of **15** with phenol afforded the α-anomer **16** in high diastereoselectivity. Deprotection and subsequent tosylation of **16** at 5′-OH yielded key intermediate **18** that was reacted with the di(tetra-*n*-butylammonium) salts of NDP (**12**) or ADP (**19**) to give protected **Ph-ADPr** (**20**) or protected **Ph-NDPr** (**21**), respectively, in low to moderate yields. Deprotection of **20** and **21** with aqueous HCl furnished target compounds **Ph-ADPr** (**2**) and **Ph-NDPr** (**4**).

**Scheme 2:**
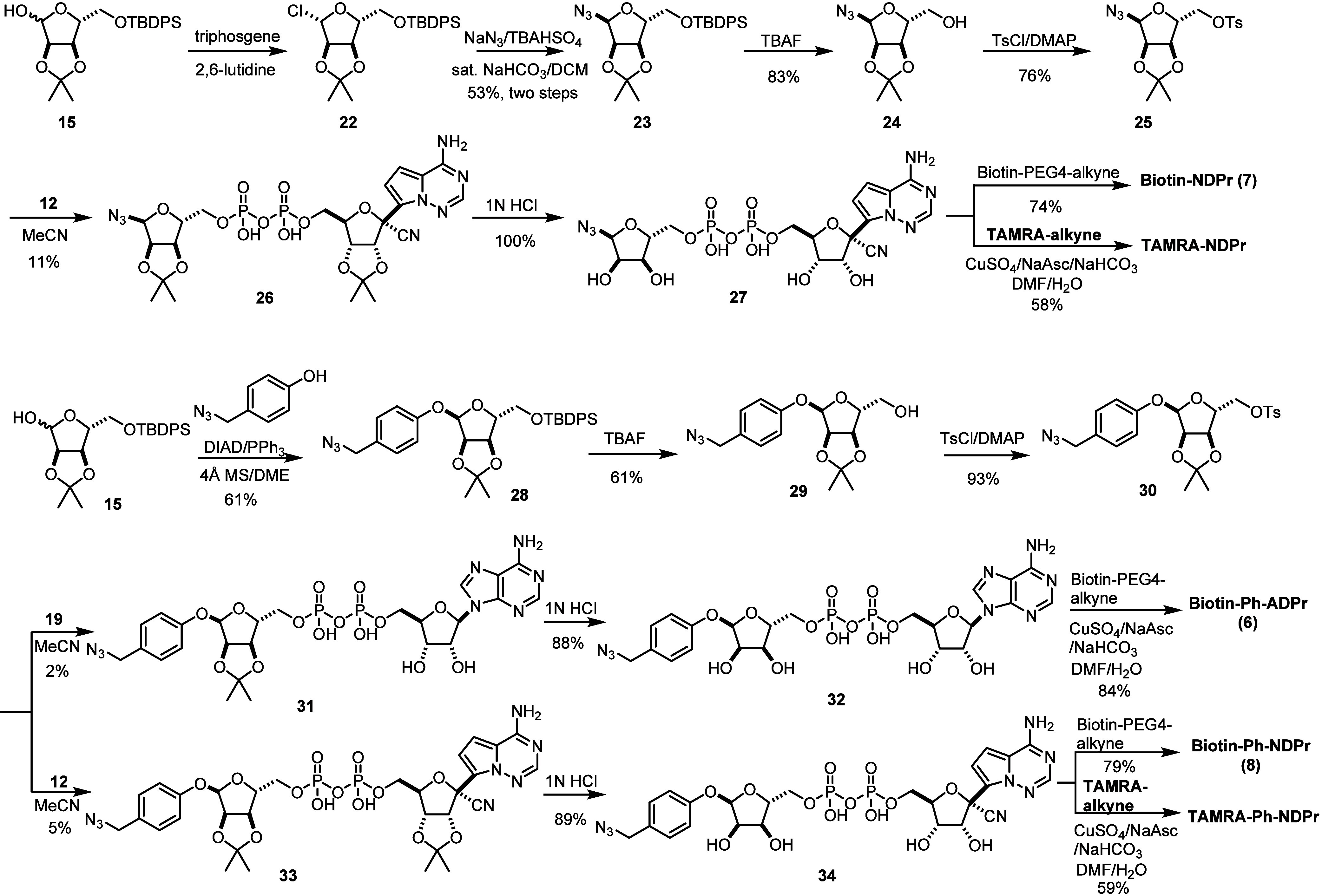
Synthetic Routes for the Biotinylated Ligands **biotin-NDPr** (**7**), **biotin-Ph-ADPr** (**6**), and **biotin-Ph-ADPr** (**8**), and FP Tracers **TAMRA-NDPr** and **TAMRA-Ph-NDPr**

**Biotin-ADPr** (**5**) was prepared using a click reaction between α-ADPr-N_3_^17^ and commercially available biotin-PEG4-alkyne. To synthesize **biotin-NDPr** (**7**), we developed a route for the key intermediate α-NDPr-N_3_ (**27**) (Scheme 2). The route starts with the chlorination of protected ribose **15** at the anomeric position using triphosgene as the chlorine source^21^ and 2,6-lutidine as the base, affording glycosyl chloride **22** as a mixture of anomers (α:β = 3:7). The use of 2,6-lutidine as the base was crucial, as switching it to less sterically hindered pyridine failed to deliver any desired product. **22** was reacted with sodium azide with phase transfer catalysis to afford diastereomerically pure α-glycosyl azide **23** after purification. Subsequent removal of TBDPS protection and tosylation of 5′-OH afforded 5′-tosylate **25**, which was then reacted with the aforementioned di(tetra-*n*-butylammonium) salt of NDP (**12**) to give protected α-1″-N_3_-NDPr (**26**). Deprotection of **26** and subsequent click reaction with biotin-PEG4-alkyne furnished **biotin-NDPr** (**7**). N_3_-Ph-ADPr (**32**) and N_3_-Ph-NDPr (**34**), precursors for the synthesis of **biotin-Ph-ADPr** (**6**) and **biotin-Ph-NDPr** (**8**), were prepared in routes similar with that for **Ph-ADPr** and **Ph-NDPr**, except that phenol was switched to *p*-azidomethyl phenol^22^ in the Mistunobu reaction with **15** at the start of the synthesis (Scheme 2).

Having obtained the designed inhibitors and their biotinylated counterparts, we next tested their ability to bind to the SARS-CoV-2 Mac1. Biolayer interferometry (BLI) has been extensively used in the literature to characterize the binding kinetics of antigen–antibody interactions^23,24^ and small-molecule ligands binding to macromolecules.^25−27^ Recently, we succeeded in characterizing the binding kinetics of isoADPr with the RNF146 WWE domain by loading streptavidin biosensors with biotin-isoADPr, giving stable sensorgrams that yielded *K*_d_ values comparable to the reported value determined through other biophysical methods.^28^ Therefore, we used a similar approach here to study the binding kinetics between SARS-CoV-2 Mac1 and biotinylated ligands.

**Figure 4:**
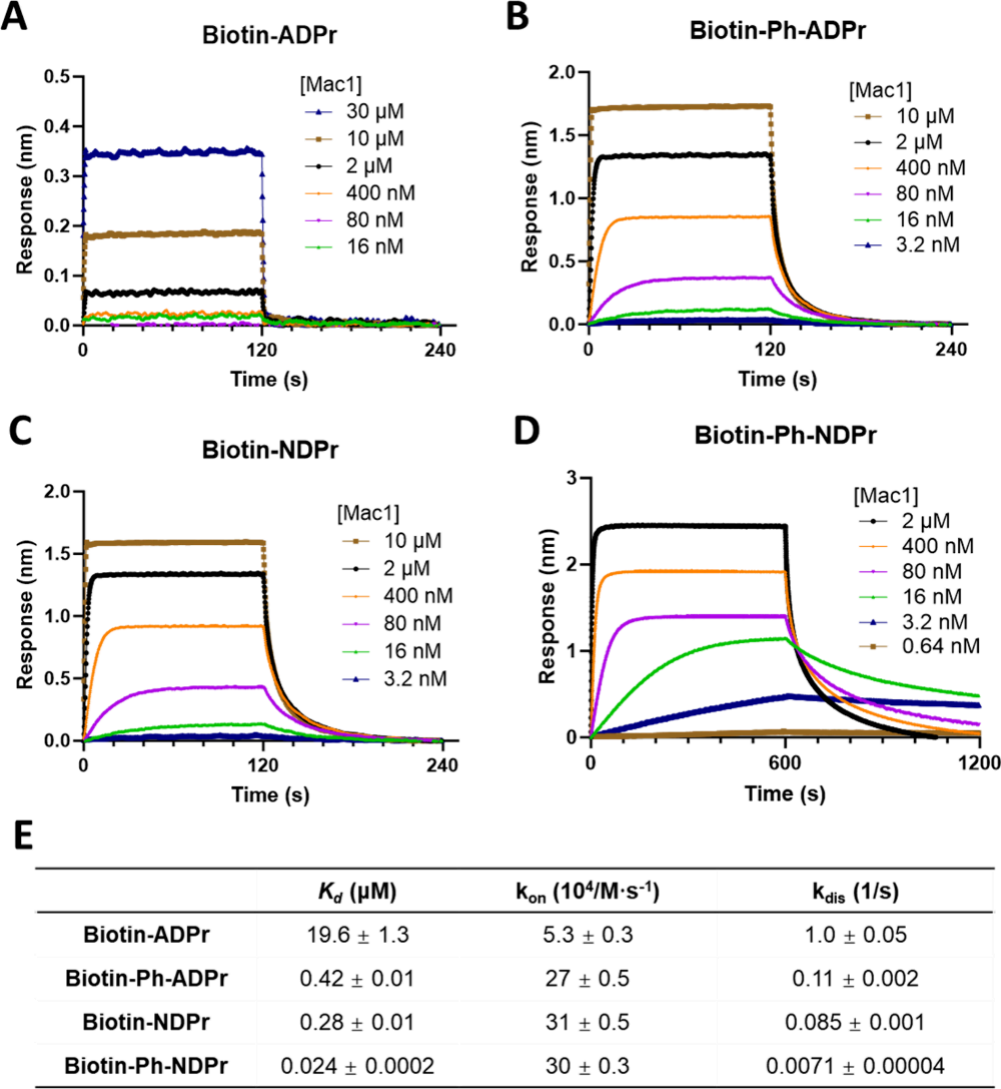
Biolayer interferometry data of binding of SARS-CoV-2 Mac1 to immobilized biotinylated ligands. Sensorgrams of streptavidin biosensors loaded with (A) **biotin-ADPr**, (B) **biotin-Ph-ADPr**, (C) **biotin-NDPr**, and (D) **biotin-Ph-NDPr** that were dipped into SARS-CoV-2 Mac1 solutions at indicated concentrations. (E) Table summarizing the fitted *K*_d_, *k*_on_, and *k*_dis_ values calculated in the Octet BLI analysis software.

Streptavidin biosensors were loaded with different biotinylated ligands and then dipped into wells containing different concentrations of SARS-CoV-2 Mac1 in multiple association–dissociation cycles. The recorded sensorgrams were processed and aligned, and the kinetics data including dissociation constants (*K*_d_), association rates (*k*_on_), and dissociation rates (*k*_dis_) were fitted with the 1:1 binding model. As shown in Figure 4, immobilized **biotin-ADPr** binds SARS-CoV-2 Mac1 with *K*_d_ of 19.6 μM, which is comparable to the reported *K*_d_ of ADPr binding to the same protein (*K*_d_ = 11.6 μM) determined through isothermal titration.^29^ We were pleased to find that **biotin-Ph-ADPr** and **biotin-NDPr** are ∼50- and ∼70-fold, respectively, more potent than **biotin-ADPr** based on the *K*_d_ values (Figure 4), demonstrating the beneficial roles of the 1″-OPh moiety and the 1′-CN group in binding. **Biotin-Ph-NDPr** that incorporates both 1″-OPh and 1′-CN had a *K*_d_ value of only 24 nM, a striking ∼1000-fold decrease compared to that of **biotin-ADPr**. The binding affinity gains are mainly caused by decreases in the dissociation rates rather than faster on-rates, best evidenced by **biotin-Ph-NDPr** whose *k*_dis_ was more than 100-fold smaller than that of **biotin-ADPr** while its *k*_on_ was merely sixfold larger. For drug development, slower dissociation rate or higher residence time (1/*k*_dis_) of the inhibitor, rather than binding affinity, has been better correlated with in vivo activity and should be prioritized.^30,31^

To understand how **NDPr** binds to SARS-CoV-2 Mac1, we solved the X-ray crystal structure of SARS-CoV-2 Mac1 in complex with **NDPr** using diffraction data to 1.4 Å (Figure 2D). As expected, the binding pose of **NDPr** is well-aligned with that of ADPr, except that the 1″-CN group of **NDPr** forms hydrogen-bonding interactions with the backbone NHs of Phe156 and Asp157. Compared with the co-crystal structure of SARS-CoV-2 Mac1 with ADPr, the nearby β7-α6 loop moves significantly to allow interactions with the extra cyano group of **NDPr**, a feature also seen in the binding pose of **GS-441524**.

**Figure 5:**
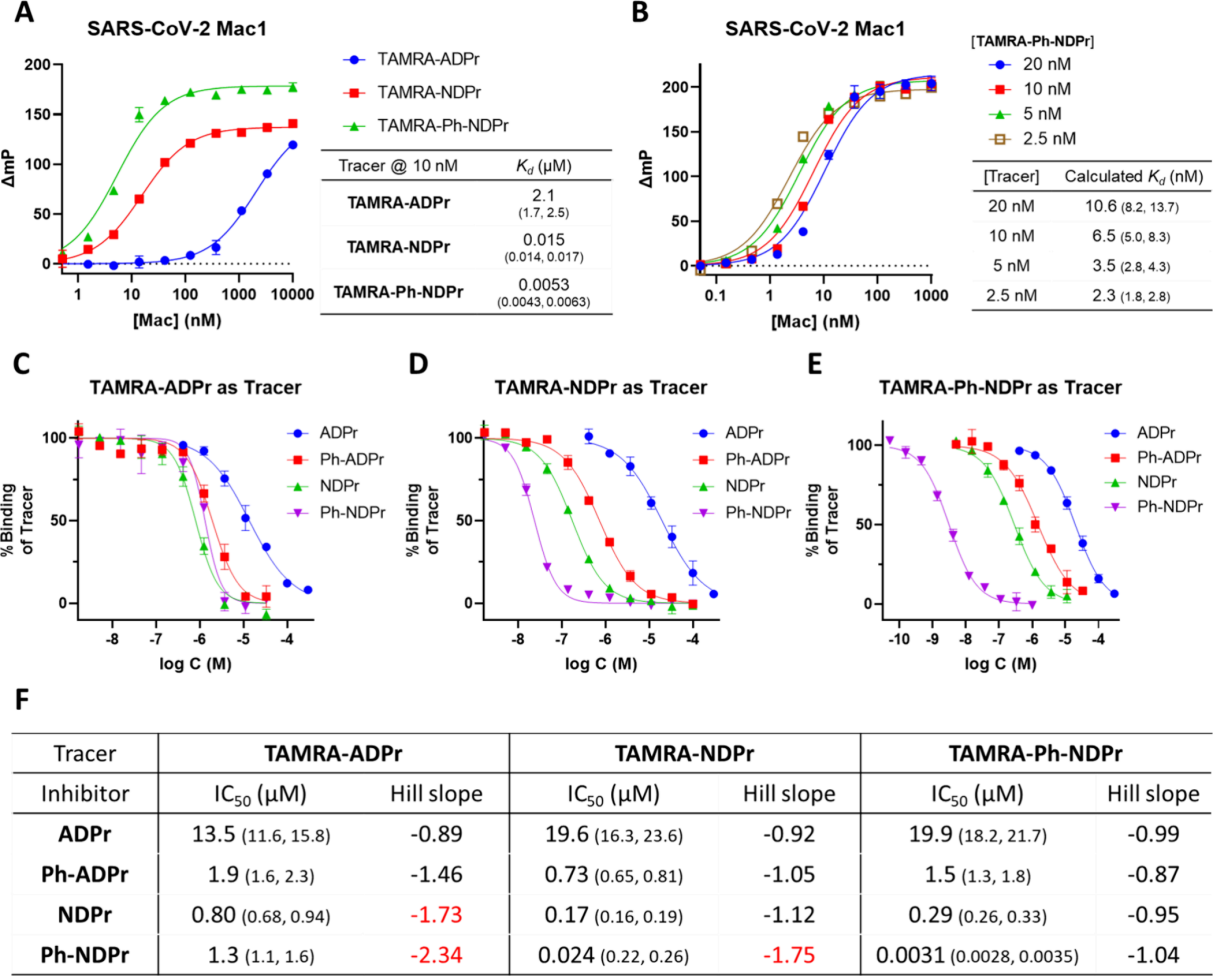
Fluorescence polarization (FP) assay data for different tracers and inhibitors with SARS-CoV-2 Mac1. (A) FP titration of SARS-CoV-2 Mac1 with three different tracers at 10 nM and a table summarizing the fitted *K*_d_ value of each tracer. (B) FP titration of SARS-CoV-2 Mac1 with **TAMRA-Ph-NDPr** and a table of fitted *K*_d_ values at different tracer concentrations. (C) IC_50_ determination of SARS-CoV-2 Mac1 inhibitors using **TAMRA-ADPr** as tracer in FP assays showing poor resolution of potent inhibitors. Protein and tracer were used at 1.5 μM and 20 nM, respectively. (D) IC_50_ determination of SARS-CoV-2 Mac1 inhibitors using **TAMRA-NDPr** as tracer in FP assays with improved resolution of potent inhibitors. Protein and tracer were used at 30 and 20 nM, respectively. (E) IC_50_ determination of SARS-CoV-2 Mac1 inhibitors using **TAMRA-Ph-NDPr** as tracer in FP assays. Both protein and tracer were used at a low concentration of 2 nM to avoid quasi-stoichiometric titration conditions. (F) Table summarizing fitted IC_50_ and Hill slope values from (C) to (E). Hill slope values that significantly deviate from unity are highlighted in red. All *K*_d_ and IC_50_ values are presented as best-fit values with a 95% confidence interval in parentheses (*n* = 2 or *n* = 3).

Encouraged by the BLI data shown above, we designed two new FP tracers: **TAMRA-NDPr** and **TAMRA-Ph-NDPr** (Figure 3), which were synthesized via click chemistry using N_3_-NDPr (**26**) or N_3_-Ph-NDPr (**34**) and previously reported **TAMRA-alkyne**(17) (Scheme 2). The tracers were first titrated with the SARS-CoV-2 Mac1 protein to obtain their *K*_d_ values (Figure 5A). **TAMRA-NDPr** and **TAMRA-Ph-NDPr** exhibited *K*_d_ values of 15 and 5.3 nM, respectively. Both were over 100-fold more potent than **TAMRA-ADPr** under the same assay conditions. Since **TAMRA-Ph-NDPr** is such a tight binder of SARS-CoV-2 Mac1, use of this tracer at the usual concentrations of 10 to 20 nM in FP assays would result in quasi-stoichiometric titration conditions. As shown in Figure 5B, the calculated *K*_d_ value of **TAMRA-Ph-NDPr** decreased as its concentrations were decreasing, and with tracer concentrations from 20 to 5 nM, the calculated *K*_d_ value was always about half of the tracer concentration used, indicating the tracer binds the protein in a quasi-stoichiometric manner at these high tracer concentrations.

In a typical FP assay, the tracer compound is usually used at a low nanomolar concentration (10 to 100 nM) that does not significantly exceed the *K*_d_ value while ensuring it is high enough to give enough fluorescence signal. For the protein, a general rule of thumb is that the concentration should be around the *K*_d_ value and the assay window should exceed 70 mP. It is important to note that in competitive FP-based binding assays, the range of resolvable inhibitor potency is determined by the affinity of the tracer and more potent tracers are required to distinguish more potent inhibitors.^32,33^ Indeed, although we previously established **TAMRA-ADPr** as a robust FP tracer for SARS-CoV-2 Mac1 that reliably resolved the IC_50_ values of several micromolar inhibitors, it failed to discriminate the binding affinities of the submicromolar inhibitors developed here (Figure 5C,F). For instance, the most potent **Ph-NDPr** was incorrectly ranked to be less potent than **NDPr** (Figure 5F) by using **TAMRA-ADPr** as the tracer. We also observed that the Hill slopes of the IC_50_ curves for the stronger binders are significantly different from the theoretical value of −1 (Figure 5F). Steep dose–response curves have been associated with the enzyme concentration being much higher than the inhibitor *K*_d_ in enzymatic assays, leading to stoichiometric inhibition of the enzyme.^34^ The same principles can be applied here to explain the high Hill slopes of potent inhibitors when weak tracers are used. For instance, **TAMRA-ADPr** requires 1.5 μM of SARS-CoV-2 Mac1 protein to achieve a satisfactory assay window (ΔmP), and thus, theoretically at least 0.75 μM of inhibitor is required to bind half of the Mac1 used according to the 1:1 binding model. Therefore, the lower limit of IC_50_ of the assay is determined by the protein concentration, which is in turn determined by the tracer’s *K*_d_ and the ΔmP window required for reliable measurement. Inhibitors with *K*_d_ values much smaller than the protein concentration cannot be accurately measured and the IC_50_ curves will have high Hill slopes. It is important to note the difference between the stoichiometric inhibition by the inhibitor described here and the quasi-stoichiometric titration condition mentioned earlier. The former is usually associated with the tracer *K*_d_ being much higher than the inhibitor’s inhibition constant (*K*_i_) while the latter happens when the tracer is used at a concentration much higher than its *K*_d_. Using **TAMRA-NDPr** as the tracer, the concentration of SARS-CoV-2 Mac1 can be lowered to 10–50 nM (0.5 to threefold of *K*_d_) with reasonable assay windows. Gratifyingly, the binding affinities of ADPr, **Ph-ADPr**, **NDPr**, and **Ph-NDPr** can be unambiguously resolved with **TAMRA-NDPr** at 20 nM and the protein at 30 nM (Figure 5D). The obtained IC_50_ values are well correlated with the *K*_d_ values of their biotinylated versions measured in BLI experiments (Figures 4 and 5F).

The IC_50_ curve of **Ph-NDPr** using **TAMRA-NDPr** as the tracer was still steeper than normal, suggesting that its binding affinity was underestimated even with **TAMRA-NDPr**. A good dose–response curve of **Ph-NDPr** with Hill slope of unity was obtained using **TAMRA-Ph-NDPr** as the tracer, yielding IC_50_ of 3.1 nM, which was 6000-, 500-, and 100-fold better than ADPr, **Ph-ADPr**, and **NDPr**, respectively, measured under the same assay conditions (Figure 5E,F). Therefore, it appears that the binding affinity gains from the 1″-OPh moiety and the 1′-CN group are additive for SARS-CoV-2 Mac1 inhibitors, which should greatly facilitate future inhibitor designs.

Despite that **TAMRA-Ph-NDPr** has the best resolving power for SARS-CoV-2 Mac1 inhibitors, it should be noted that very low concentration (<2 nM) of this tracer should be used in the screens to avoid quasi-stoichiometric titration conditions where more inhibitor is required to displace the tracer from protein binding (explained in detail in ref (33)) and could thus conceal low-affinity hits from HTS campaigns. Additionally, although a wide assay window of ∼100 mP could be achieved with 2 nM **TAMRA-Ph-NDPr** and 2 nM SARS-CoV-2 Mac1 protein, the fluorescence intensity was only about fivefold higher than the background. This prohibits the screening of inhibitors at high concentrations or if the inhibitors have significant intrinsic fluorescence. Because of these considerations, we found the less potent **TAMRA-NDPr** a better choice for routine screens of SARS-CoV-2 Mac1 inhibitors while the more potent **TAMRA-Ph-NDPr** is more suitable for differentiating compounds that are extremely potent.

Next, we measured the binding between the biotinylated ligands and MERS-CoV Mac1, VEEV Mac, and CHIKV Mac using BLI (Figure S1). The results are summarized in Table 1. Compared with **biotin-ADPr**, **biotin-NDPr** binds to the three viral macrodomains 10- to 40-fold stronger, suggesting that the 1′-CN group in **biotin-NDPr** boosts the binding to these macrodomains. The 1″-OPh moiety, on the other hand, promotes only the binding of MERS-CoV Mac1 and VEEV Mac, but not CHIKV Mac. For MERS-CoV Mac1 and VEEV Mac, respectively, **biotin-Ph-ADPr** has a 20- and 30-fold decrease in *K*_d_ compared to **biotin-ADPr** while the *K*_d_ difference is relatively small for CHIKV Mac. **Biotin-Ph-NDPr** is the strongest binder of MERS-CoV Mac1 and VEEV Mac with *K*_d_ of 17.2 and 14.0 nM, respectively, ∼100-fold and 400-fold lower than that of **biotin-ADPr**. For CHIKV Mac, **biotin-NDPr** is the most potent binder with a *K*_d_ of 331 nM, which is only ∼15-fold stronger than **biotin-ADPr**. Similar to what has been observed for SARS-CoV-2 Mac1, the affinity boost for **biotin-Ph-NDPr** is mainly contributed by a much slower off-rate for both MERS-CoV Mac1 (*k*_dis_: 0.031/s) and VEEV Mac (*k*_dis_: 0.014/s), although its dissociation rate for SARS-CoV-2 Mac1 (*k*_dis_: 0.0071/s) is still significantly lower.

**Table 1:** Kinetics Data of Immobilized Biotin Ligands Binding to MERS-CoV, CHIKV, and VEEV Macrodomains^a^

**protein**	**biotin ligand**	*K*_d_**(μM)**	*k*_**on**_**(10**^**4**^**/**M·s^–1^**)**	*k*_**dis**_ (1/s)
MERS-CoV Mac1	**biotin-ADPr**	2.1 ± 0.1	32 ± 1	0.70 ± 0.03
	**biotin-Ph-ADPr**	0.10 ± 0.004	110 ± 3	0.11 ± 0.003
	**biotin-NDPr**	0.21 ± 0.01	130 ± 5	0.27 ± 0.01
	**biotin-Ph-NDPr**	0.017 ± 0.0002	180 ± 1	0.031 ± 0.0002
CHIKV Mac	**biotin-ADPr**	5.3 ± 0.2	12 ± 0.3	0.65 ± 0.02
	**biotin-Ph-ADPr**	6.2 ± 0.2	1.9 ± 0.05	0.12 ± 0.002
	**biotin-NDPr**	0.33 ± 0.01	18 ± 0.4	0.060 ± 0.0009
	**biotin-Ph-NDPr**	0.57 ± 0.02	19 ± 0.6	0.11 ± 0.003
VEEV Mac	**biotin-ADPr**	5.5 ± 0.4	14 ± 0.8	0.79 ± 0.04
	**biotin-Ph-ADPr**	0.16 ± 0.002	52 ± 0.6	0.083 ± 0.0008
	**biotin-NDPr**	0.13 ± 0.002	58 ± 0.6	0.076 ± 0.0008
	**biotin-Ph-NDPr**	0.014 ± 0.0001	82 ± 0.7	0.014 ± 0.00006

a Data are represented as fitted *K*_d_, *k*_on_, and *k*_dis_ values ± error calculated in the Octet BLI Analysis software.

**Figure 6:**
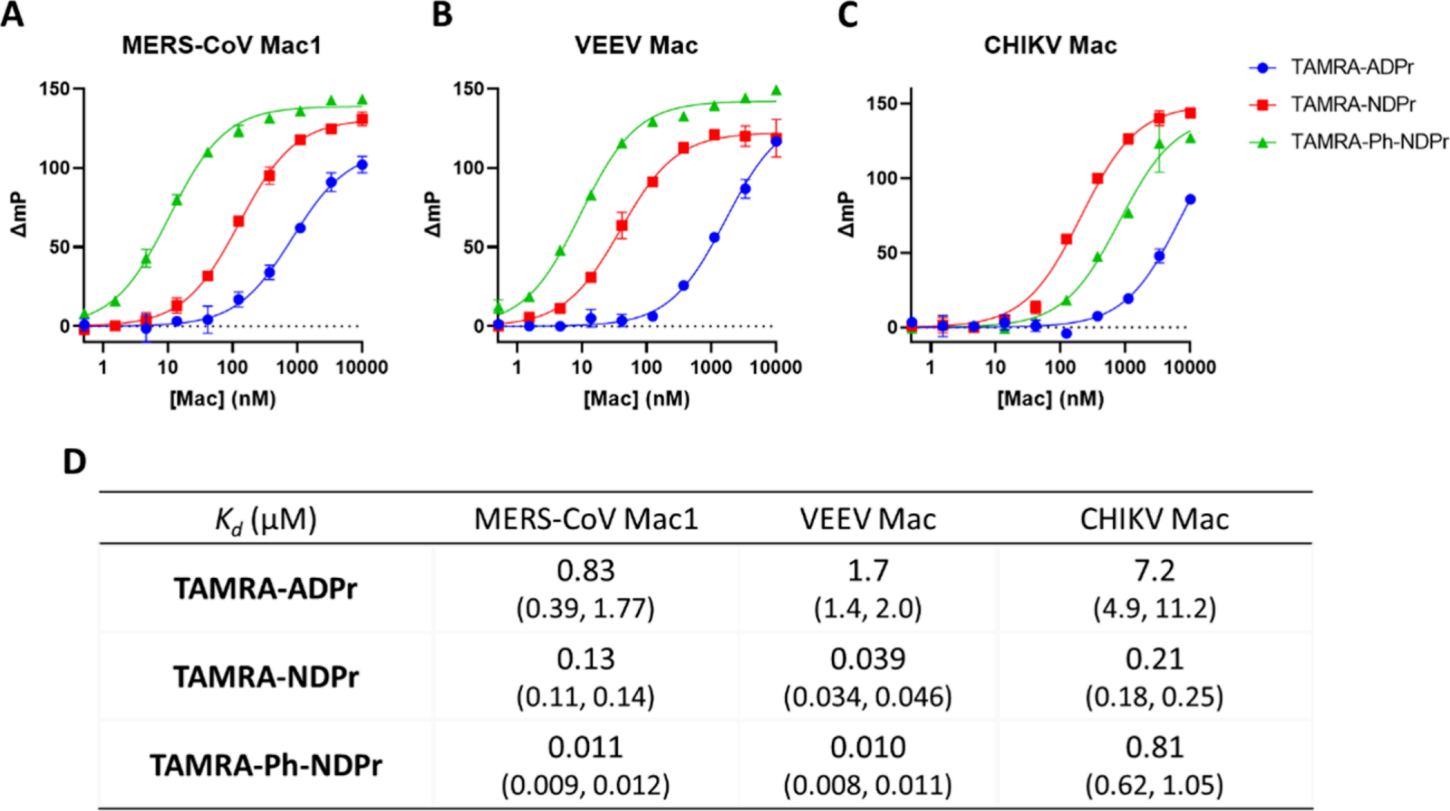
FP titration curves of **TAMRA-ADPr**, **TAMRA-NDPr**, and **TAMRA-Ph-NDPr** at 10 nM with (A) MERS-CoV Mac1, (B) VEEV Mac, and (C) CHIKV Mac. (D) Table summarizing fitted *K*_d_ values of the three tracers toward the different viral macrodomains tested. *K*_d_ values are presented as best-fit values with 95% confidence intervals in parentheses (*n* = 2).

Having measured the binding affinities of the biotinylated ligands for MERS-CoV Mac1, VEEV Mac, and CHIKV Mac, we conducted FP titration experiments of the TAMRA-labeled tracers with the three proteins (Figure 6). The *K*_d_ value of each tracer determined by using FP titration is well correlated with the *K*_d_ value of its biotin-labeled counterpart obtained by using BLI. **TAMRA-Ph-NDPr** is a potent tracer for both MERS-CoV Mac1 and VEEV Mac, with *K*_d_ of 11 and 10 nM, respectively. The larger *K*_d_ values of **TAMRA-Ph-NDPr** for MERS-CoV Mac1 and VEEV Mac than for SARS-CoV-2 Mac1 are actually advantageous for inhibitor screen purposes as this tracer can now be used at higher concentrations (5 to 20 nM) without complications caused by quasi-stoichiometric titration conditions. Consistent with the BLI data, **TAMRA-NDPr** (*K*_d_ 208 nM) is a slightly better tracer for CHIKV Mac than **TAMRA-Ph-NDPr** (*K*_d_ of 810 nM). Therefore, **TAMRA-Ph-NDPr** is a powerful FP tracer for screening MERS-CoV Mac1 and VEEV Mac inhibitors that requires as little as 10–20 nM protein and should be able to resolve the binding affinities of potent inhibitors with *K*_d_ down to 10 nM. Although less potent, **TAMRA-NDPr** is a useful FP tracer for CHIKV Mac with more than 30-fold affinity improvements over **TAMRA-ADPr**, which translates into 30-fold less protein needed, facilitating large-scale inhibitor screening against this target.

**Figure 7:**
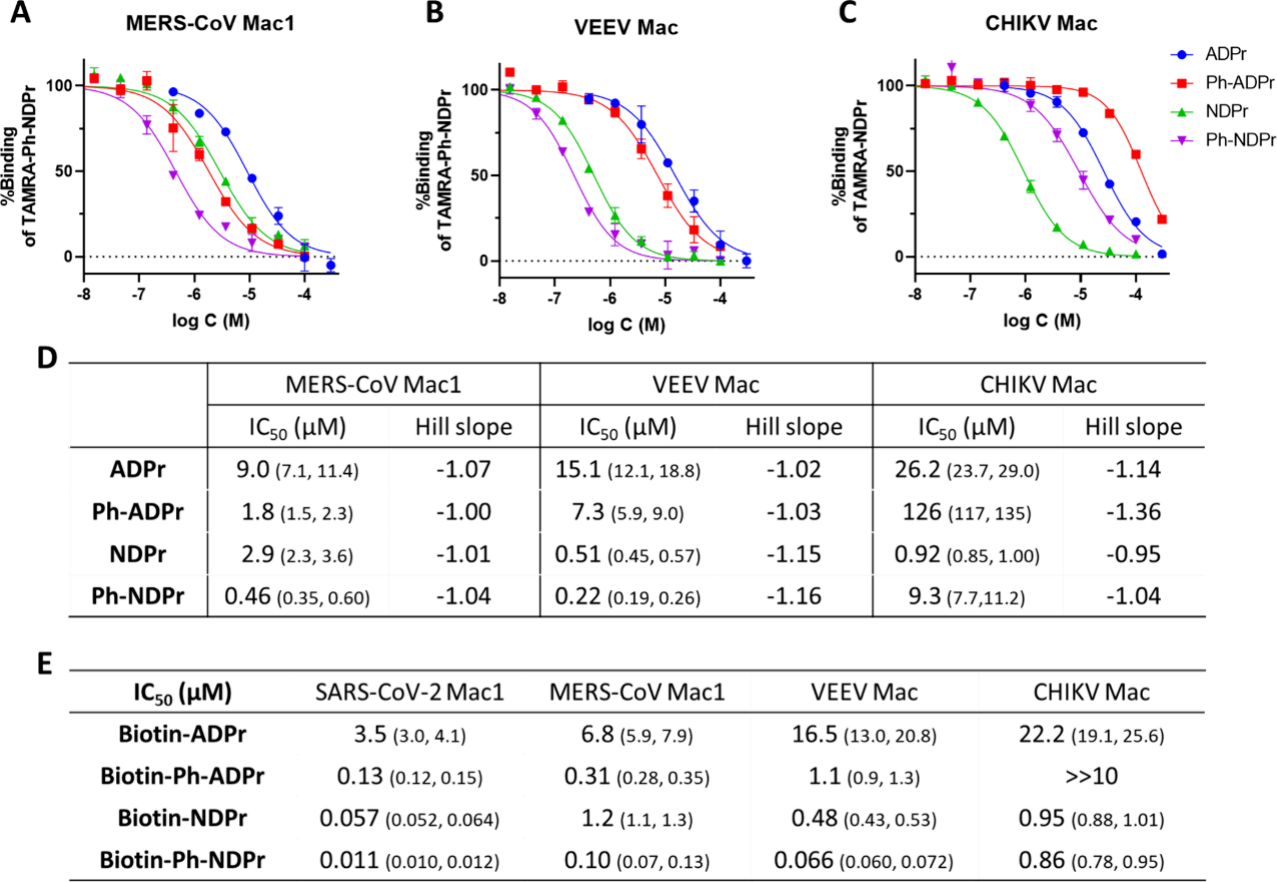
Dose–response curves of **ADPr**, **Ph-ADPr**, **NDPr**, and **Ph-NDPr** in FP-based binding assays of (A) MERS-CoV Mac1 with **TAMRA-Ph-ADPr** as tracer, (B) VEEV Mac with **TAMRA-Ph-ADPr** as tracer, and (C) CHIKV Mac with **TAMRA-NDPr** as tracer. (D) Table summarizing fitted IC_50_ and Hill slope values of the four inhibitors against different viral macrodomains tested. (E) Table summarizing fitted IC_50_ values of the four biotin-labeled inhibitors against different viral macrodomains tested. IC_50_ values are presented as best-fit values with 95% confidence intervals in parentheses (*n* = 2).

As shown in Figure 7, the IC_50_ values of **ADPr**, **Ph-ADPr**, **NDPr**, and **Ph-NDPr** against MERS-CoV Mac1 and VEEV Mac could be nicely resolved using **TAMRA-Ph-NDPr** as the tracer. Similarly, **TAMRA-NDPr** also successfully distinguished **NDPr** as the most potent binder for CHIKV Mac. We were curious to see whether the biotin portion can somehow contribute to viral macrodomain binding and thus tested the biotinylated ligands in the FP assays (Figure 7E). Intriguingly, the biotinylated ligands exhibited lower IC_50_s compared with their nonbiotinylated counterparts. Macrodomains recognize modifications on protein substrates. Thus, secondary interactions outside the ADPr-binding pocket are likely also important for successful substrate recognition. The TAMRA or biotin moieties or the linker region may provide extra interactions with the protein and thus confer stronger binding affinities. We also calculated the inhibitors’ *K*_i_ values (Figure S3) in place of the IC_50_ values using equations that were previously published.^33^*K*_i_ should, in theory, be less affected by experimental conditions (e.g., protein concentration and tracer used). However, the calculated *K*_i_ values for the same inhibitor against the same protein did vary when different tracers were used (Figure S3A). This was likely because some of the IC_50_ values, which was used to calculate the *K*_i_, were not accurate because of the stoichiometric inhibition as discussed earlier. Overall, **TAMRA-NDPr** and **TAMRA-Ph-NDPr** are powerful tracers for all four viral macrodomain tested and allow the screens of nanomolar candidates at much lower costs, representing significant improvements over the original design **TAMRA-ADPr**.

## Conclusions

We synthesized several ADPr mimics as inhibitors or probes of viral macrodomains, including SARS-CoV-2 Mac1, MERS-CoV Mac1, VEEV Mac, and CHIKV Mac. For the first time, we revealed that the 1′-CN group of **GS-441524**, a metabolite of the antiviral drug remdesivir, can significantly promote binding to multiple viral macrodomains other than SARS-CoV-2 Mac1. Interestingly, this cyano group is detrimental to the binding of human MacroD1 and MacroD2 (Figure S2). Therefore, **GS-441524** represents a promising starting point for the development of selective and broad-spectrum antiviral drugs targeting multiple viral macrodomains. We also confirmed that capping the 1″-OH with a simple phenyl ring increases the viral macrodomain binding capabilities. Moreover, the binding affinity gains from 1′-CN and 1″-OPh are additive for most viral macrodomains. Future inhibitor design endeavors could incorporate these two structural motifs while replacing or masking the diphosphate linkage in ADPr to confer cell permeability and metabolic stability. Finally, we developed two novel and potent FP tracers **TAMRA-NDPr** and **TAMRA-Ph-NDPr** that can accurately resolve the binding affinities of nanomolar inhibitors of different viral macrodomains at much lower costs. The newly developed tracers will aid in future screens of viral macrodomain inhibitors with low nanomolar activities.

## Materials and Methods

### Chemical Synthesis

Detailed synthetic procedures can be found in the Supporting Information.

### Expression and Purification of Macrodomains

SARS-CoV-2 Mac1, VEEV Mac and CHIKV Mac, MacroD1, and MacroD2 were purified as previously reported.^17^ Plasmid for MERS-CoV Mac1 was purchased from Twist Biosciences by using NdeI/XhoI cut sites in pET28a vectors (full sequences available in the SI). The plasmids were transformed into BL21(DE3) chemically competent *Escherichia coli*. 4 L of LB broth with 50 μg/mL kanamycin was inoculated with an overnight starter grown at 37 °C. Cultures were grown at 200 rpm and 37 °C for ∼4 h until the OD600 reached 0.8. Then, IPTG was added to 0.5 mM and the cells were incubated at 16 °C overnight to allow protein expression. Cells were harvested by centrifugation at 6000*g*. Cell pellets were frozen at −80 °C or immediately used for purification. Pellets were resuspended in lysis buffer (50 mM Tris (pH 8.0), 500 mM NaCl, 0.5 mg mL^–1^ lysozyme, 1 mM PMSF, and Pierce universal nuclease). Following a 30 min incubation, cells were sonicated on ice for 4 min in total at 60% amplitude. Lysate was clarified at 4 °C and 30,000 × *g* for 35 min. Clarified lysate was loaded onto Ni-NTA resin, washed with 50 mL of wash buffer (50 mM Tris pH 8.0, 500 mM NaCl, 20 mM imidazole), and eluted with elution buffer (50 mM Tris pH 8, 500 mM NaCl, 200 mM imidazole). Crude macrodomains were concentrated using a 10 kDa MWCO Amicon filter and loaded onto a HiLoad 16/600 Superdex 75 gel filtration column equilibrated with storage buffer (25 mM Tris (pH 8.0), 150 mM NaCl, 10% glycerol) on an KTA FPLC system. Fractions containing macrodomains were pooled, concentrated, flash frozen in liquid nitrogen, and stored at −80 °C for future use. For SARS-CoV-2, the sample was supplemented with DTT (2 mM) and tobacco-etch protease and incubated at 4 °C overnight. The reaction mixture was then subjected to subtractive nickel chelate chromatography, and the eluate was injected into a HiLoad 16/600 Superdex 75 gel filtration column equilibrated with protein storage buffer (5 mM HEPES and 150 mM NaCl, pH 7.5). Fractions containing the purified SARS-CoV-2 macrodomain were combined and concentrated. Then samples were aliquoted, flash frozen using liquid nitrogen, and stored at −80 °C.

### Biolayer Interferometry

The binding of the biotin-labeled compounds to different viral macrodomains was monitored and measured on an Octet RH16 biolayer interferometer. Streptavidin biosensor tips were loaded with 1 μM of the biotin-labeled compounds in the kinetics buffer (PBS with 0.02% Tween 20, and 0.1% BSA) for 100 s. After a 100 s baseline step, the loaded sensor tips were moved to sample wells containing viral macrodomain proteins at increasing concentration in the kinetics buffer sequentially in multiple cycles, with each cycle consisting of a 120 s association step in the sample well and a 120 s dissociation step in the buffer well. For **biotin-Ph-NDPr**, the association and dissociation times were elongated to 600 s for binding to SARS-CoV-2 Mac1, MERS-CoV Mac1, and VEEV Mac due to much lower dissociation rates of this compound. The volume of each well was 200 μL. Reference biosensors without loading of the biotin-labeled compounds were used to exclude possibilities of nonspecific binding to the sensor tips, and reference wells without macrodomain protein were used for blank subtraction. Data were processed, and curves fitted with a 1:1 best-fit model in the Octet BLI Analysis software.

### FP Titration of Different Tracers with Viral Macrodomains

Procedures and assay conditions were previously described.^17^ mP values were calculated using the equation below:
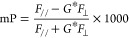
where *F*_//_ and *F*_⊥_ are the parallel and perpendicular fluorescence intensities, respectively, and *G* is the grating factor of the instrument, which was calibrated so that 20 nM 5-TAMRA in water has an mP shift of 50. The obtained mP data were fitted in the one-site-specific binding model implemented in GraphPad Prism 9.4.1 (GraphPad Software, Inc.) to give the *K*_d_ value using the equation below:
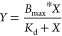
where *X*, *Y*, and *B*_max_ are the protein concentration, binding response (mP shift), and maximum binding response, respectively.

### FP-Based Binding Assay for Viral Macrodomains

The general procedure and assay conditions were same as previously described.^17^ The final protein and tracer concentrations for different protein–tracer pairs used in this study are listed in Table 2. The relative percent binding of the tracer was calculated as follows:
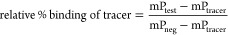
where mP_test_, mP_tracer_, and mP_neg_ are the mP values of the test wells, tracer control wells, and negative control wells, respectively. The obtained data were then fitted into an IC_50_ curve using the sigmoidal four-parameter logistic model implemented in GraphPad Prism 9.4.1 (GraphPad Software, Inc.) using the equation below:
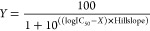
where *X* and *Y* are the inhibitor concentration and relative percent binding of the tracer, respectively.

**Table 2:** Final Concentrations for Different Protein–Tracer Pairs Used in the FP-Based Binding Assay

	**TAMRA-ADPr**	**TAMRA-NDPr**	**TAMRA-Ph-NDPr**
SARS-CoV-2 Mac1	1.5 μM protein	30 nM protein	2 nM protein
	20 nM tracer	20 nM tracer	2 nM tracer
MERS-CoV Mac1	not used	not used	20 nM protein
			20 nM tracer
VEEV Mac	not used	not used	20 nM protein
			20 nM tracer
CHIKV Mac	not used	200 nM protein	not used
		20 nM tracer	

### Co-Crystallization of SARS-CoV-2 Mac1 Bound to the NDPr Inhibitor

SARS-CoV-2 Mac1 was mixed with NDPr to final concentrations of 1.3 and 6.5 mM. The Mac1-NPDr complex was crystallized by the hanging-drop method at 20 °C by mixing 1 μL of the Mac1-NDPr solution with 1 μL of well solution (200 mM sodium acetate, 100 mM Tris–HCl pH 8, and 30% (w/v) PEG 4000). Crystals were observed after 3–5 days. Before freezing with liquid nitrogen, crystals were cryoprotected in a well solution containing 10% ethylene glycol.

### Diffraction Data Collection, Structure Solution, Model Building, and Refinement

Diffraction data was collected on beamline ID7B-2 at the Center for High-Energy X-ray Sciences (CHEXS) at the Cornell High Energy Synchrotron Source (CHESS). Initial data processing was performed using fast_dp,^35^ which uses XDS,^36^ CCP4,^37^ and CCTBX.^38^ The structure was solved by molecular replacement by Phaser^39^ in Phenix^40^ using the previously published structure of SARS-CoV-2 Mac1 (PDB: 6YWL),^11^ with the first four residues removed, as the search model. Coot^41^ was used for model building, and refinement and validation were performed in Phenix.^42^ The data collection and refinement statistics are listed in Table 3. There are three copies of the Mac1-NDPr complex in the asymmetric unit. The structures of each copy are nearly identical so noncrystallographic symmetry restraints were used during refinement. The coordinates and structure factors have been deposited in the RCSB PDB with accession code 9AZX.

**Table 3:** Data Collection and Refinement Statistics^a^

	**Mac1-NDPr**
**wavelength**	0.968600
**resolution range**	29.06–1.395 (1.43–1.395)
**space group**	P 1
**unit cell**	45.496 47.084 65.726 79.15° 72.05° 74.30°
**total reflections**	322604 (15166)
**unique reflections**	93134 (5586)
**multiplicity**	3.5 (2.7)
**completeness (%)**	94.33 (78.78)
mean I/sigma (I)	12.18 (1.38)
**Wilson***B*-factor	18.03
*R*-merge	0.05424 (0.6008)
*R*-meas	0.06424 (0.7468)
*R*-pim	0.03404 (0.4341)
**CC**1/2	0.998 (0.728)
CC*	1 (0.918)
**reflections used in refinement**	93134 (5584)
**reflections used for***R*-free	2000 (120)
*R*-work	0.1523 (0.3045)
*R*-free	0.1844 (0.3678)
**number of non-hydrogen atoms**	4310
**macromolecules**	3804
**ligands**	114
**solvent**	392
**protein residues**	503
**RMS (bonds)**	0.007
**RMS (angles)**	0.91
**Ramachandran favored (%)**	98.19
**Ramachandran allowed (%)**	1.81
**Ramachandran outliers (%)**	0.00
**Rotamer outliers (%)**	0.24
**clash score**	0.13
**average***B*-factor	27.8
**macromolecules**	26.95
**ligands**	22.25
**solvent**	38.47

a Statistics for the highest-resolution shell are shown in parentheses.

## Supporting Information Available

(Figure S1) BLI sensorgrams of MERS-CoV Mac1, VEEV Mac, and CHIKV Mac binding to immobilized biotin-labeled compounds; (Figure S2) showing IC_50_ curves of ADPr and **NDPr** binding to MacroD1 and MacroD2; (Figure S3) calculated *K*_i_ values of all inhibitors against different viral macrodomains; detailed procedure for chemical synthesis with characterization data; and NMR spectra of intermediates and target compounds (PDF)
