# Supplementary material for: GS-441524-Diphosphate-Ribose Derivatives as Nanomolar Binders and Fluorescence Polarization Tracers for SARS-CoV-2 and Other Viral Macrodomains
Source: ACS Chem Biol. 2024 Apr 22;19(5):1093–105. doi: 10.1021/acschembio.4c00027 (PMC11106745; doi:10.1021/acschembio.4c00027)
Supplement: Supplementary file 1 — cb4c00027_si_001.pdf [file cb4c00027_si_001.pdf]

Supporting Information

GS-441524-diphosphate-ribose derivatives as nanomolar binders and fluorescence polarization tracers for SARS-CoV-2 and other viral macrodomains

Kewen Peng[1], Shamar D. Wallace[2], Saket R. Bagde[2], Jialin Shang[1], Ananya Anmangandla[1], Sadhan Jana[1], J. Christopher Fromme[2,*], Hening Lin[3,*]

[1]Department of Chemistry and Chemical Biology, Cornell University, Ithaca, NY 14853, USA

[2]Department of Molecular Biology and Genetics, Weill Institute for Cell and Molecular Biology, Cornell University, Ithaca, NY 14853, USA

[3]Howard Hughes Medical Institute; Department of Chemistry and Chemical Biology; Department of Molecular Biology and Genetics, Cornell University, Ithaca, NY 14853, USA

*Correspondence: hl379@cornell.edu and jcf14@cornell.edu

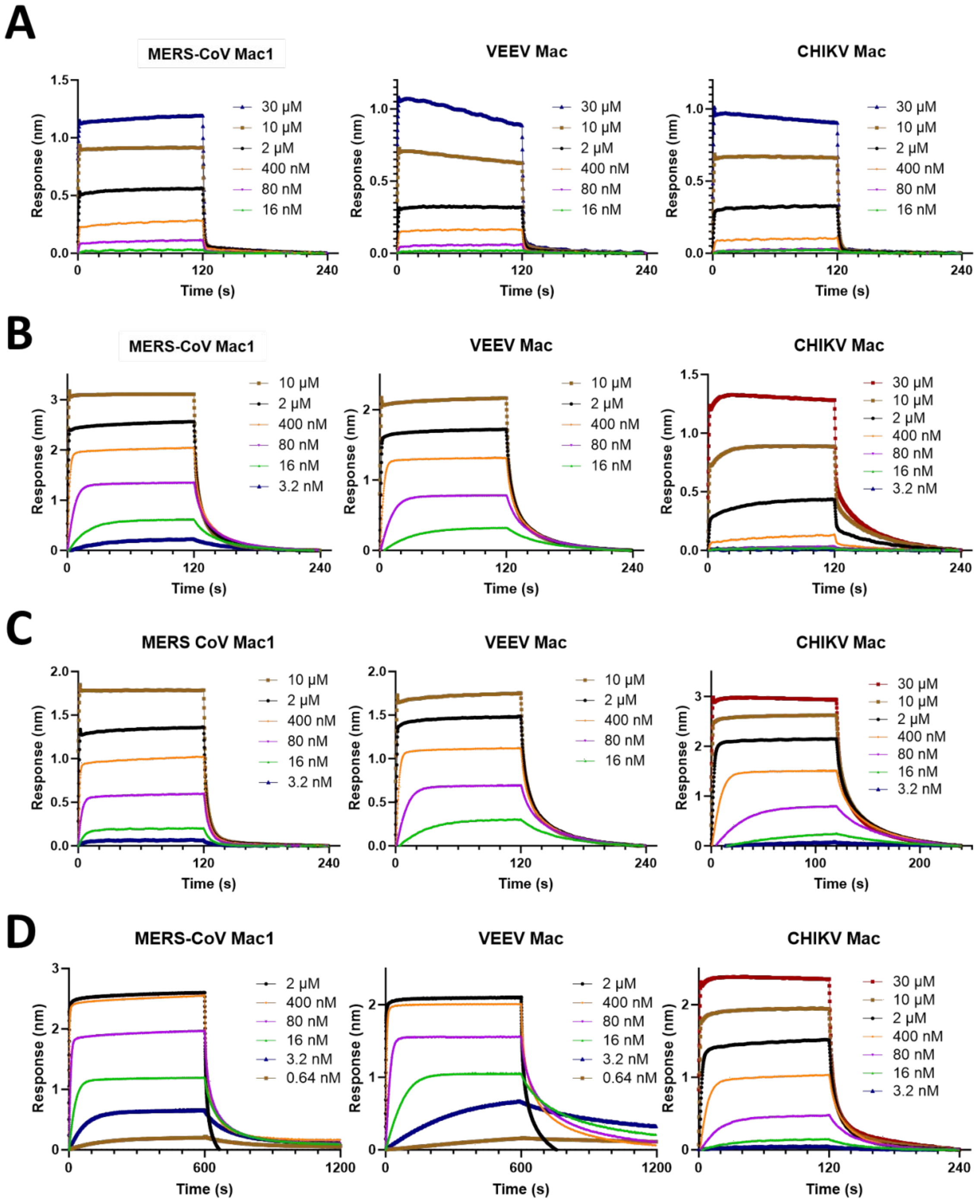

Figure S1. Biolayer interferometry sensorgrams of different viral macrodomains binding to immobilized (**A**) **biotin-ADPr**, (**B**) **biotin-Ph-ADPr**, (**C**) **biotin-NDPr,** and (**D**) **biotin-Ph-NDPr**.

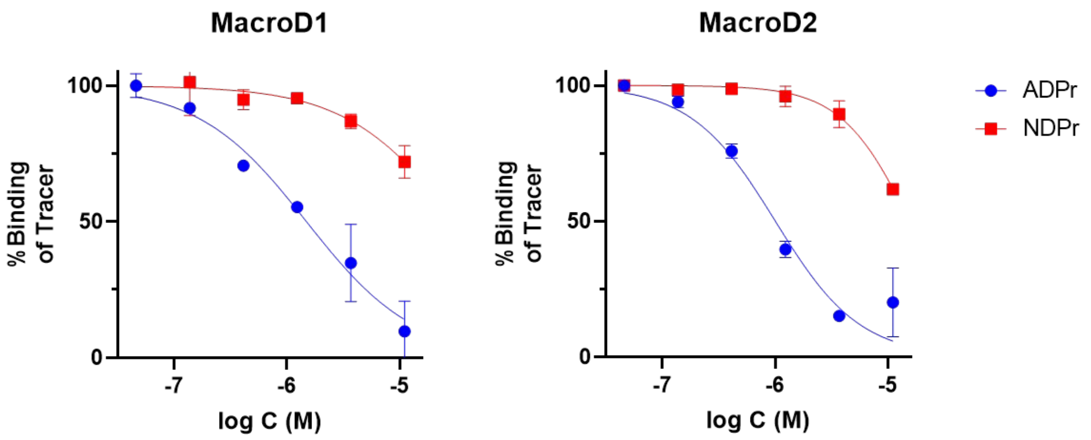

Figure S2. IC_{50} curves of ADPr and **NDPr** binding to human MacroD1 and MacroD2. Fitted IC_{50} values of ADPr binding to MacroD1 and MacroD2 are 1.5 µM and 1.0 µM, respectively, while the IC_{50} values of **NDPr** are >10 µM.

A

K_i (µM)	TAMRA-ADPr	TAMRA-NDPr	TAMRA-Ph-NDPr
**ADPr**	11.9 (8.7, 16.5)	8.07 (5.38, 12.1)	11.0 (9.2, 13.2)
**Ph-ADPr**	2.45 (1.69,3.56)	0.28 (0.25, 3.19)	0.61 (0.47, 0.80)
**NDPr**	0.91 (0.66, 1.26)	0.068 (0.061, 0.076)	0.13 (0.11, 0.16)
**Ph-NDPr**	1.52 (0.95, 2.44)	0.0075 (0.0058, 0.0097)	0.0015 (0.0013, 0.0018)

B

K_i (µM)	MERS-CoV Mac1	VEEV Mac	CHIKV Mac
**ADPr**	4.61 (3.34, 6.35)	6.49 (4.37, 9.68)	27.8 (23.3, 33.3)
**Ph-ADPr**	0.53 (0.39, 0.73)	1.87 (1.45, 2.42)	230 (182, 297)
**NDPr**	0.73 (0.61, 0.87)	0.15 (0.13, 0.16)	0.77 (0.69, 0.87)
**Ph-NDPr**	0.10 (0.09, 0.12)	0.059 (0.048, 0.072)	7.44 (5.46, 10.16)

C

K_i (µM)	SARS-CoV-2 Mac1	MERS-CoV Mac1	VEEV Mac	CHIKV Mac
**Biotin-ADPr**	1.80 (1.44, 2.24)	2.89 (2.32, 3.60)	8.68 (6.05, 12.67)	33.3 (27.2, 41.0)
**Biotin-Ph-ADPr**	0.061 (0.051, 0.072)	1.19 (1.01, 1.40)	0.50 (0.40, 0.62)	>>10
**Biotin-NDPr**	0.022 (0.019, 0.026)	0.42 (0.35, 0.49)	0.19 (0.16, 0.22)	0.92 (0.79, 1.08)
**Biotin-Ph-NDPr**	0.0031 (0.0023, 0.0042)	0.032 (0.018, 0.054)	0.022 (0.019, 0.026)	0.96 (0.80, 1.15)

Figure S3. K_i values calculated with data from (**A**) Figure 5F, (**B**) Figure 7D and (**C**) Figure 7E.

Supplementary Methods

Plasmid information for MERS-CoV Macro1 (aa1110-1276 of pp1a, K9N638)

PLSNFEHKVITECVTIVLGDAIQVAKCYGESVLVNAANTHLKHGGGIAGAINAASKGAVQKESDEYILA
KGPLQVGDSVLLQGHSLAKNILHVVGPDARAKQDVSLLSKCYKAMNAYPLVVTPLVSAGIFGVKPAV
SFDYLIREAKTRVLVVVNSQDVYKSLTIVDI*

CCACTTAGTAATTTTGAACACAAAGTTATTACTGAATGCGTGACGATCGTCCTTGGCGACGCTATC
CAAGTCGCAAAATGTTATGGGGAGAGTGTGTTGGTGAACGCAGCAAATACTCATCTTAAGCACGG
AGGAGGCATAGCCGGGGCTATCAATGCCGCTTCTAAAGGTGCAGTCCAGAAAGAGAGCGATGAGT
ACATCCTGGCGAAAGGTCCCCTTCAAGTTGGCGACTCGGTATTGCTTCAGGGTCACAGTCTCGCTA
AGAATATATTACACGTTGTTGGCCCCGATGCGCGGGCCAAACAGGACGTTAGTCTGTTGTCAAAGT
GTTACAAAGCGATGAACGCATATCCGCTGGTAGTAACGCCCCTGGTTTCGGCTGGTATATTTGGCG
TCAAGCCTGCTGTAAGCTTCGACTACCTGATACGAGAGGCCAAGACCCGTGTCCTCGTTGTCGTTA
ACTCACAGGACGTCTATAAGTCACTCACCATCGTCGACATTTGATGA

General synthetic methods

Unless otherwise stated, all reactions were carried out under ambient atmosphere. Reagents and solvents were purchased from Sigma Aldrich, TCI, Alfa-aesar, Combi-blocks, Chem-impex international, Ambeed, VWR, and Fisher scientific and were used as received. Thin layer chromatography (TLC) was performed on precoated glass-backed silica gel 60 F254 plates (EMD Millipore), visualized by UV or staining with 10% H_2SO_4 in ethanol. SiliaFlash® P60 (SiliCycle) silica gel was used for column chromatography purification. All unreported compounds were characterized by ^{1}H-NMR, ^{13}C-NMR and/or ^{31}P-NMR spectra recorded on a 500MHz Bruker spectrometer. Mass spectra (MS) were obtained on a LCQ Fleet Mass Spectrometer (Thermo Scientific) equipped with an electrospray ionization (ESI) source connected to a Shimadzu HPLC LC20-AD. Preparative HPLC was run on a Hypersil C18 column (5 µm, 30 mm * 150 mm) with water (0.1% formic acid) as solvent A and acetonitrile (0.1% formic acid) as solvent B, following a linear gradient of solvent B from 0% to 50% in 45 minutes for TAMRA-labeled ADPr derivatives and from 0% to 40% in 45 minutes for biotin-labeled ADPr derivatives.

General Procedure A for the tosylation reaction of ribose derivatives:

To a stirring solution of 2',3'-O-isopropylidene ribose derivative (1.66 mmol, 1.0 eq.) in dichloromethane (DCM, 20 mL) was added tosyl chloride (TsCl, 0.35 g, 1.82 mmol, 1.1 eq.) and 4-dimethylaminopyridine (DMAP, 0.22 g, 1.82 mmol, 1.1 eq.). After overnight reaction at room temperature, the solvent was removed under vacuum and the residue was purified by flash column chromatography on silica gel to give the desired product.

General Procedure B for the displacement reaction between di(tetra-*n*-butylammonium) salts of ADP analogues and 5-OTs ribose derivatives:

The di(tetra-*n*-butylammonium) salt of the ADP/NDP analogue (0.50 mmol, 1 eq.) was combined with the 5-OTs ribose derivative (0.75 mmol, 1.5 eq.) in 0.5 mL acetonitrile. The reaction mixture was stirred at room temperature overnight before being diluted with 2 mL of water and passed through a Dowex-50WX8 cation exchange resin (NH_4^+ form, 30 eq.) with water. The eluent was concentrated and purified by flash column chromatography on silica gel (*i*PrOH: H_2O: NH_3 12:1:1) to give the desired product.

General Procedure C for the removal of acetonide protection:

At 4 ℃, 10 mg of the acetonide-protected ADPr or NDPr analogue was added into a centrifuge tube, followed by the addition of 200 μL of cold 1N HCl. The reaction mixture was allowed to stir for 24 hours at this temperature and was then directly loaded onto a size-exclusion chromatography column (HW-40 resin) and eluted with pure water. The fractions containing the desired product were pooled and lyophilized to yield the pure product.

General Procedure D for the click reaction of azido-ADPr/NDPr analogues:

The azido-ADPr/NDPr analogue (1.3 mg, 2.2 μmol, 1 eq.) in 200 μL water was added to a microcentrifuge tube, followed by the addition of 50 mg/mL $CuSO_4$ (5.7 μL, 1.8 μmol, 0.8 eq.), 100 mg/mL sodium ascorbate (6.9 μL, 3.5 μmol, 1.6 eq.), 10 μL saturated $NaHCO_3$ solution and finally **TAMRA-alkyne** (1.3 mg, 2.2 μmol, 1 eq.) or biotin-PEG4-alkyne (1.0 mg, 2.2 μmol, 1 eq.) in 90 μL DMF. The reaction mixture was incubated at room temperature with

occasional vortexing. After 30 minutes, the reaction was complete based on LC-MS analysis. The reaction mixture was diluted with 1 mL water and centrifugated at 21,000 g. The supernatant was directly purified by prep-HPLC. The desired fractions were pooled and freeze-dried to yield the pure product. For TAMRA-labeled compounds, additional cation-exchange was performed with Dowex-50WX8 ion-exchange resin (NH_4^+ form) to give the ammonium salts of the target compounds with better water solubility and improved NMR peak shapes.

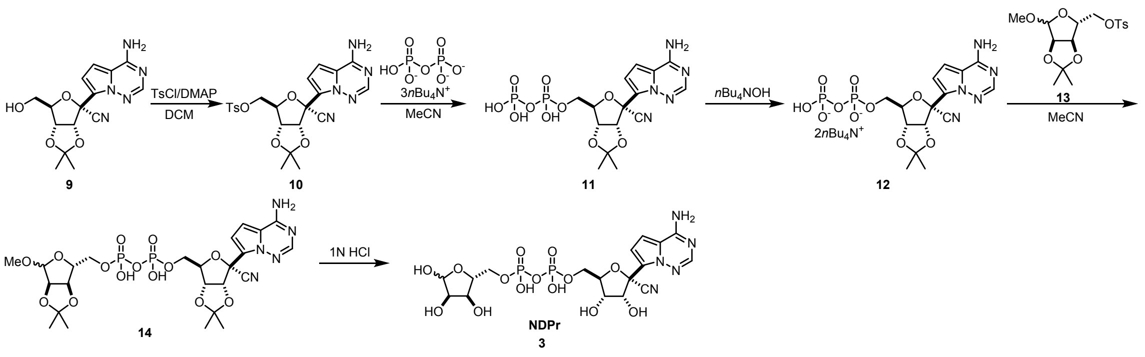

2',3'-O-isopropylidene-5'-O-tosyl-nucleoside (10)

General Procedure A was followed. **10** was obtained as an off-white solid (1.34 g, yield: 91%)

LC-MS (ESI): m/z calcd for $C_{22}H_{24}N_5O_6S^+$ $[M+H]^+$ 486.1, found 486.2.

^{1}H NMR (500 MHz, $CDCl_3$) δ 7.96 (d, J = 1.8 Hz, 1H), 7.72 (d, J = 8.4, 2H), 7.25 (d, J = 8.0 Hz, 2H), 6.99 (dd, J = 4.7, 1.9 Hz, 1H), 6.63 (dd, J = 4.7, 1.9 Hz, 1H), 5.54 (s, 2H), 5.45 (dd, J = 6.8, 1.9 Hz, 1H), 4.96 (dd, J = 6.7, 3.9 Hz, 1H), 4.55 (q, J = 4.4 Hz, 1H), 4.37 – 4.24 (m, 2H), 2.42 (s, 3H), 2.07 (d, J = 1.9 Hz, 1H), 1.76 (s, 3H), 1.41 (s, 3H).

^{13}C NMR (126 MHz, $CDCl_3$) δ 155.2, 147.3, 145.2, 132.3, 129.8, 128.0, 123.1, 117.3, 116.7, 115.5, 112.6, 99.9, 83.8, 82.3, 81.6, 81.4, 77.3, 77.0, 76.8, 68.1, 26.3, 25.5, 21.7.

2',3'-O-isopropylidene-NDP (11)

Tosylate **2** (0.43 g, 0.89 mmol, 1.0 eq.) and pyrophosphoric acid tris(tetrabutylammonium) salt (1.00 g, 1.11 mmol, 1.25 eq.) were dissolved in acetonitrile (1 mL). The reaction mixture was stirred at room temperature for 24 hours before being diluted with water (5 mL). The mixture was filtered and the filtrate was passed through a column of Dowex-50WX8 cation exchange resin (NH_4^+ form, 30 eq.) with water to remove tetra-*n*-butylammonium. The eluent containing the product was evaporated to dryness and purified on a silica gel column (*i*PrOH:NH_3:H_2O 8:1:1 to 4:1:1) to give **11** as a white solid (105 mg, yield: 24%)

LC-MS (ESI): m/z calcd for $C_{15}H_{18}N_5O_{10}P_2^-$ $[M-H]^-$ 490.1, found 490.3.

^{1}H NMR (500 MHz, D_2O) δ 7.93 (s, 1H), 7.02 (d, J = 4.7 Hz, 1H), 6.94 (d, J = 4.7 Hz, 1H), 5.51 (d, J = 6.5 Hz, 1H), 5.26 (dd, J = 6.5, 2.6 Hz, 1H), 4.78 (1H, overlapped with the solvent peak), 4.19 – 4.06 (m, 2H), 1.77 (s, 3H), 1.50 (s, 3H).

^{13}C NMR (126 MHz, D_2O) δ 155.5, 147.1, 122.5, 117.1, 116.8, 116.1, 111.1, 102.4, 84.5, 83.9,

83.9, 81.5, 80.1, 64.7, 64.7, 25.0, 24.3.

^{31}P NMR (202 MHz, D_2O) δ -7.72 (d, J = 20.6 Hz), -9.02 (d, J = 20.3 Hz).

2',3'-O-isopropylidene NDP di(tetra-n-butylammonium) salt (12)

11 (105 mg, 0.216 mmol, 1 eq.) was dissolved in 1 mL of water, followed by the addition of 55% *n*Bu_4NOH in water (202 μL, 2 eq.). The resulting mixture was lyophilized and the obtained solid was directly used in the next step.

2',3': 2'',3''-bis-O-isopropylidene-NDP-ribose (14)

General Procedure B was followed. **12** (210 mg, 0.216 mmol) was reacted with **13** (106 mg, 0.324 mmol) to give **14** (10 mg, yield: 7%).

LC-MS (ESI): m/z calcd for $C_{24}H_{32}N_5O_{14}P_2^-$ $[M-H]^-$ 676.1, found 676.4.

^{1}H NMR (500 MHz, D_2O) δ 8.00 (s, 1H), 7.18 (d, J = 4.8 Hz, 1H), 7.07 (d, J = 4.8 Hz, 1H), 5.40 (d, J = 6.2 Hz, 1H), 5.24 (dd, J = 6.3, 2.3 Hz, 1H), 4.98 (d, J = 1.3 Hz, 1H), 4.81 (s, 1H), 4.80 (s, 1H), 4.61 (d, J = 5.8 Hz, 1H), 4.26 (t, J = 7.1 Hz, 1H), 4.15 (s, 2H), 3.90 – 3.78 (m, 2H), 3.29 (s, 3H), 1.76 (s, 3H), 1.48 (s, 3H), 1.44 (s, 3H), 1.30 (s, 3H).

^{13}C NMR (126 MHz, D_2O) δ 151.0, 139.7, 126.5, 117.0, 115.8, 114.8, 113.1, 112.6, 108.5, 107.4, 85.1, 84.9, 84.8, 84.2, 81.8, 81.0, 80.7, 65.9, 65.2, 54.8, 25.3, 25.0, 24.3, 23.7.

^{31}P NMR (202 MHz, D_2O) δ -11.53.

NDP-ribose (3)

General Procedure C was followed. **14** (10 mg, 0.014 mmol) was stirred in 200 μL 1N HCl to give **3** (8 mg, yield: quant.).

LC-MS (ESI): m/z calcd for $C_{17}H_{22}N_5O_{14}P_2^-$ $[M+H]^-$ 582.1, found 582.2.

^{1}H NMR (500 MHz, D_2O) δ 8.08 (s, 1H), 7.37 (d, J = 4.9 Hz, 1H), 7.17 (d, J = 4.8 Hz, 1H), 5.32 (d, J = 4.1 Hz, 0.4H, H1-α), 5.18 (d, J = 2.1 Hz, 0.6H, H1-β), 4.94 (s, 1H), 4.52 (s, 1H), 4.45 (d, J = 4.6 Hz, 1H), 4.25 (q, J = 5.7 Hz, 1H), 4.16 (q, J = 4.4 Hz, 3H), 4.12 – 4.00 (m, 2H), 3.97 (dd, J = 5.0, 2.1 Hz, 2H).

^{13}C NMR (126 MHz, D_2O) δ 148.9, 135.7, 128.4, 116.4, 113.9, 113.5, 109.9, 101.2, 96.4, 84.8,

81.1, 77.5, 75.4, 75.2, 70.7, 70.4, 70.3, 70.0, 66.3, 64.6.

^{31}P NMR (202 MHz, D_2O) δ -11.18.

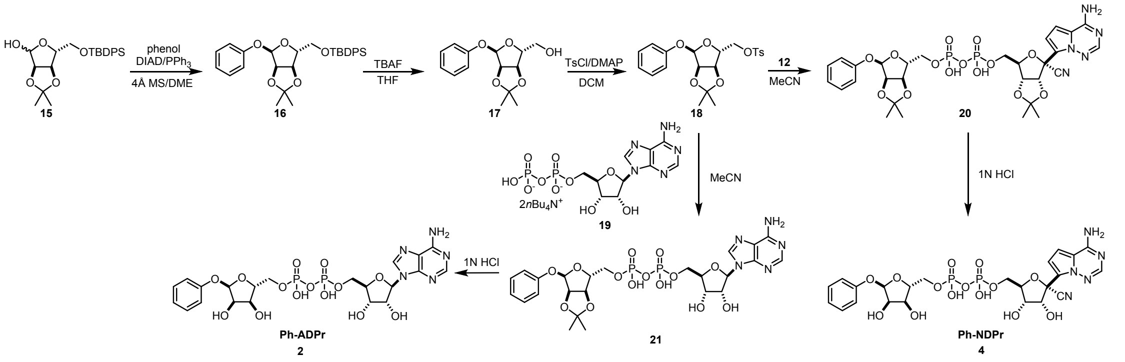

α-1-OPh-2,3-O-isopropylidene-5-OTBDPS-D-ribose (16)

To a flask was added **15** (200 mg, 0.47 mmol, 1 eq.), phenol (44 mg, 0.47 mmol, 1 eq.), PPh_3 (184 mg, 0.70 mmol, 1.5 eq.), 4 Å molecular sieve (100 mg) and DME (5 mL). The resulting suspension was stirred for 10 minutes, followed by the slow addition of diisopropyl azodicarboxylate (DIAD,138 µL, 0.70 mmol, 1.5 eq.). The reaction was complete after 2 hours with the α-isomer as the major product. Hexanes (10 mL) were added and the mixture was stirred for an additional 10 minutes. The precipitates were filtered off and the filtrate was evaporated to dryness. Column chromatography on silica gel (hexanes:ethyl acetate 8:1) afforded the product **16** as a colorless oil (126 mg, yield: 58%).

^{1}H NMR (500 MHz, $CDCl_3$) δ 7.72 (ddt, J = 12.5, 6.6, 1.5 Hz, 4H), 7.51 – 7.46 (m, 2H), 7.45 – 7.41 (m, 4H), 7.32 (tt, J = 7.6, 2.2 Hz, 2H), 7.15 – 7.09 (m, 2H), 7.04 (tt, J = 7.3, 1.1 Hz, 1H), 5.77 (d, J = 4.4 Hz, 1H), 4.94 (dd, J = 6.5, 4.3 Hz, 1H), 4.87 (dd, J = 6.4, 1.8 Hz, 1H), 4.41 (q, J = 2.6 Hz, 1H), 3.89 (dd, J = 11.3, 3.2 Hz, 1H), 3.82 (dd, J = 11.2, 2.7 Hz, 1H), 1.71 (s, 3H), 1.46 (s, 3H), 1.11 (s, 9H).

^{13}C NMR (126 MHz, $CDCl_3$) δ 157.2, 135.6, 135.6, 133.0, 132.9, 130.0, 129.9, 129.4, 127.9, 127.8, 122.0, 116.8, 114.6, 100.5, 82.6, 81.1, 81.0, 64.8, 26.9, 26.1, 25.9, 21.6, 19.2.

α-1-OPh-2,3-O-isopropylidene-D-ribose (17)

16 (2.74 g, 5.90 mmol, 1 eq.) was dissolved in 15 mL THF and 1 M TBAF in THF (6.49 mL, 6.49 mmol, 1.1 eq.) was added. The reaction mixture was stirred at room temperature for 2

hours. Purification by column chromatography (hexanes:ethyl acetate 2:1) afforded the product **17** as a low-melting point white solid (0.98 g, yield: 73%).

^{1}H NMR (500 MHz, $CDCl_3$) δ 7.40 – 7.24 (m, 2H), 7.09 (d, J = 2.2 Hz, 2H), 7.04 (td, J = 7.4, 2.3 Hz, 1H), 5.72 – 5.63 (m, 1H), 4.93 – 4.70 (m, 2H), 4.38 (q, J = 3.0 Hz, 1H), 3.86 (dd, J = 12.2, 3.3 Hz, 1H), 3.76 (d, J = 12.1 Hz, 1H), 2.12 – 2.00 (m, 1H), 1.71 (s, 3H), 1.44 (s, 3H).

^{13}C NMR (126 MHz, $CDCl_3$) δ 156.9, 129.4, 122.2, 116.9, 115.8, 99.8, 82.6, 81.1, 80.4, 62.7, 26.0, 26.0.

α-1-OPh-2,3-O-isopropylidene-5-OTs-D-ribose (18)

General Procedure A was followed. **17** (0.67 g, 2.96 mmol) was reacted to give **18** as a white syrup (0.78 g, yield: 63%).

^{1}H NMR (500 MHz, $CDCl_3$) δ 7.82 (d, J = 7.9 Hz, 2H), 7.38 (d, J = 7.9 Hz, 2H), 7.31 – 7.27 (m, 2H), 7.04 (d, J = 7.3 Hz, 1H), 7.01 (d, J = 8.2 Hz, 2H), 5.51 (d, J = 4.4 Hz, 1H), 4.82 (dd, J = 6.7, 4.5 Hz, 1H), 4.71 (dd, J = 6.7, 2.5 Hz, 1H), 4.42 (q, J = 2.9 Hz, 1H), 4.23 (t, J = 2.7 Hz, 2H), 2.48 (s, 3H), 1.67 (s, 3H), 1.41 (s, 3H).

^{13}C NMR (126 MHz, $CDCl_3$) δ 156.8, 145.3, 132.6, 130.0, 129.4, 128.0, 122.3, 116.7, 115.7, 99.9, 80.7, 80.2, 79.5, 69.6, 53.4, 25.9, 21.7.

α-1''-OPh-2',3':2'',3''-bis-O-isopropylidene-NDP-ribose (20)

General Procedure B was followed. **18** (127 mg, 0.30 mmol) was reacted with **12** to give **20** as a white solid (12 mg, yield: 5%).

LC-MS (ESI): m/z calcd for $C_{29}H_{34}N_5O_{14}P_2^-$ [M-H]$^-$ 738.2, found 738.3.

^{1}H NMR (500 MHz, D_2O) δ 7.90 (s, 1H), 7.24 (t, J = 8.5 Hz, 2H), 7.08 (d, J = 4.8 Hz, 1H), 7.01 (d, J = 4.7 Hz, 1H), 6.99 – 6.93 (m, 3H), 5.72 (d, J = 4.0 Hz, 1H), 5.17 (d, J = 2.4 Hz, 2H), 5.00 (dd, J = 6.2, 4.1 Hz, 1H), 4.96 (dd, J = 6.3, 1.5 Hz, 1H), 4.75 (1H, overlapped with the solvent peak) 4.36 (s, 1H), 4.13 (q, J = 5.3 Hz, 2H), 4.07 – 3.99 (m, 2H), 1.74 (s, 3H), 1.56 (s, 3H), 1.47 (s, 3H), 1.40 (s, 3H).

^{13}C NMR (126 MHz, D_2O) δ 155.8, 151.2, 140.2, 129.7, 126.0, 122.6, 117.1, 116.9, 116.2, 115.7, 114.8, 114.5, 112.4, 107.1, 100.5, 85.1, 84.2, 84.2, 81.6, 80.7, 80.7, 80.6, 79.5, 66.6,

65.1, 25.1, 24.9, 24.3.

^{31}P NMR (202 MHz, D_2O) δ -11.34 (d, J = 19.3 Hz), -11.66 (d, J = 18.5 Hz)

α-1''-OPh-NDP-ribose (4)

General Procedure C was followed. **20** (2.7 mg, 3.7 μmol) was stirred in 100 μL of 1N HCl to give **4** as a white solid (2.0 mg, yield: 82%).

LC-MS (ESI): m/z calcd for $C_{23}H_{26}N_5O_{14}P_2^-$ [M-H]$^-$ 658.1, found 658.3.

^{1}H NMR (500 MHz, D_2O) δ 7.89 (s, 1H), 7.28 – 7.21 (m, 2H), 7.06 – 7.00 (m, 2H), 6.98 (d, J = 4.7 Hz, 1H), 6.96 – 6.90 (m, 2H), 5.44 (d, J = 4.2 Hz, 1H), 4.86 (d, J = 5.3 Hz, 1H), 4.51 – 4.45 (m, 1H), 4.41 (t, J = 4.9 Hz, 1H), 4.32 – 4.27 (m, 1H), 4.24 (d, J = 5.3 Hz, 2H), 4.16 (q, J = 4.6 Hz, 2H), 4.03 – 3.94 (m, 2H).

^{13}C NMR (126 MHz, D_2O) δ 156.2, 142.9, 129.7, 125.0, 122.7, 116.8, 116.7, 115.5, 111.9, 105.2, 100.7, 84.3, 84.3, 84.1, 84.0, 77.4, 75.0, 71.1, 70.0, 69.7, 65.5, 64.5.

^{31}P NMR (202 MHz, D_2O) δ -11.11 (d, J = 21.2 Hz), -11.41 (d, J = 21.8 Hz).

α-1''-OPh-2'',3''-O-isopropylidene-ADP-ribose (21)

General Procedure B was followed. **18** (210 mg, 0.50 mmol) was reacted with the di(tetra-*n*-butylammonium) salt of ADP (**19**) to give **21** as a white solid (30 mg, yield: 9%)

LC-MS (ESI): m/z calcd for $C_{24}H_{30}N_5O_{14}P_2^-$ [M-H]$^-$ 674.1, found 674.5.

^{1}H NMR (500 MHz, D_2O) δ 8.55 (s, 1H), 8.24 (s, 1H), 7.26 – 7.15 (m, 2H), 6.95 (t, J = 7.4 Hz, 1H), 6.93 – 6.87 (m, 2H), 6.06 (d, J = 5.3 Hz, 1H), 5.71 (d, J = 4.4 Hz, 1H), 5.03 (dd, J = 6.2, 4.0 Hz, 1H), 4.97 (dd, J = 6.2, 1.5 Hz, 1H), 4.64 (t, J = 5.2 Hz, 1H), 4.47 (t, J = 4.6 Hz, 1H), 4.43 (s, 1H), 4.37 (q, J = 3.3 Hz, 1H), 4.24 (q, J = 12.7, 12.1 Hz, 2H), 4.09 (d, J = 4.0 Hz, 2H), 1.54 (s, 3H), 1.38 (s, 3H).

^{13}C NMR (126 MHz, D_2O) δ 155.7, 152.1, 147.9, 141.3, 129.7, 122.6, 116.1, 114.5, 100.5, 87.7, 84.0, 80.7, 79.5, 74.7, 70.2, 66.6, 65.1, 24.8, 24.3.

^{31}P NMR (202 MHz, D_2O) δ -11.16.

α-1''-OPh-ADP-ribose (2)

General Procedure C was followed. **21** (13 mg, 0.019 mmol) was stirred in 400 μL 1N HCl to give **2** as a white solid (10 mg, yield: 83%).

LC-MS (ESI): m/z calcd for $C_{21}H_{26}N_5O_{14}P_2^-$ [M-H]$^-$ 634.1, found 634.2.

^{1}H NMR (500 MHz, D_2O) δ 8.44 (s, 1H), 8.16 (s, 1H), 7.25 – 7.17 (m, 2H), 6.96 (t, J = 7.4 Hz, 1H), 6.93 – 6.89 (m, 2H), 6.05 (d, J = 5.5 Hz, 1H), 5.47 (d, J = 4.4 Hz, 1H), 4.62 (d, J = 5.4 Hz, 1H), 4.47 (t, J = 4.5 Hz, 1H), 4.38 – 4.33 (m, 1H), 4.31 (dd, J = 6.2, 4.4 Hz, 2H), 4.25 (dd, J = 6.1, 2.9 Hz, 1H), 4.21 (dd, J = 5.0, 3.0 Hz, 2H), 4.04 (dd, J = 5.3, 3.6 Hz, 2H).

^{13}C NMR (126 MHz, D_2O) δ 156.2, 155.6, 152.8, 148.9, 139.7, 129.6, 122.8, 117.1, 101.1, 87.0, 84.1, 83.7, 74.5, 71.1, 70.2, 69.7, 65.6, 65.1.

^{31}P NMR (202 MHz, D_2O) δ -11.07 (d, J = 21.6 Hz), -11.32 (d, J = 21.2 Hz).

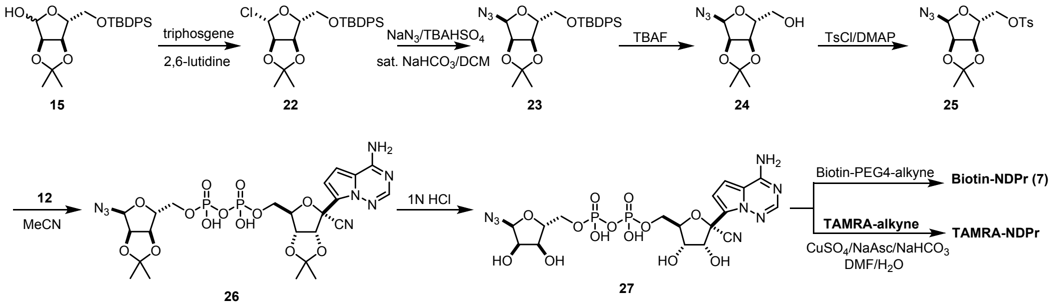

α-1-azido-1-deoxy-2,3-O-isopropylidene-5-OTBSPS-D-ribose (23)

15 (0.50 g, 1.17 mmol, 1 eq.) was dissolved with 10 mL of THF and cooled on an ice bath. Triphosgene (0.18 g, 0.59 mmol, 0.5 eq.) was added, followed by the slow addition of 2,6-lutidine (219 μL, 1.6 eq.). The reaction mixture was then removed from the ice bath and stirred at room temperature for 2 hours. The formed precipitates were filtered out and the filtrate was evaporated to dryness and taken up with 15 mL of ethyl acetate, washed with ice-cold water (20 mL, twice) and brine. The organic layer was dried over Na_2SO_4 and evaporated to dryness to give crude **22** (0.38 g) that was used in the next step without purification. The α and β-isomers were formed in a 3:7 ratio as indicated by the NMR integrations of the anomeric hydrogen in the crude sample. To the flask with crude **22** was added 5 mL of DCM, sodium azide (0.28 g, 4.25 mmol, 5 eq.) in 5 mL of saturated $NaHCO_3$ and Bu_4NHSO_4 (0.29 g, 0.85 mmol, 1 eq.). The resulting bi-phasic solution was stirred vigorously overnight. The reaction

mixture was diluted with 10 mL of DCM and the organic layer was washed water and brine, dried over anhydrous Na_2SO_4 and evaporated to dryness. The residue was purified by column chromatography (hexanes:ethyl acetate 20:1) to give diastereomerically pure **23** as a colorless oil (0.28 g, two-step yield: 53%)

^{1}H NMR (500 MHz, $CDCl_3$) δ 7.68 – 7.64 (m, 4H), 7.49 – 7.42 (m, 6H), 5.22 (d, J = 4.2 Hz, 1H), 4.86 (dd, J = 6.2, 1.2 Hz, 1H), 4.80 (dd, J = 6.2, 4.2 Hz, 1H), 4.31 (q, J = 2.3 Hz, 1H), 3.90 (dd, J = 11.3, 2.7 Hz, 1H), 3.73 (dd, J = 11.3, 2.5 Hz, 1H), 1.63 (s, 3H), 1.41 (s, 3H), 1.08 (s, 9H).

^{13}C NMR (126 MHz, $CDCl_3$) δ 135.6, 135.5, 132.5, 132.4, 130.1, 130.0, 128.0, 128.0, 113.7, 91.3, 83.3, 81.9, 81.7, 65.8, 26.9, 25.6, 24.6, 19.1

α-1-azido-1-deoxy-2,3-O-isopropylidene-D-ribose (24)

23 (0.28 g, 0.62 mmol, 1 eq.) was dissolved in 15 mL of THF and 1 M TBAF in THF (0.68 mL, 0.68 mmol, 1.1 eq.) was added. The reaction mixture was stirred at room temperature for 2 hours. Purification by column chromatography (hexanes:ethyl acetate 2:1) afforded the product **24** as a colorless oil (0.11 g, yield: 83%).

^{1}H NMR (500 MHz, $CDCl_3$) δ 5.20 (d, J = 4.1 Hz, 1H), 4.81 – 7.42 (m, 2H), 4.34 (td, J = 3.5, 2.0 Hz, 1H), 3.87 (ddd, J = 11.8, 5.1, 3.1 Hz, 1H), 3.75 (ddd, J = 11.8, 5.9, 3.8 Hz, 1H), 1.70 (t, J = 5.5 Hz, 1H), 1.64 (s, 3H), 1.40 (s, 3H).

^{13}C NMR (126 MHz, $CDCl_3$) δ 114.8, 91.0, 83.3, 81.5, 81.0, 63.3, 25.7, 24.9.

α-1-azido-1-deoxy-2,3-O-isopropylidene-5-OTs-D-ribose (25)

General Procedure A was followed. **23** (93 mg, 0.43 mmol) was reacted to give **25** as a colorless oil (120 mg, yield: 76%).

^{1}H NMR (500 MHz, $CDCl_3$) δ 7.80 (d, J = 8.0 Hz, 2H), 7.40 (d, J = 8.0 Hz, 2H), 4.98 (t, J = 2.2 Hz, 1H), 4.74 (d, J = 2.3 Hz, 2H), 4.38 (t, J = 2.9 Hz, 1H), 4.20 (dd, J = 10.8, 2.9 Hz, 1H), 4.14 (dd, J = 10.8, 3.0 Hz, 1H), 2.49 (s, 3H), 1.59 (s, 3H), 1.37 (s, 3H).

^{13}C NMR (126 MHz, $CDCl_3$) δ 145.5, 132.2, 130.1, 127.9, 114.7, 91.2, 81.2, 81.1, 80.3, 70.3, 25.6, 24.8, 21.7.

α-1''-azido-1''-deoxy-2',3':2'',3''-bis-O-isopropylidene-NDP-ribose (26)

General Procedure B was followed. **25** (120 mg, 0.33 mmol) was reacted with **12** to give **26** as a white solid (25 mg, yield: 11%).

LC-MS (ESI): m/z calcd for $C_{23}H_{29}N_8O_{13}P_2^-$ $[M-H]^-$ 687.1, found 687.4.

^{1}H NMR (500 MHz, D_2O) δ 7.80 (s, 1H), 6.86 (d, J = 4.1 Hz, 1H), 6.82 (d, J = 4.9 Hz, 1H), 5.32 (d, J = 6.4 Hz, 1H), 5.11 (dd, J = 6.6, 2.8 Hz, 1H), 5.08 (d, J = 4.0 Hz, 1H), 4.76 (d, J = 6.1 Hz, 1H), 4.67 – 4.62 (m, 2H), 4.09 – 4.01 (m, 3H), 3.83 (s, 2H), 1.64 (s, 3H), 1.39 (s, 6H), 1.19 (s, 3H).

^{13}C NMR (126 MHz, D_2O) δ 153.8, 144.8, 123.3, 117.3, 116.0, 115.9, 113.8, 111.6, 103.8, 90.8, 84.4, 83.7, 81.2, 81.2, 81.1, 81.1, 80.3, 79.9, 66.7, 64.8, 64.2, 24.9, 24.4, 24.2, 23.4.

^{31}P NMR (202 MHz, D_2O) δ -11.63

α-1''-azido-1''-deoxy-NDP-ribose (27)

General procedure C was followed. **26** (10 mg, 0.014 mmol) was stirred in 100 μL of 1N HCl to yield **27** as a white solid (9 mg, yield: quant.).

LC-MS (ESI): m/z calcd for $C_{17}H_{21}N_8O_{13}P_2^-$ $[M-H]^-$ 608.1, found 608.2.

^{1}H NMR (500 MHz, D_2O) δ 8.06 (s, 1H), 7.30 (d, J = 4.8 Hz, 1H), 7.14 (d, J = 4.9 Hz, 1H), 5.39 (d, J = 4.7 Hz, 1H), 4.93 (d, J = 5.2 Hz, 1H), 4.51 (d, J = 2.7 Hz, 1H), 4.42 (t, J = 4.9 Hz, 1H), 4.25 (t, J = 5.1 Hz, 1H), 4.20 – 4.12 (m, 4H), 3.97 (dq, J = 6.4, 3.5, 3.1 Hz, 2H).

^{13}C NMR (126 MHz, D_2O) δ 150.1, 137.9, 127.4, 116.5, 114.5, 113.0, 108.6, 91.4, 84.7, 84.6, 84.0, 83.9, 77.5, 75.2, 71.6, 70.2, 69.7, 65.4, 65.3, 64.7.

^{31}P NMR (202 MHz, D_2O) δ -11.16 (d, J = 20.6 Hz), -11.40 (d, J = 20.7 Hz).

Biotin-NDP-ribose (7)

General Procedure D was followed. **26** (2.0 mg, 3.3 μmol) was reacted with biotin-PEG4-alkyne to yield **7** as a white solid (2.6 mg, yield: 74%).

LC-MS (ESI): m/z calcd for $C_{38}H_{58}N_{11}O_{19}P_2S^+$ $[M+H]^+$ 1066.3, found 1066.3.

^{1}H NMR (500 MHz, D_2O) δ 8.24 (s, 1H), 8.06 (s, 1H), 7.33 – 7.24 (m, 1H), 7.15 (d, J = 4.6

Hz, 1H), 6.32 (d, J = 5.2 Hz, 1H), 4.91 (d, J = 5.0 Hz, 1H), 4.66 (d, J = 6.1 Hz, 3H), 4.56 (dd, J = 8.0, 4.8 Hz, 1H), 4.50 (s, 1H), 4.42 (d, J = 5.2 Hz, 3H), 4.36 (dd, J = 8.0, 4.5 Hz, 1H), 4.17 (s, 2H), 4.06 (s, 2H), 3.71 (dt, J = 15.7, 4.7 Hz, 4H), 3.68 – 3.63 (m, 8H), 3.58 (t, J = 5.3 Hz, 2H), 3.35 (t, J = 5.3 Hz, 2H), 3.26 (dd, J = 9.2, 4.8 Hz, 1H), 2.94 (dd, J = 13.0, 4.9 Hz, 1H), 2.73 (d, J = 13.0 Hz, 1H), 2.22 (t, J = 7.3 Hz, 2H), 1.68 – 1.50 (m, 4H), 1.35 (p, J = 9.1, 8.2 Hz, 2H).

^{13}C NMR (126 MHz, D_2O) δ 181.8, 176.9, 165.4, 163.2, 162.9, 149.2, 136.5, 128.1, 116.5, 113.5, 109.4, 90.0, 84.7, 84.4, 77.6, 75.2, 71.3, 70.3, 70.2, 69.7, 69.6, 69.6, 69.4, 69.0, 68.9, 65.4, 64.6, 63.2, 62.1, 60.3, 55.4, 39.7, 38.9, 35.5, 27.9, 27.7, 25.2.

TAMRA-NDPr

General Procedure D was followed. **26** (5.0 mg, 8.2 μmol) was reacted with **TAMRA-alkyne** to yield **TAMRA-NDPr** as a dark purple solid (5.8 mg, yield: 58%).

LC-MS (ESI): m/z calcd for $C_{53}H_{61}N_{12}O_{18}P_2^-$ $[M-H]^-$ 1215.4, found 1215.6.

^{1}H NMR (500 MHz, D_2O) δ 8.27 (s, 1H), 8.13 – 7.96 (m, 1H), 7.91 (s, 1H), 7.66 (s, 1H), 7.52 – 7.20 (m, 1H), 7.12 – 6.83 (m, 2H), 6.73 (s, 1H), 6.69 – 6.42 (m, 3H), 6.26 (d, J = 5.4 Hz, 1H), 6.12 (s, 1H), 6.09 – 5.92 (m, 1H), 4.77 (1H, overlapped with the solvent peak), 4.61 (t, J = 5.4 Hz, 1H), 4.50 – 4.28 (m, 4H), 4.20 – 4.05 (m, 2H), 4.00 (d, J = 28.0 Hz, 2H), 3.40 (s, 2H), 3.07 (s, 2H), 3.04 – 2.72 (m, 14H), 2.50 (t, J = 7.4 Hz, 2H), 1.53 (t, J = 6.9 Hz, 2H), 1.44 – 1.27 (m, 4H), 1.25 – 1.13 (m, 2H).

^{13}C NMR (126 MHz, D_2O) δ 174.4, 172.3, 168.5, 157.7, 156.3, 152.4, 145.8, 142.8, 135.2, 134.1, 130.5, 128.5, 127.8, 124.7, 122.6, 117.4, 116.9, 115.1, 113.5, 112.4, 111.7, 104.2, 95.6, 89.7, 89.6, 84.7, 84.2, 77.7, 77.5, 77.2, 76.9, 74.9, 71.1, 70.2, 70.0, 65.2, 64.6, 39.9, 39.0, 35.1, 28.3, 28.1, 25.8, 25.6, 21.2.

^{31}P NMR (202 MHz, D_2O) -11.30 (d, J = 26.0 Hz).

Biotin-ADPr (5)

General Procedure D was followed. ADPr-N_3 (2.0 mg, 3.4 μmol) was reacted with biotin-

PEG4-alkyne to yield **5** as a white solid (2.1 mg, yield: 55%).

LC-MS (ESI): m/z calcd for $C_{36}H_{58}N_{11}O_{19}P_2S^+$ $[M+H]^+$ 1042.3, found 1042.3.

^{1}H NMR (500 MHz, D_2O) δ 8.66 (s, 1H), 8.48 – 8.39 (m, 1H), 8.18 (s, 1H), 6.34 (d, J = 5.4 Hz, 1H), 6.15 (d, J = 5.4 Hz, 1H), 4.74 (1H, overlapped with the solvent peak), 4.68 – 4.62 (d, m, 3H), 4.57 (dd, J = 8.1, 4.6 Hz, 1H), 4.53 (dd, J = 5.0, 3.7 Hz, 1H), 4.51 – 4.47 (m, 1H), 4.44 (dt, J = 6.4, 5.0 Hz, 1H), 4.38 (dt, J = 8.1, 4.3 Hz, 2H), 4.25 (s, 2H), 4.16 (d, J = 11.5 Hz, 1H), 4.13 – 4.07 (m, 1H), 3.70 (tt, J = 6.0, 2.3 Hz, 4H), 3.67 – 3.63 (m, 8H), 3.59 (t, J = 5.2 Hz, 2H), 3.35 (t, J = 5.2 Hz, 2H), 3.30 – 3.24 (m, 1H), 2.96 (dt, J = 13.0, 5.0 Hz, 1H), 2.74 (dd, J = 13.0, 5.2 Hz, 1H), 2.23 (td, J = 7.2, 5.3 Hz, 2H), 1.74 – 1.51 (m, 4H), 1.40 – 1.34 (m, 2H).

^{13}C NMR (126 MHz, D_2O) δ 177.0, 165.4, 163.2, 162.9, 149.9, 144.7, 142.6, 124.6, 117.6, 90.0, 87.9, 84.4, 74.8, 71.3, 70.4, 70.3, 69.7, 69.6, 69.6, 69.4, 69.0, 68.9, 65.5, 65.2, 63.1, 62.1, 60.3, 55.4, 39.7, 38.9, 35.5, 27.9, 27.7, 25.2.

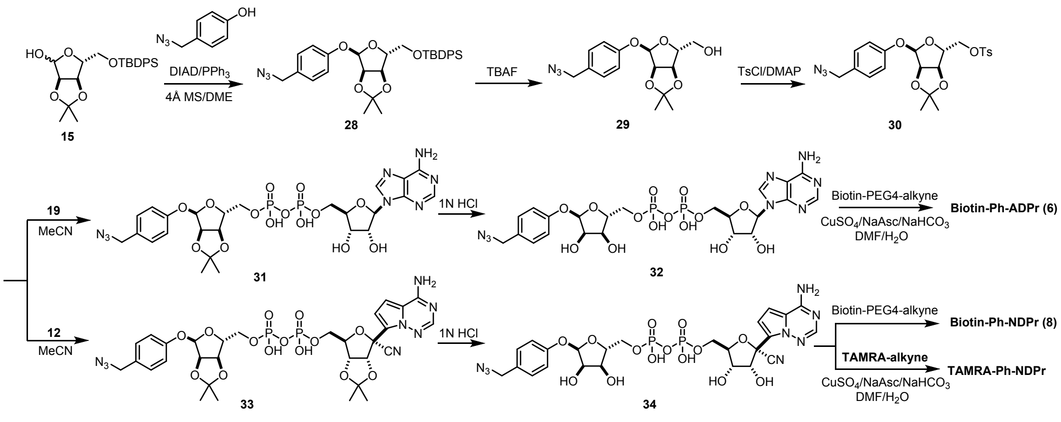

α-1-(4-(azidomethyl)phenoxy)-1-deoxy-2,3-O-isopropylidene-5-OTBDPS-D-ribose (28)

To a flask was added **15** (0.57 g, 1.34 mmol, 1 eq.), 4-(azidomethyl)phenol (0.20 g, 1.34 mmol, 1 eq.), PPh_3 (0.53 g, 2.01 mmol, 1.5 eq.), 4Å molecular sieve (400 mg) and DME (6 mL). The resulting suspension was stirred for 10 minutes, followed by the slow addition of DIAD (396 μL, 2.01 mmol, 1.5 eq.). The reaction was complete after 2 hours with the α-isomer as the major product. Hexanes (15 mL) were added and the mixture was stirred for an additional 10 minutes. The precipitates were filtered off and the filtrate was evaporated to dryness. Column chromatography on silica gel (hexanes:ethyl acetate 8:1) afforded the product **28** as a light-

orange oil (0.46 g, yield: 61%).

^{1}H NMR (500 MHz, $CDCl_3$) δ 7.73 – 7.67 (m, 4H), 7.50 – 7.45 (m, 2H), 7.45 – 7.40 (m, 4H), 7.28 – 7.24 (m, 2H), 7.15 – 7.08 (m, 2H), 5.74 (d, J = 4.4 Hz, 1H), 4.93 (dd, J = 6.4, 4.4 Hz, 1H), 4.86 (dd, J = 6.4, 1.8 Hz, 1H), 4.39 (q, J = 2.5 Hz, 1H), 4.31 (s, 2H), 3.88 (dd, J = 11.3, 3.1 Hz, 1H), 3.81 (dd, J = 11.3, 2.7 Hz, 1H), 1.69 (s, 3H), 1.45 (d, J = 4.8 Hz, 3H), 1.09 (s, 9H).

^{13}C NMR (126 MHz, $CDCl_3$) δ 157.1, 135.6, 135.6, 134.8, 133.0, 132.9, 130.0, 129.9, 129.6, 128.8, 127.9, 127.8, 127.7, 117.0, 114.6, 100.4, 82.7, 81.0, 80.9, 77.3, 77.0, 76.8, 64.8, 54.4, 28.3, 26.9, 26.6, 26.0, 25.9, 19.2.

α-1-(4-(azidomethyl)phenoxy)-1-deoxy-2,3-O-isopropylidene-D-ribose (29)

28 (1.65 g, 2.95 mmol, 1 eq.) was dissolved in 6 mL THF and 1 M TBAF in THF (3.25 mL, 3.25 mmol, 1.1 eq.) was added. The reaction mixture was stirred at room temperature for 2 hours. Purification by column chromatography (hexanes:ethyl acetate 2:1) afforded the product **29** as a colorless oil (0.58 g, yield: 61%).

^{1}H NMR (500 MHz, $CDCl_3$) δ 7.26 – 7.22 (m, 2H), 7.12 – 7.04 (m, 2H), 5.67 (d, J = 4.3 Hz, 1H), 4.84 (dd, J = 7.0, 4.3 Hz, 1H), 4.80 (dd, J = 6.9, 2.7 Hz, 1H), 4.37 (q, J = 3.0 Hz, 1H), 4.28 (s, 2H), 3.91 – 3.85 (m, 1H), 3.80 – 3.74 (m, 1H), 1.68 (s, 3H), 1.42 (s, 3H).

^{13}C NMR (126 MHz, $CDCl_3$) δ 156.9, 129.6, 129.0, 117.1, 115.8, 99.8, 82.7, 81.1, 80.4, 62.8, 54.4, 26.0, 25.9.

α-1-(4-(azidomethyl)phenoxy)-1-deoxy-2,3-O-isopropylidene-5-OTs-D-ribose (30)

General Procedure A was followed. **29** (0.58 g, 1.81 mmol) was reacted to give **30** as a white syrup (0.80 g, yield: 93%).

^{1}H NMR (500 MHz, $CDCl_3$) δ 7.80 (d, J = 8.0 Hz, 2H), 7.37 (d, J = 7.9 Hz, 2H), 7.22 (d, J = 8.5 Hz, 2H), 6.99 (m, d, J = 8.4 Hz, 2H), 5.49 (d, J = 4.4 Hz, 1H), 4.80 (dd, J = 6.7, 4.5 Hz, 1H), 4.70 (td, J = 6.4, 2.4 Hz, 1H), 4.40 (q, J = 2.9 Hz, 1H), 4.27 (s, 2H), 4.23 – 4.19 (m, 2H), 2.46 (s, 3H), 1.64 (s, 3H), 1.38 (s, 3H).

^{13}C NMR (126 MHz, $CDCl_3$) δ 156.8, 145.3, 132.6, 130.1, 130.0, 129.6, 129.1, 128.0, 127.9, 117.0, 115.7, 99.8, 80.6, 80.2, 79.6, 77.3, 77.0, 76.8, 69.6, 54.3, 25.9, 25.9, 21.7.

α-1''-(4-(azidomethyl)phenoxy)-1''-deoxy-2',3':2'',3''-bis-O-isopropylidene-ADP-ribose (31)

General Procedure B was followed. **30** (320 mg, 0.673 mmol) was reacted with **19** to yield **31** as a white solid (12 mg, yield: 2.4%)

LC-MS (ESI): m/z calcd for $C_{25}H_{31}N_8O_{14}P_2^-$ [M-H]$^-$ 729.1, found 729.3.

^{1}H NMR (500 MHz, D_2O) δ 8.42 (s, 1H), 8.12 (s, 1H), 7.13 (d, J = 8.3 Hz, 2H), 6.87 (d, J = 8.2 Hz, 2H), 6.00 (d, J = 5.3 Hz, 1H), 5.68 (d, J = 4.2 Hz, 1H), 5.03 (dd, J = 6.2, 4.2 Hz, 1H), 4.95 (d, J = 6.3 Hz, 1H), 4.58 (t, J = 5.2 Hz, 1H), 4.47 – 4.40 (m, 2H), 4.34 (d, J = 3.7 Hz, 1H), 4.22 (d, J = 17.6 Hz, 4H), 4.08 (dd, J = 5.6, 2.8 Hz, 2H), 1.52 (s, 3H), 1.37 (d, J = 5.7 Hz, 3H).

^{13}C NMR (126 MHz, D_2O) δ 155.6, 152.6, 142.5, 139.5, 129.8, 116.2, 114.5, 106.7, 100.4, 87.2, 80.7, 79.6, 74.6, 70.1, 66.6, 65.2, 53.6, 24.8, 24.3.

^{31}P NMR (202 MHz, D_2O) δ -11.26

α-1''-(4-(azidomethyl)phenoxy)-1''-deoxy-ADP-ribose (32)

General Procedure C was followed. **31** (7.0 mg, 9.6 μmol) was stirred in 200 μL 1N HCl to give **32** as a white solid (5.8 mg, yield: 88%)

LC-MS (ESI): m/z calcd for $C_{22}H_{27}N_8O_{14}P_2^-$ [M-H]$^-$ 689.1, found 689.3.

^{1}H NMR (500 MHz, D_2O) δ 8.61 (s, 1H), 8.36 (s, 1H), 7.24 (d, J = 8.2 Hz, 2H), 6.99 (d, J = 8.5 Hz, 2H), 6.13 (d, J = 5.4 Hz, 1H), 5.63 (d, J = 4.4 Hz, 1H), 4.51 (t, J = 4.4 Hz, 1H), 4.74 (1H, overlapped with the solvent peak) 4.40 – 4.35 (m, 3H), 4.32 (s, 2H), 4.30 – 4.21 (m, 3H), 4.09 (q, J = 5.3, 4.7 Hz, 2H).

^{13}C NMR (126 MHz, D_2O) δ 156.3, 144.9, 142.5, 129.9, 129.8, 117.1, 100.8, 88.0, 84.3, 84.2, 74.7, 71.2, 70.3, 69.7, 53.7.

^{31}P NMR (202 MHz, D_2O) δ -11.05 (d, J = 21.4 Hz), -11.32 (d, J = 21.7 Hz).

Biotin-Ph-ADPr (6)

General Procedure D was followed. **32** (2.0 mg, 2.9 μmol) was reacted with biotin-PEG4-alkyne to give **6** as a white solid (2.8 mg, yield: 84%)

LC-MS (ESI): m/z calcd for $C_{43}H_{64}N_{11}O_{20}P_2S^+$ $[M+H]^+$ 1148.4, found 1148.3.

^{1}H NMR (500 MHz, D_2O) δ 8.65 (s, 1H), 8.37 (s, 1H), 8.04 (s, 1H), 7.26 (d, J = 8.0 Hz, 2H), 7.01 (d, J = 8.1 Hz, 2H), 6.13 (d, J = 5.3 Hz, 1H), 5.65 (d, J = 4.4 Hz, 1H), 5.53 (s, 2H), 4.75 (1H, overlapped with the solvent peak), 4.67 (s, 2H), 4.52 (p, J = 4.5 Hz, 2H), 4.43 – 4.32 (m, 3H), 4.28 (q, J = 9.5, 7.5 Hz, 4H), 4.10 (s, 2H), 3.71 (d, J = 4.3 Hz, 2H), 3.67 (d, J = 5.2 Hz, 2H), 3.63 (s, 8H), 3.57 (t, J = 5.2 Hz, 2H), 3.33 (t, J = 5.2 Hz, 2H), 3.17 (dt, J = 9.6, 5.0 Hz, 1H), 2.87 (td, J = 13.1, 4.9 Hz, 1H), 2.70 (d, J = 13.1 Hz, 1H), 2.17 (t, J = 7.5 Hz, 2H), 1.69 – 1.40 (m, 4H), 1.39 – 1.20 (m, 2H).

^{13}C NMR (126 MHz, D_2O) δ 179.3, 168.3, 167.8, 159.1, 152.3, 147.1, 146.7, 145.0, 132.6, 132.2, 131.3, 127.4, 119.7, 103.1, 90.5, 77.2, 73.6, 72.8, 72.2, 72.1, 72.1, 71.9, 71.5, 71.4, 68.2, 67.6, 65.6, 64.5, 62.7, 57.8, 55.9, 42.2, 41.4, 37.9, 30.4, 30.1, 27.6.

α-1''-(4-(azidomethyl)phenoxy)-1''-deoxy-2',3':2'',3''-bis-O-isopropylidene-NDP-ribose (33)

General Procedure B was followed. **30** (63 mg, 0.134 mmol) was reacted with **12** to give **33** as a white solid (5 mg, yield: 4.7%)

LC-MS (ESI): m/z calcd for $C_{30}H_{35}N_8O_{14}P_2^-$ $[M-H]^-$ 793.2, found 793.3.

^{1}H NMR (500 MHz, D_2O) δ 7.92 (s, 1H), 7.21 (d, J = 8.5 Hz, 1H), 7.11 (d, J = 4.8 Hz, 1H), 6.99 (d, J = 4.8 Hz, 1H), 6.97 (d, J = 8.7 Hz, 1H), 5.76 (d, J = 4.2 Hz, 1H), 5.23 (d, J = 6.4 Hz, 1H), 5.17 (dd, J = 6.5, 2.4 Hz, 1H), 5.03 (dd, J = 6.2, 4.3 Hz, 1H), 4.98 (dd, J = 6.2, 1.4 Hz, 1H), 4.74 (1H, overlapped with the solvent peak), 4.40 (d, J = 3.2 Hz, 1H), 4.28 (s, 2H), 4.14 (q, J = 5.4 Hz, 2H), 4.05 (dt, J = 6.0, 2.9 Hz, 2H), 1.73 (s, 3H), 1.57 (s, 3H), 1.45 (s, 3H), 1.40 (s, 3H).

^{13}C NMR (126 MHz, D_2O) δ 155.8, 150.3, 138.7, 129.9, 129.7, 126.8, 117.1, 116.5, 115.6, 114.5, 114.3, 112.7, 108.0, 100.4, 85.1, 84.3, 84.2, 81.7, 80.8, 80.7, 80.7, 79.6, 66.6, 65.1, 53.6, 25.1, 24.9, 24.3, 24.3.

^{31}P NMR (202 MHz, D_2O) δ -11.32 (d, J = 21.0 Hz), -11.70 (d, J = 21.2 Hz).

α-1''-(4-(azidomethyl)phenoxy)-1''-deoxy-NDP-ribose (34)

General Procedure C was followed. **33** (5.0 mg, 6.3 μmol) was stirred in 200 μL 1N HCl to give **34** as a white solid (4.0 mg, yield: 89%)

LC-MS (ESI): m/z calcd for $C_{60}H_{67}N_{12}O_{19}P_2^-$ [M-H]$^-$ 713.1, found 713.2.

^{1}H NMR (500 MHz, D_2O) δ 7.84 (s, 1H), 7.16 (d, J = 8.7 Hz, 1H), 6.98 (d, J = 4.7 Hz, 1H), 6.89 (d, J = 8.6 Hz, 1H), 6.85 (d, J = 4.7 Hz, 1H), 5.45 (d, J = 4.5 Hz, 1H), 4.85 (d, J = 5.3 Hz, 1H), 4.48 (q, J = 4.1 Hz, 1H), 4.40 (t, J = 5.0 Hz, 1H), 4.33 – 4.28 (m, 3H), 4.27 – 4.21 (m, 2H), 4.20 – 4.10 (m, 2H), 3.99 (t, J = 4.3 Hz, 2H).

^{13}C NMR (126 MHz, D_2O) δ 156.2, 154.3, 145.4, 140.2, 129.8, 129.6, 123.7, 116.9, 116.7, 116.0, 111.4, 103.5, 100.6, 92.5, 84.2, 84.1, 84.0, 77.4, 74.9, 71.1, 70.1, 69.7, 65.5, 65.5, 64.6, 53.7.

^{31}P NMR (202 MHz, D_2O) δ -11.10 (d, J = 21.8 Hz), -11.41 (d, J = 21.7 Hz).

Biotin-Ph-NDPr (8)

General Procedure D was followed. **34** (2.0 mg, 2.8 μmol) was reacted with biotin-PEG4-alkyne to yield **8** as a white solid (2.6 mg, yield: 79%).

LC-MS (ESI): m/z calcd for $C_{45}H_{64}N_{11}O_{20}P_2S^+$ [M+H]$^+$ 1172.4, found 1172.4.

^{1}H NMR (500 MHz, D_2O) δ 8.02 (s, 1H), 7.98 (s, 1H), 7.26 – 7.19 (m, 3H), 7.10 (d, J = 4.9 Hz, 1H), 6.96 (d, J = 8.3 Hz, 2H), 5.57 – 5.54 (m, 1H), 5.50 (s, 2H), 4.86 (d, J = 5.2 Hz, 1H), 4.65 (s, 2H), 4.48 (d, J = 6.8 Hz, 2H), 4.40 (t, J = 4.5 Hz, 1H), 4.34 (t, J = 5.4 Hz, 1H), 4.29 – 4.23 (m, 3H), 4.17 (s, 2H), 4.04 (s, 2H), 3.69 (d, J = 6.7 Hz, 2H), 3.65 (d, J = 5.3 Hz, 2H), 3.61 (s, 8H), 3.55 (t, J = 5.4 Hz, 2H), 3.33 – 3.29 (m, 2H), 3.14 (dt, J = 9.7, 5.1 Hz, 1H), 2.85 (ddt, J = 12.3, 8.6, 3.2 Hz, 2H), 2.67 (d, J = 12.9 Hz, 1H), 2.14 (t, J = 7.1 Hz, 2H), 1.64 – 1.45 (m, 4H), 1.29 – 1.24 (m, 2H).

^{13}C NMR (126 MHz, D_2O) δ 179.3, 168.3, 167.8, 159.1, 152.3, 147.1, 146.7, 145.0, 132.6, 132.2, 131.3, 127.4, 119.7, 103.1, 90.5, 77.2, 73.6, 72.8, 72.2, 72.1, 72.1, 71.9, 71.5, 71.4, 68.2, 67.6, 65.6, 64.5, 62.7, 57.8, 55.9, 42.2, 41.4, 37.9, 30.4, 30.1, 27.6.

TAMRA-Ph-NDPr

General Procedure D was followed. **34** (2.0 mg, 2.8 μmol) was reacted with biotin-PEG4-

alkyne to yield **TAMRA-Ph-NDPr** as a dark purple solid (2.2 mg, yield: 59%).

LC-MS (ESI): m/z calcd for $C_{60}H_{67}N_{12}O_{19}P_2^-$ [M-H]$^-$ 1321.4, found 1321.5.

^{1}H NMR (500 MHz, D_2O) δ 8.30 (s, 1H), 8.01 (d, J = 7.7 Hz, 1H), 7.66 (s, 1H), 7.63 (s, 1H) 7.24 (d, J = 7.8 Hz, 1H), 7.05 (d, J = 8.3 Hz, 2H), 6.89 (d, J = 9.2 Hz, 2H), 6.80 (d, J = 8.1 Hz, 2H), 6.71 (d, J = 4.5 Hz, 1H), 6.66 – 6.59 (m, 2H), 6.55 (s, 1H), 6.31 (d, J = 8.5 Hz, 2H), 5.32 (s, 3H), 4.62 (1H, overlapped with the solvent peak), 4.45 – 4.40 (m, 1H), 4.36 (s, 1H), 4.20 (s, 2H), 4.11 (s, 3H), 4.02 – 3.82 (m, 2H), 3.45 (s, 2H), 3.08 – 3.03 (m, 14H), 2.89 (t, J = 7.1 Hz, 2H), 2.48 (t, J = 7.1 Hz, 1H), 1.59 (s, 2H), 1.32 – 1.21 (m, 4H), 1.08 (s, 2H).

^{13}C NMR (126 MHz, D_2O) δ 174.5, 169.0, 163.2, 162.9, 158.1, 156.6, 146.4, 135.3, 134.3, 130.6, 129.5, 128.6, 128.3, 123.3, 123.2, 117.5, 117.0, 115.2, 113.7, 112.6, 110.9, 100.4, 95.7, 84.2, 79.9, 77.3, 75.0, 71.1, 70.1, 69.5, 65.4, 64.7, 53.2, 40.0, 35.4, 28.3, 28.1, 27.4, 25.8, 25.5.

^{31}P NMR (202 MHz, D_2O) δ -11.16 (d, J = 20.8 Hz), -11.38 (d, J = 20.6 Hz).

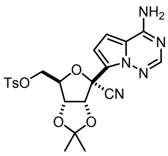
NH2
N
N
N
O
TsO
CN
O
O

10

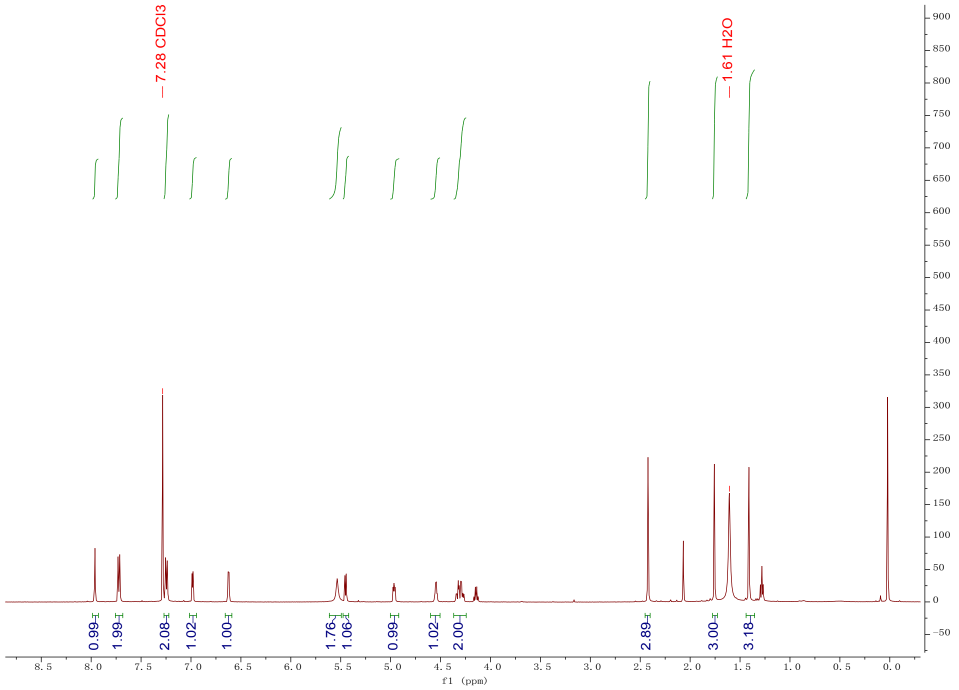
7.28 CDCl3
1.61 H2O
0.99
1.99
2.08
1.02
1.00
1.76
1.06
0.99
1.02
2.00
2.89
3.00
3.18
f1 (ppm)

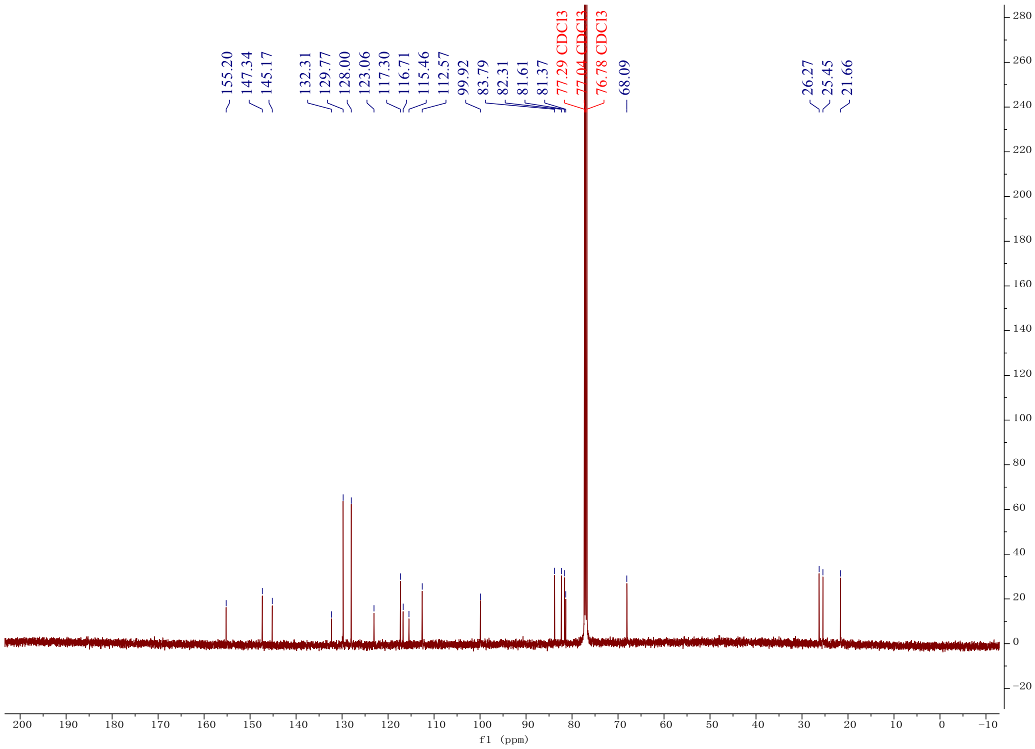
155.20
147.34
145.17
132.31
129.77
128.00
123.06
117.30
116.71
115.46
112.57
99.92
83.79
82.31
81.61
81.37
77.29 CDCl3
77.04 CDCl3
76.78 CDCl3
68.09
26.27
25.45
21.66
f1 (ppm)

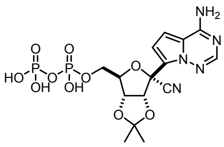
NH2
HO
OH
OH
CN

11

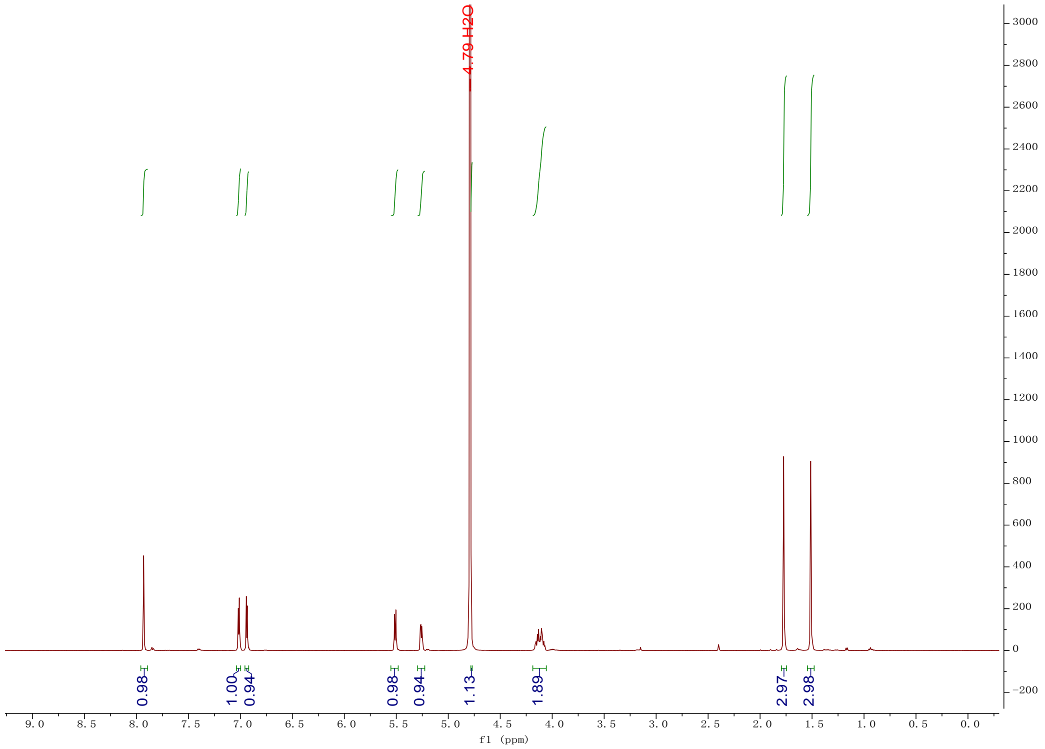
4.79 H2O
0.98
1.00
0.94
0.98
0.94
1.13
1.89
2.97
2.98
f1 (ppm)

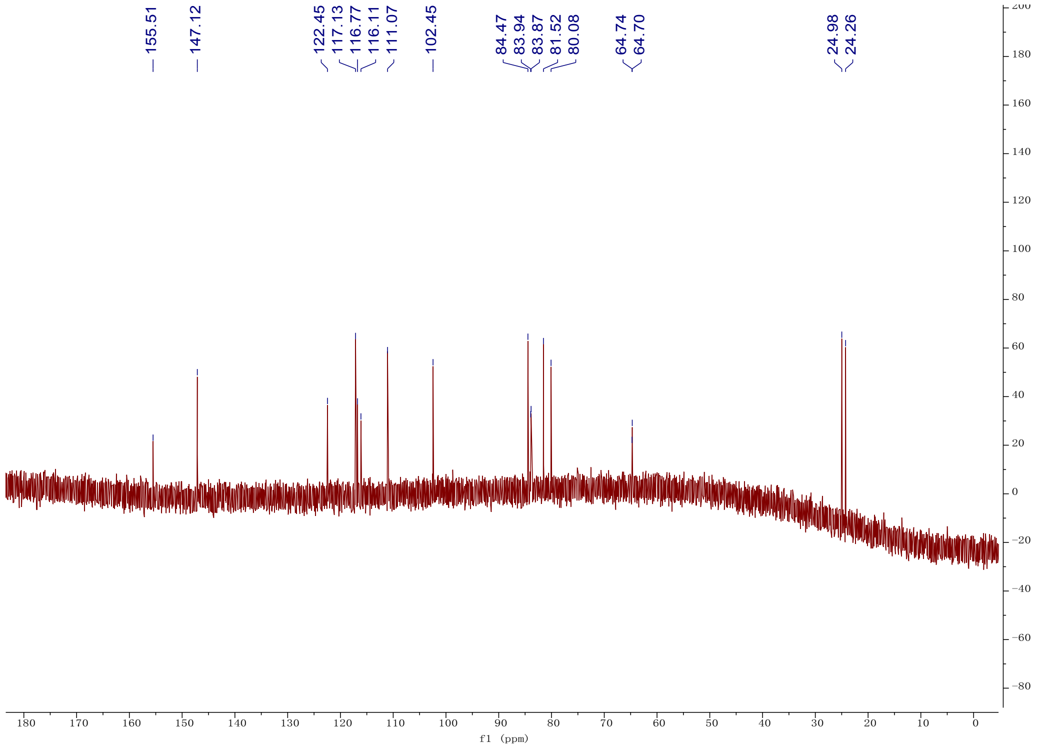
155.51
147.12
122.45
117.13
116.77
116.11
111.07
102.45
84.47
83.94
83.87
81.52
80.08
64.74
64.70
24.98
24.26
f1 (ppm)

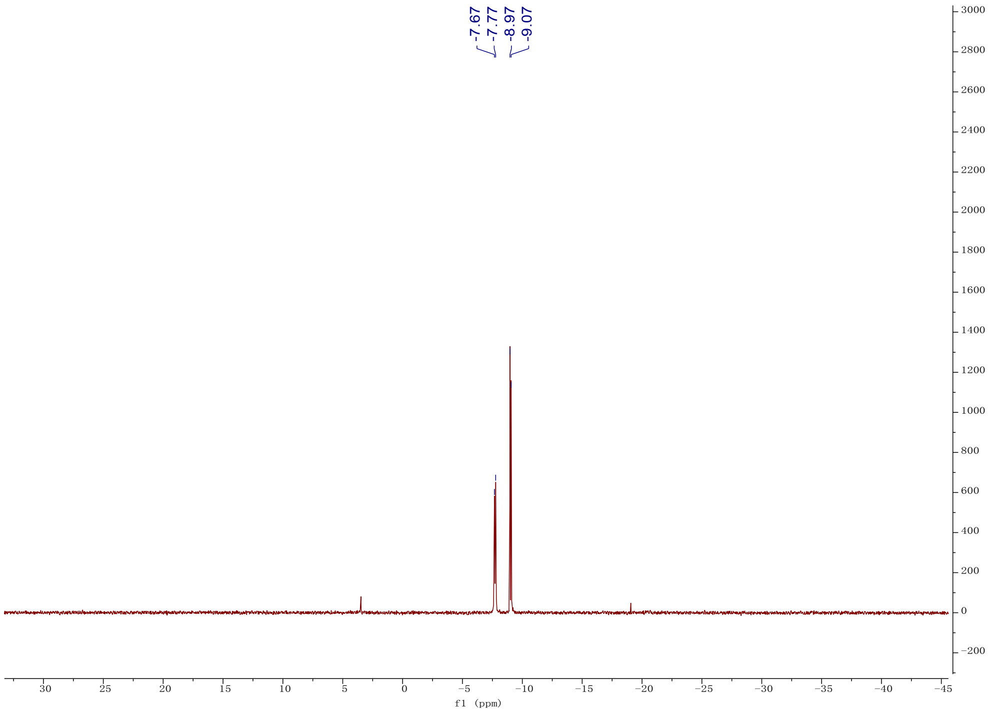
-7.67
-7.77
-8.97
-9.07
f1 (ppm)

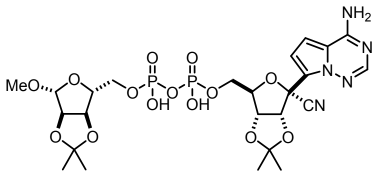
NH2
N
N
N
MeO
O
P
OH
OH
CN

14

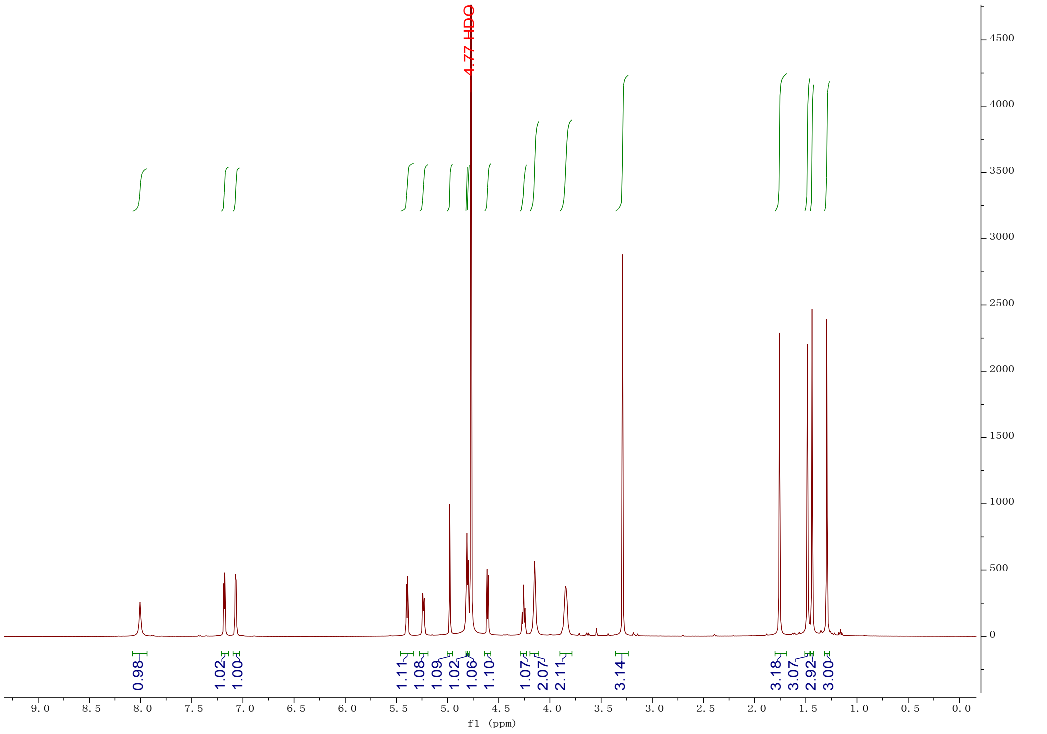
4.77 HDO
0.98
1.02
1.00
1.11
1.08
1.09
1.02
1.06
1.10
1.07
2.07
2.11
3.14
3.18
3.07
2.92
3.00
9.0 8.5 8.0 7.5 7.0 6.5 6.0 5.5 5.0 4.5 4.0 3.5 3.0 2.5 2.0 1.5 1.0 0.5 0.0
f1 (ppm)
4500 4000 3500 3000 2500 2000 1500 1000 500 0

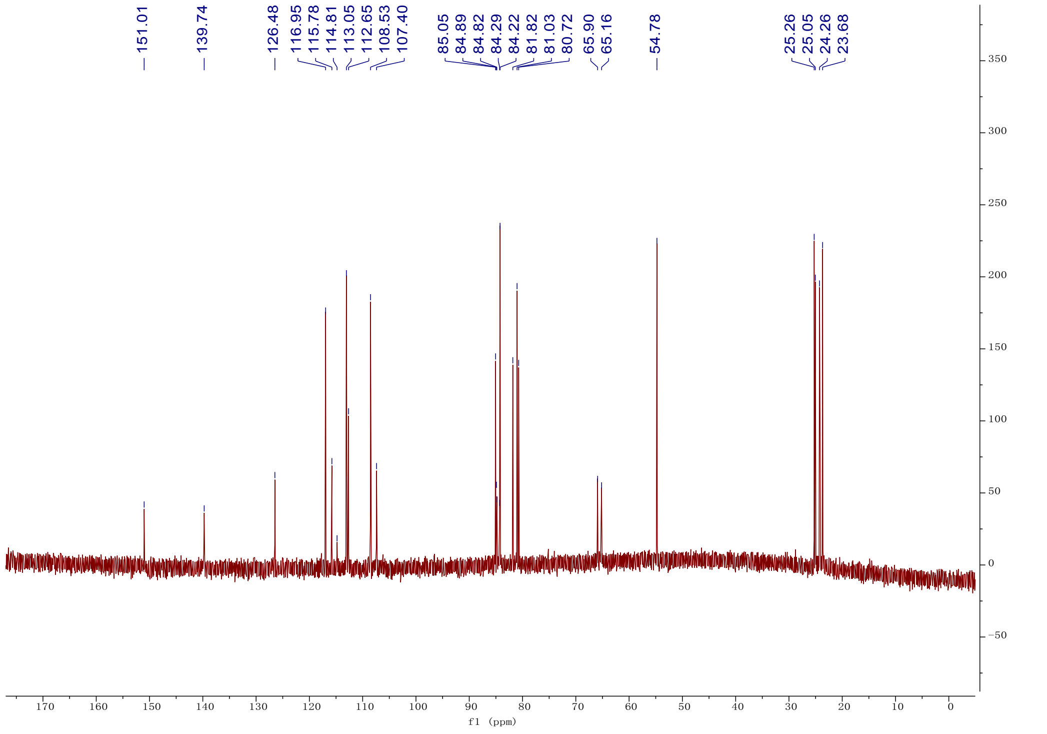
151.01
139.74
126.48
116.95
115.78
114.81
113.05
112.65
108.53
107.40
85.05
84.89
84.82
84.29
84.22
81.82
81.03
80.72
65.90
65.16
54.78
25.26
25.05
24.26
23.68
170 160 150 140 130 120 110 100 90 80 70 60 50 40 30 20 10 0
f1 (ppm)
350 300 250 200 150 100 50 0 -50

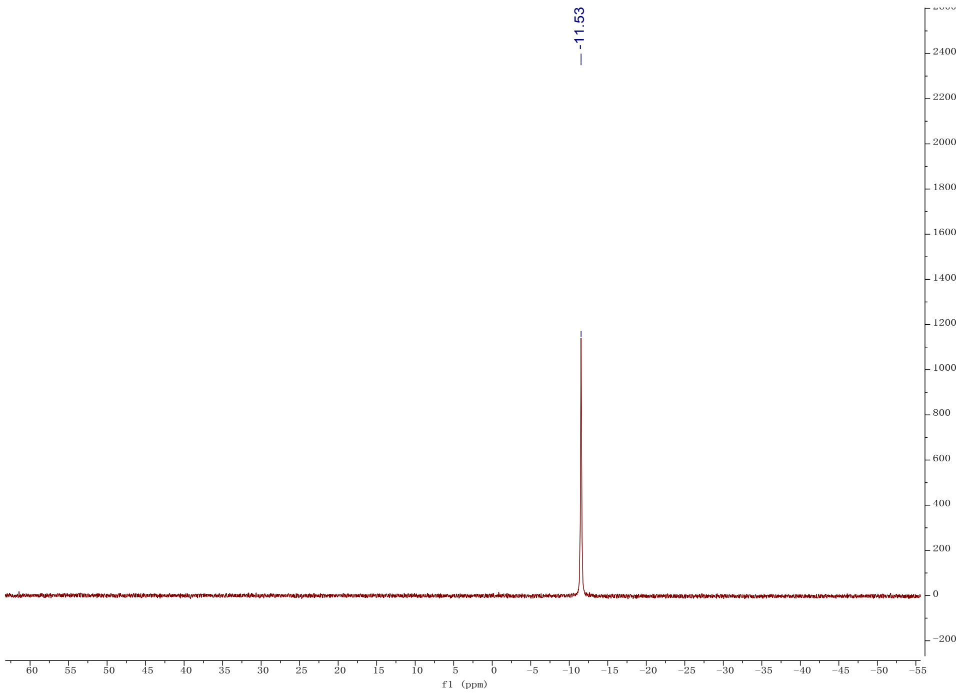
-11.53
f1 (ppm)

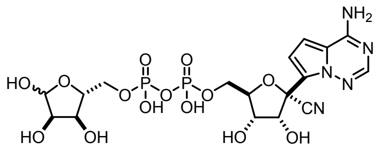
NH2
N
N
N
O
CN
HO
OH
O
O
P
P
O
O
O
OH
OH
O
HO
HO
OH

NDPr
3

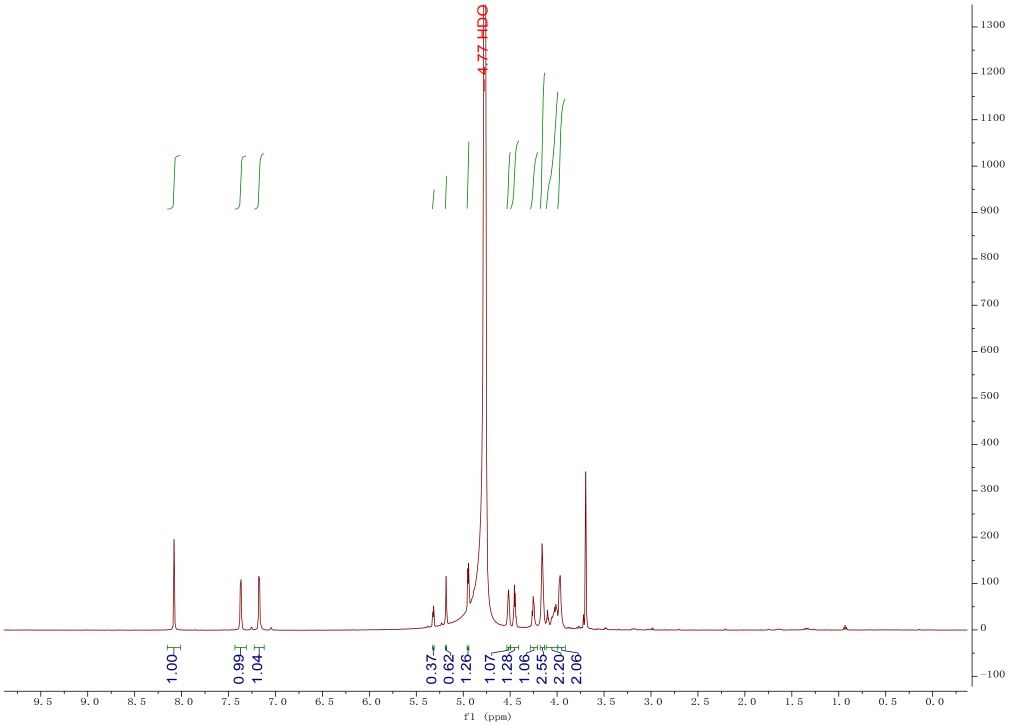
4.77 HDO
1.00
0.99
1.04
0.37
0.62
1.26
1.07
1.28
1.06
2.55
2.20
2.06
f1 (ppm)

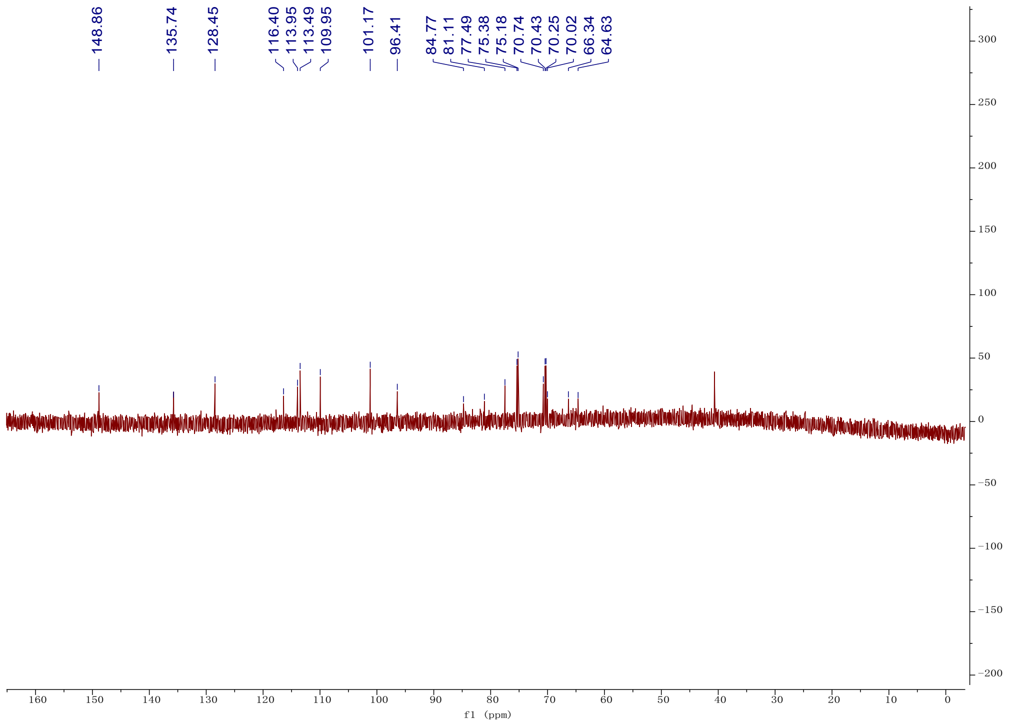
148.86
135.74
128.45
116.40
113.95
113.49
109.95
101.17
96.41
84.77
81.11
77.49
75.38
75.18
70.74
70.43
70.25
70.02
66.34
64.63
f1 (ppm)

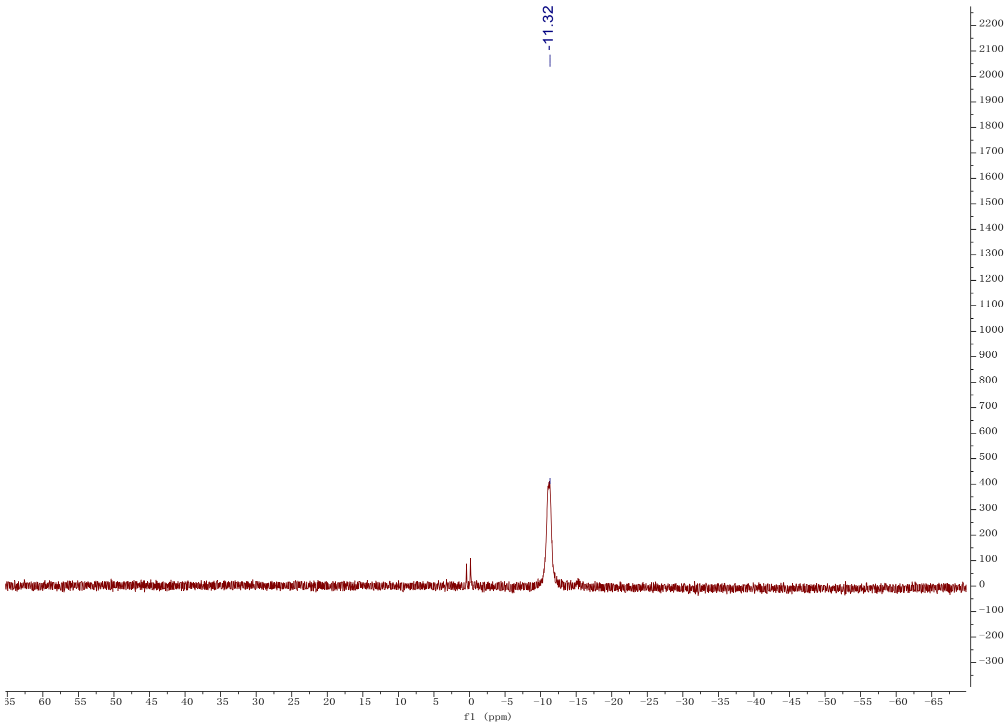
-11.32
f1 (ppm)

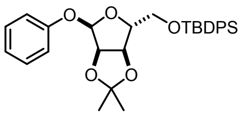

16

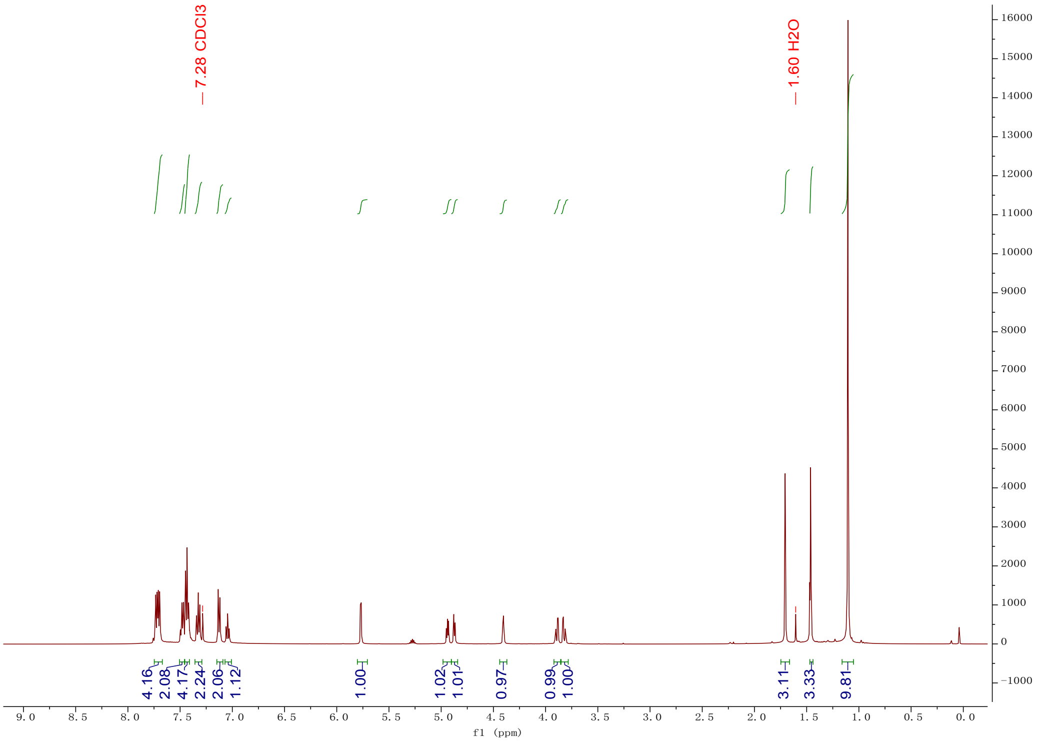

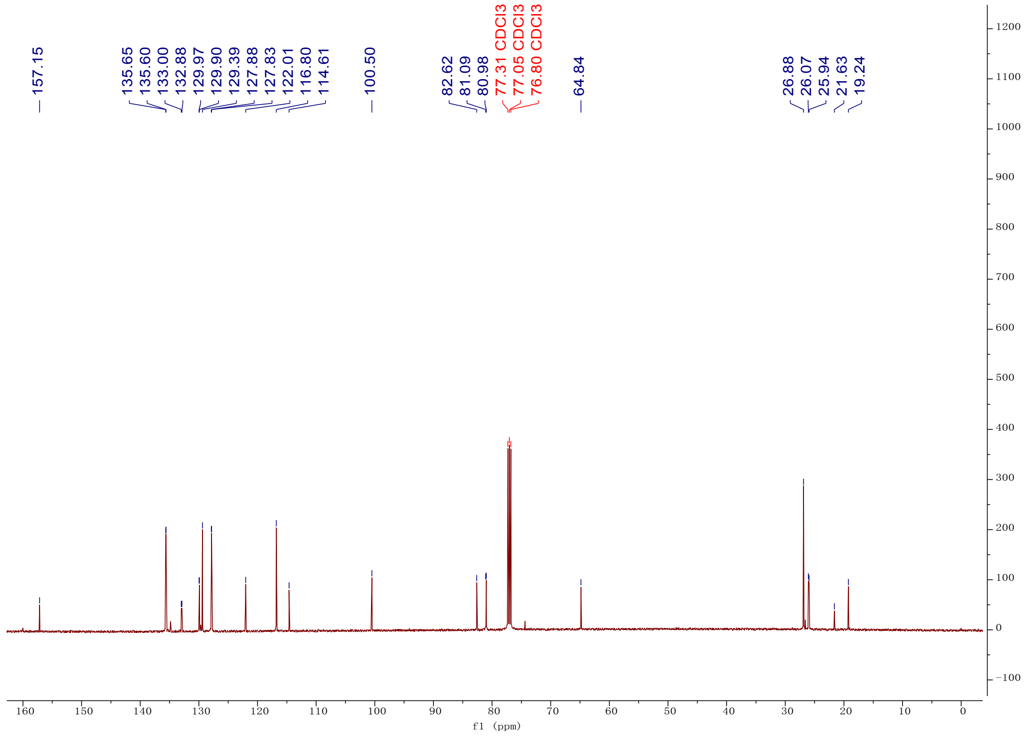

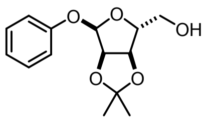

17

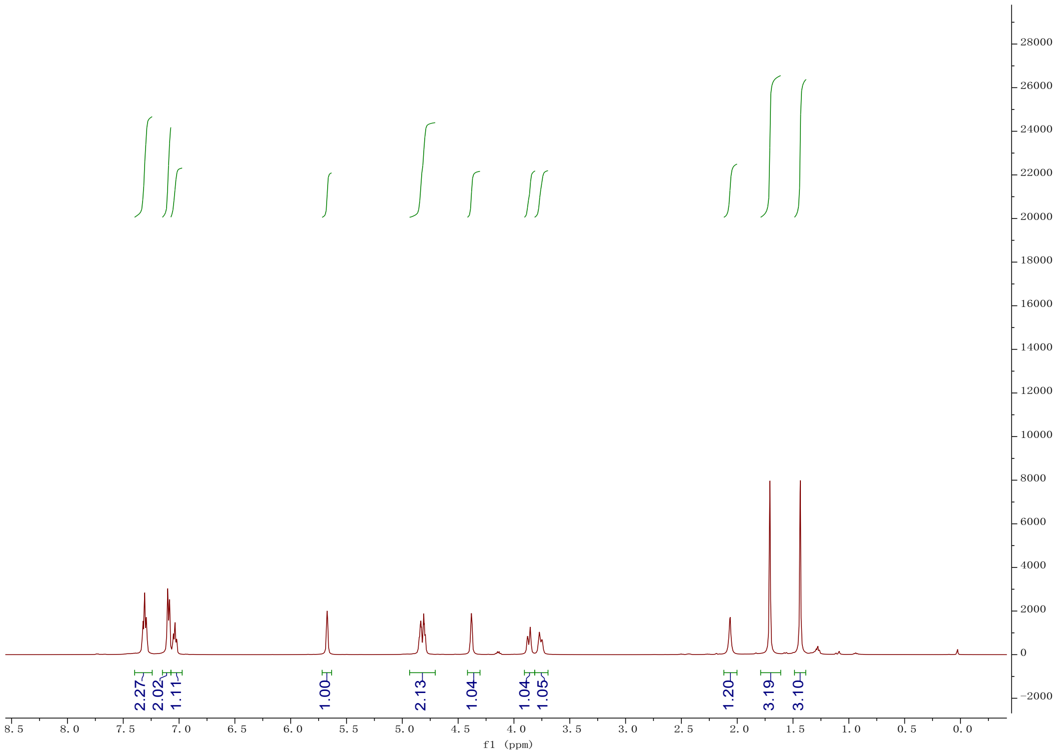

2.27
2.02
1.11
1.00
2.13
1.04
1.04
1.05
1.20
3.19
3.10
f1 (ppm)

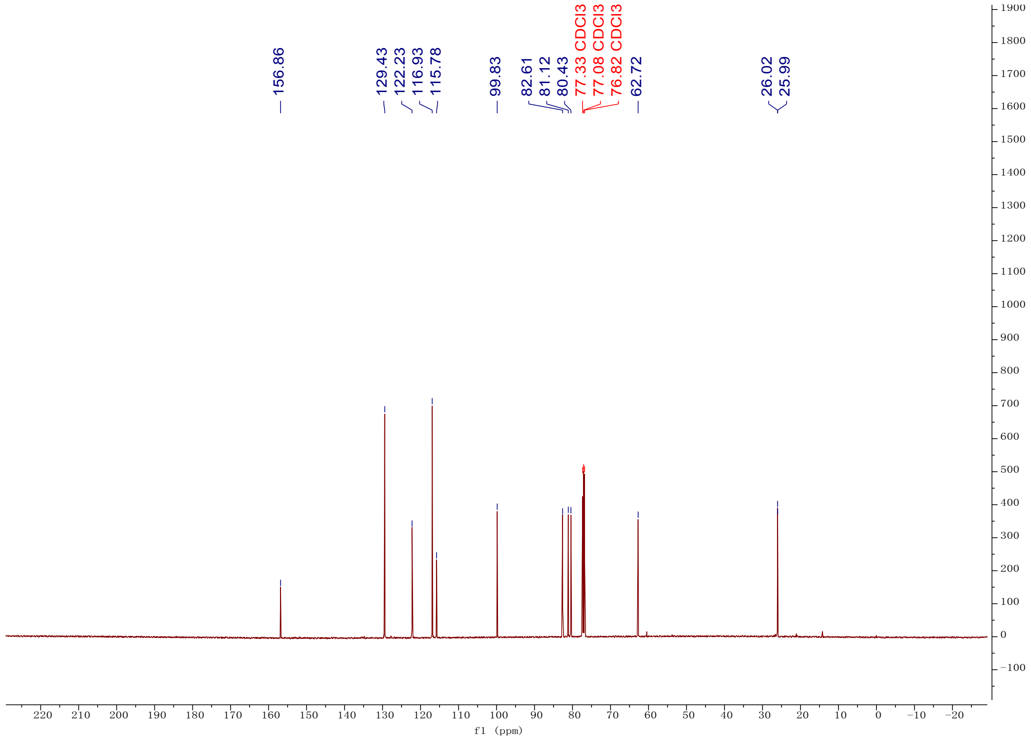

156.86
129.43
122.23
116.93
115.78
99.83
82.61
81.12
80.43
77.33 CDCl3
77.08 CDCl3
76.82 CDCl3
62.72
26.02
25.99
f1 (ppm)

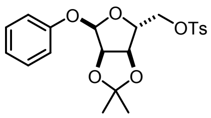

18

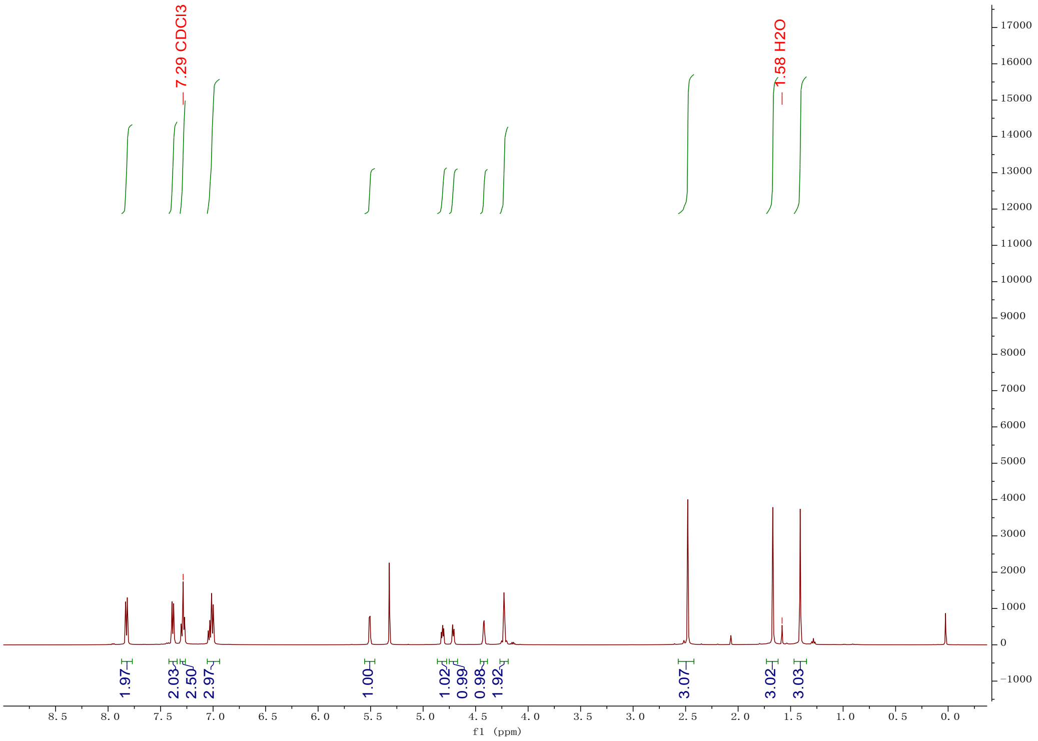

7.29 CDCl3
1.58 H2O
1.97
2.03
2.50
2.97
1.00
1.02
0.99
0.98
1.92
3.07
3.02
3.03
f1 (ppm)

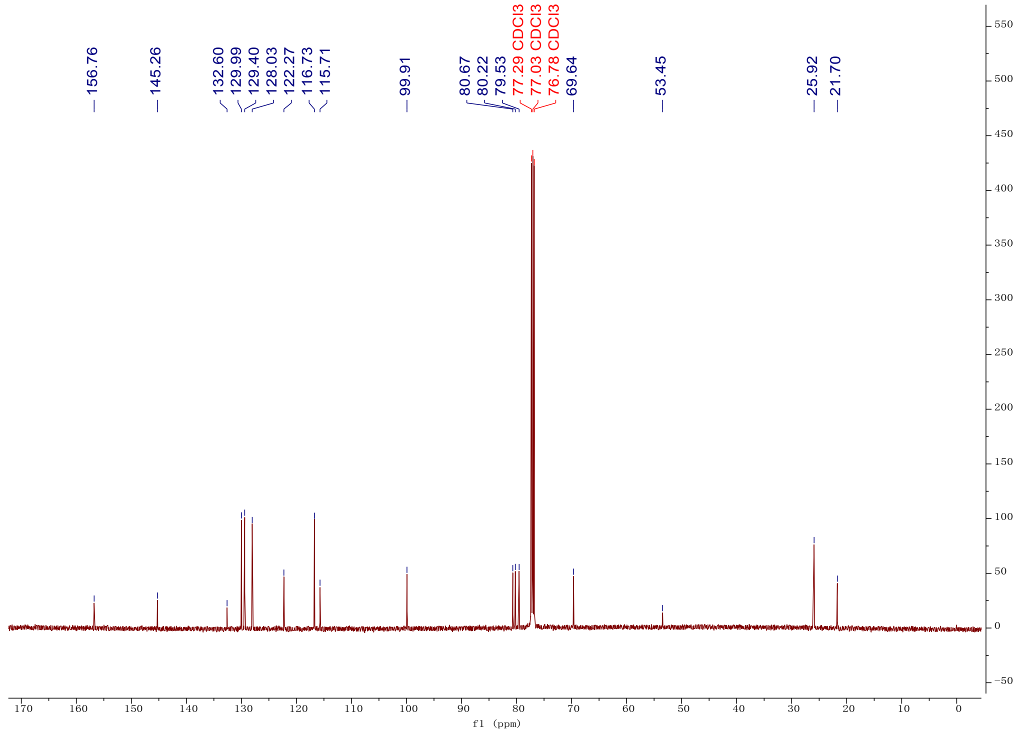

156.76
145.26
132.60
129.99
129.40
128.03
122.27
116.73
115.71
99.91
80.67
80.22
79.53
77.29 CDCl3
77.03 CDCl3
76.78 CDCl3
69.64
53.45
25.92
21.70
f1 (ppm)

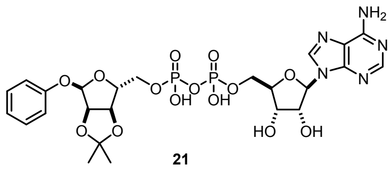
NH2
N
N
N
N
O
O
O
O
O
P
P
OH
OH
O
O
O
O
O
HO
OH

21

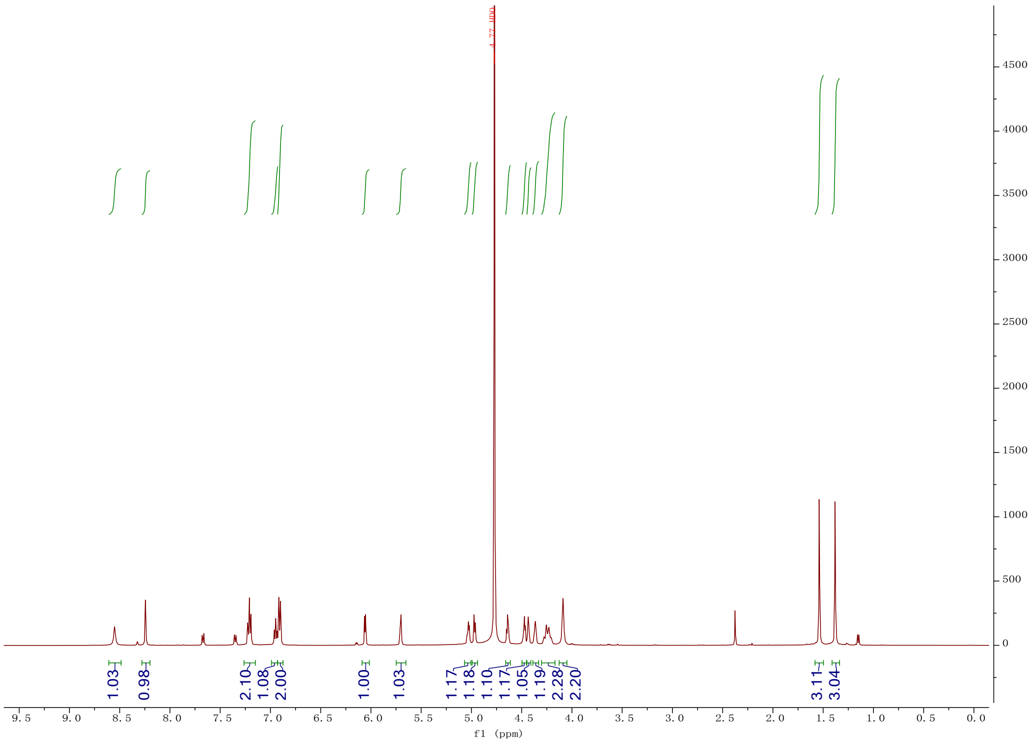
4.77 HDO
1.03
0.98
2.10
1.08
2.00
1.00
1.03
1.17
1.18
1.10
1.17
1.05
1.19
2.28
2.20
3.11
3.04
f1 (ppm)

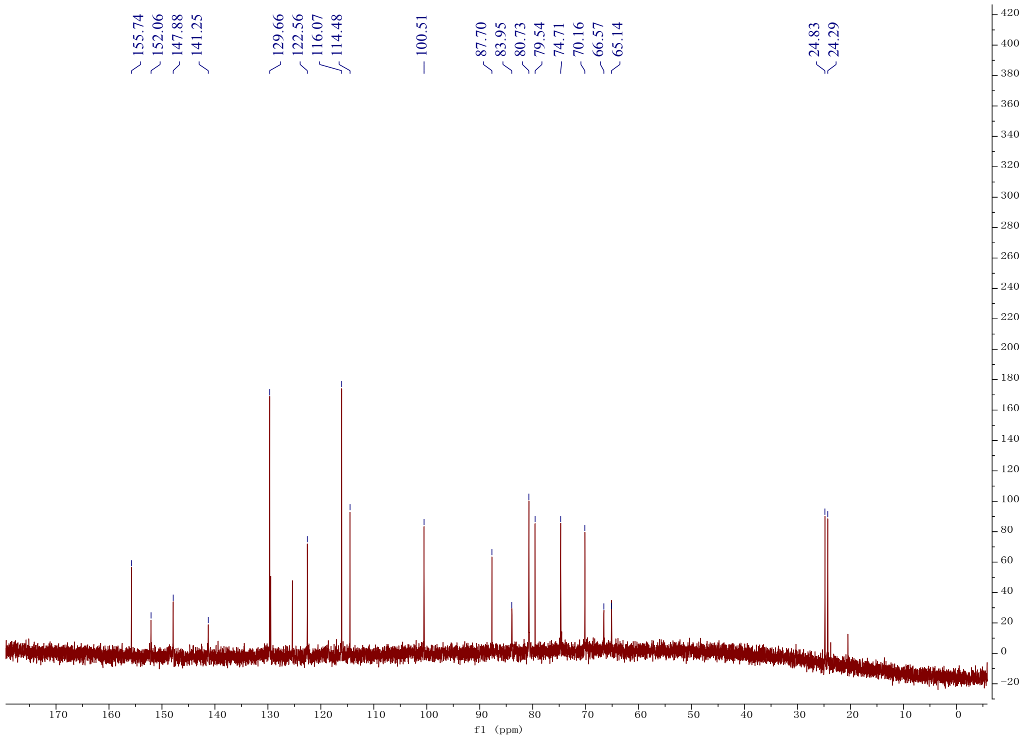
155.74
152.06
147.88
141.25
129.66
122.56
116.07
114.48
100.51
87.70
83.95
80.73
79.54
74.71
70.16
66.57
65.14
24.83
24.29
f1 (ppm)

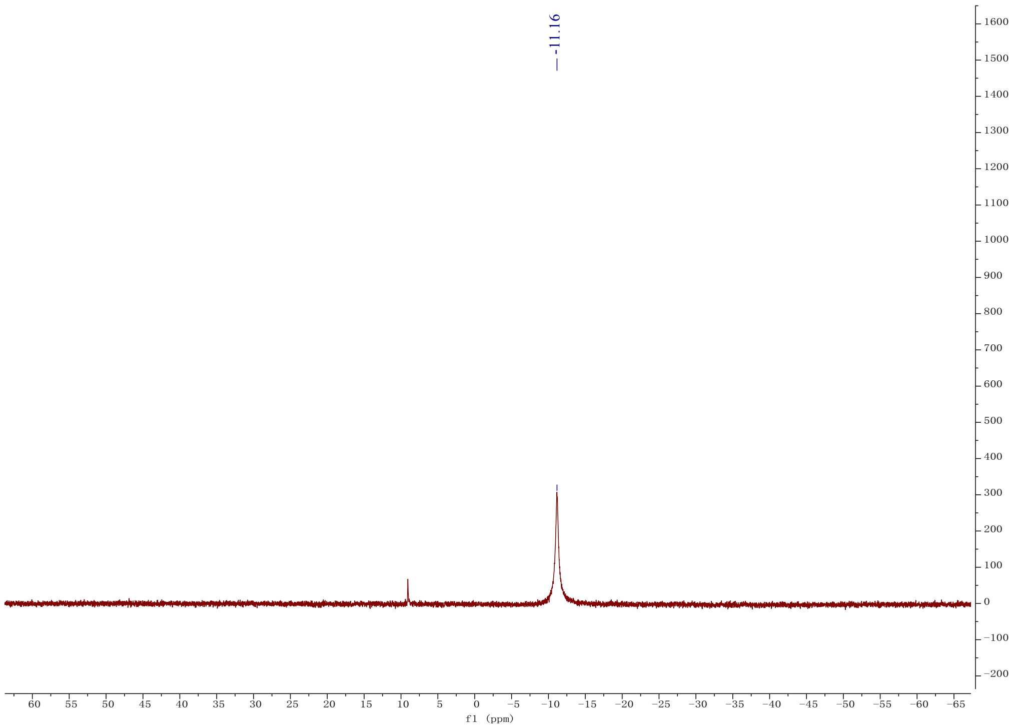
-11.16
f1 (ppm)

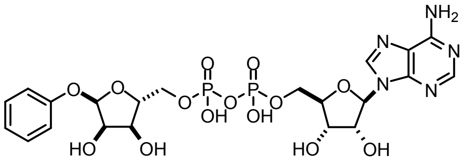

Ph-ADPr
2

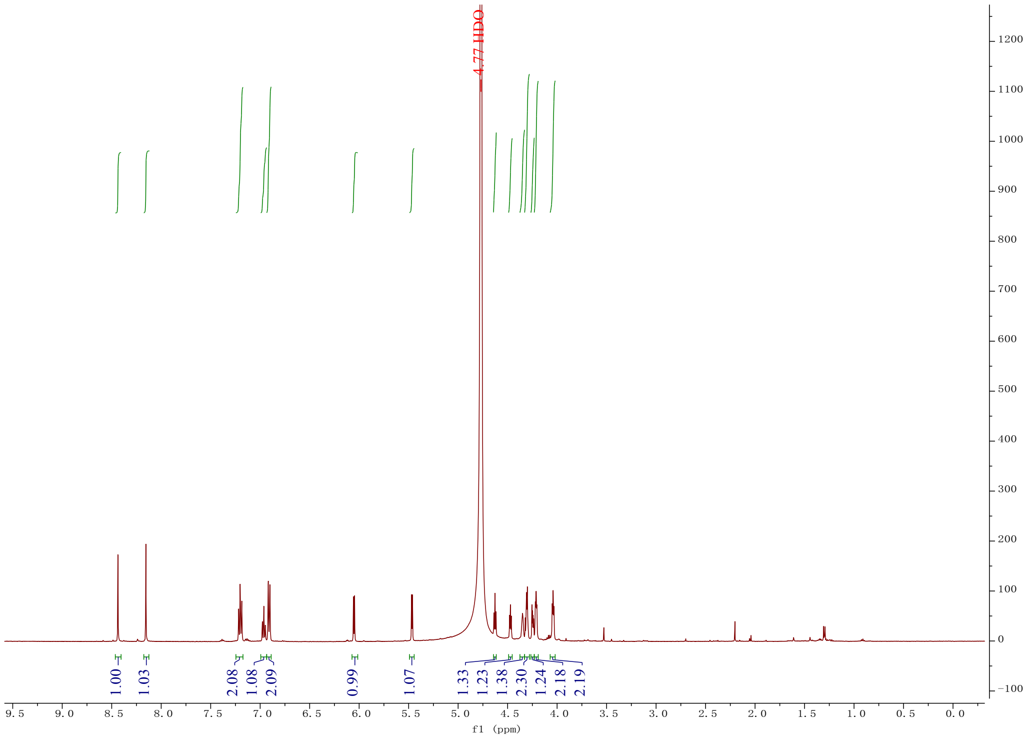

4.77 HDO
1.00
1.03
2.08
1.08
2.09
0.99
1.07
1.33
1.23
1.38
2.30
1.24
2.18
2.19
f1 (ppm)

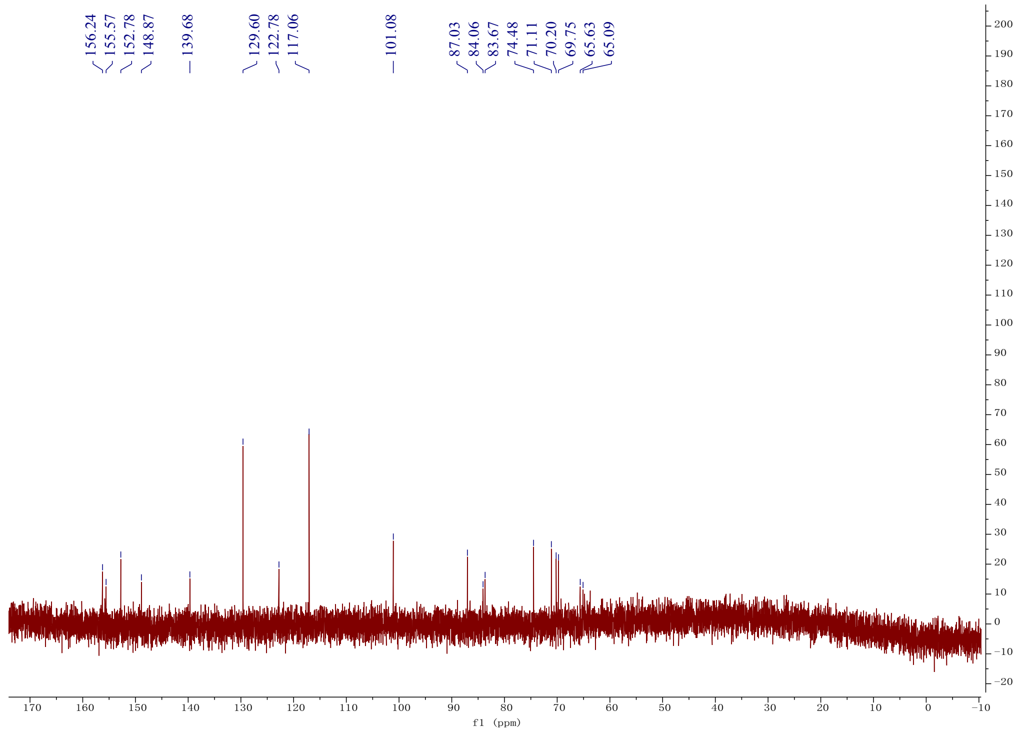

156.24
155.57
152.78
148.87
139.68
129.60
122.78
117.06
101.08
87.03
84.06
83.67
74.48
71.11
70.20
69.75
65.63
65.09
f1 (ppm)

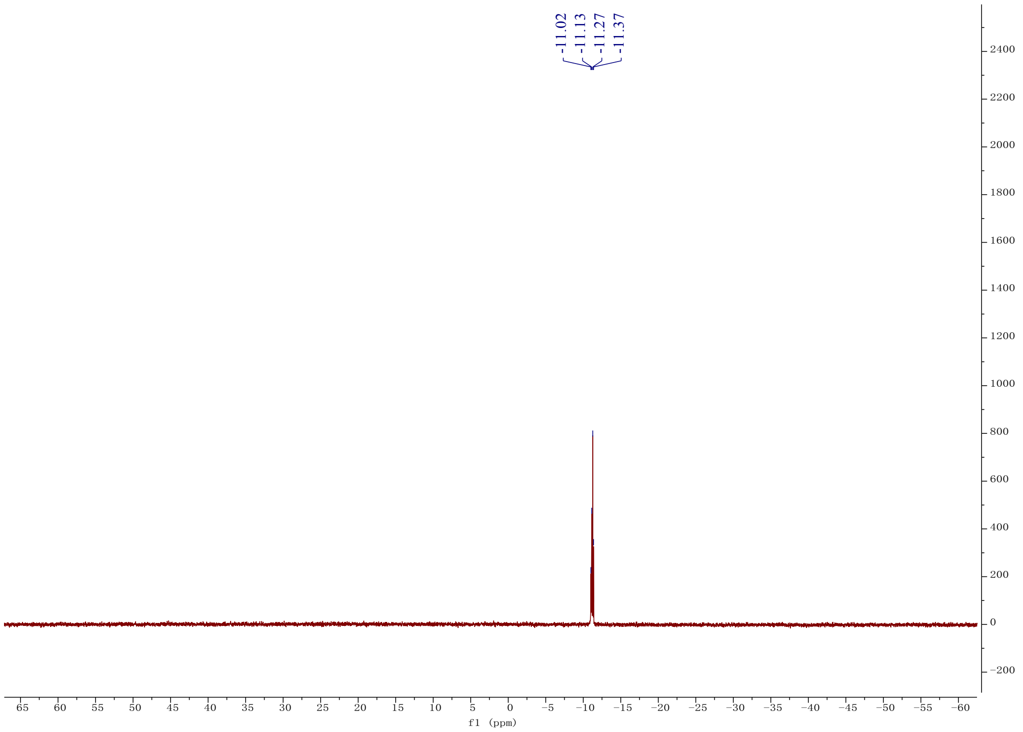
-11.02
-11.13
-11.27
-11.37
f1 (ppm)

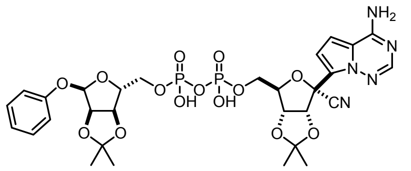

20

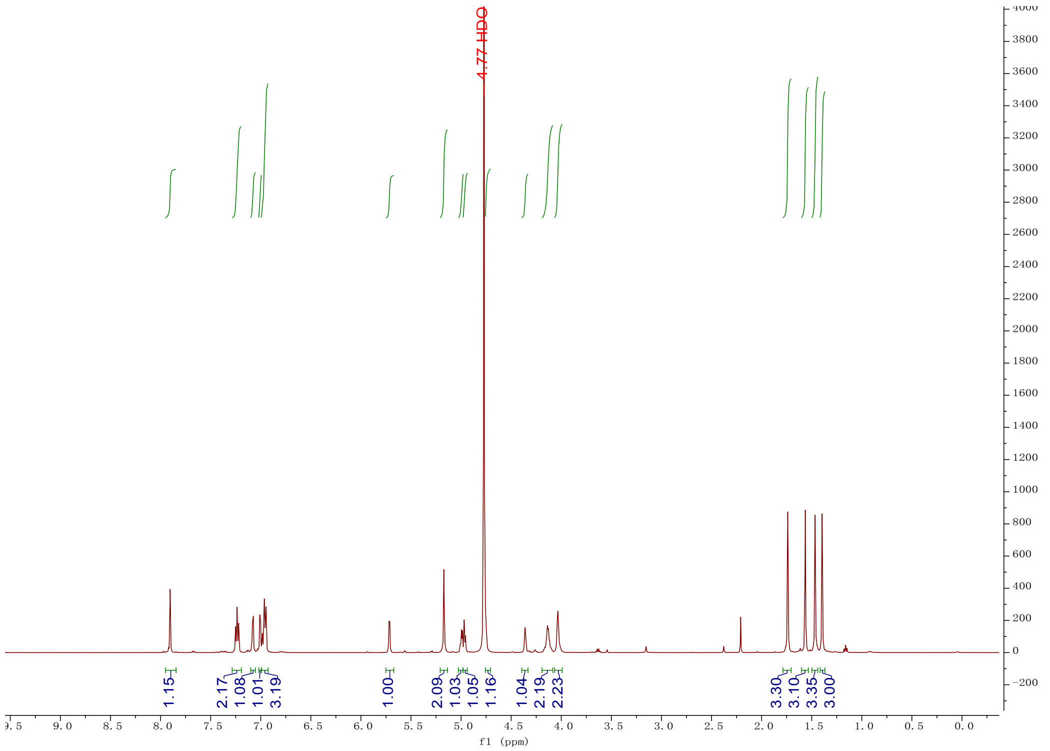

4.77 HDO
1.15
2.17
1.08
1.01
3.19
1.00
2.09
1.03
1.05
1.16
1.04
2.19
2.23
3.30
3.10
3.35
3.00
f1 (ppm)

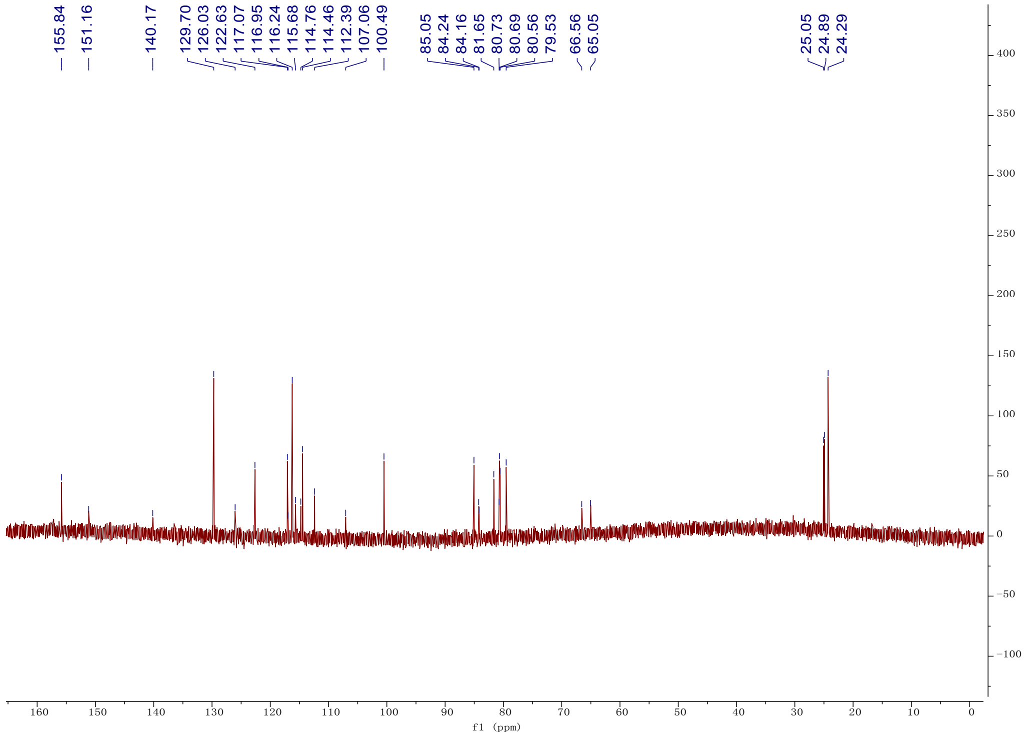

155.84
151.16
140.17
129.70
126.03
122.63
117.07
116.95
116.24
115.68
114.76
114.46
112.39
107.06
100.49
85.05
84.24
84.16
81.65
80.73
80.69
80.56
79.53
66.56
65.05
25.05
24.89
24.29
f1 (ppm)

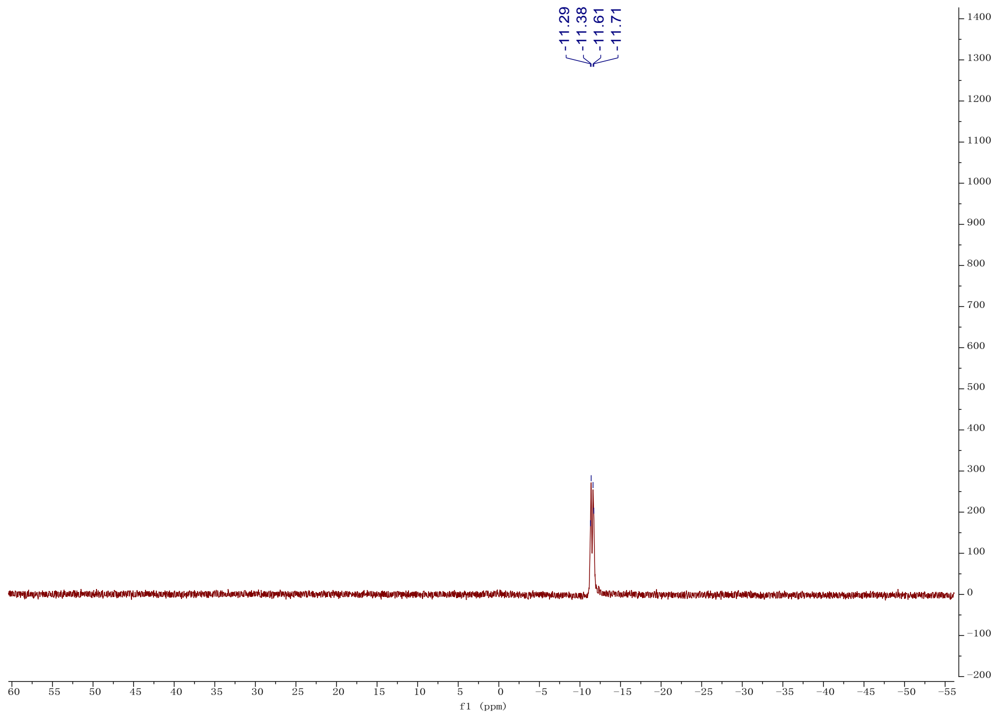
-11.29
-11.38
-11.61
-11.71
f1 (ppm)

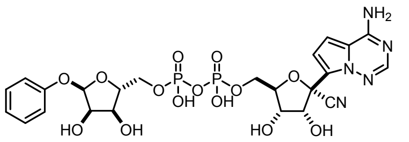
NH$_2$
N
N
N
O
O
O
O
P
P
O
O
O
OH
OH
HO
OH
HO
OH
CN

Ph-NDPr
4

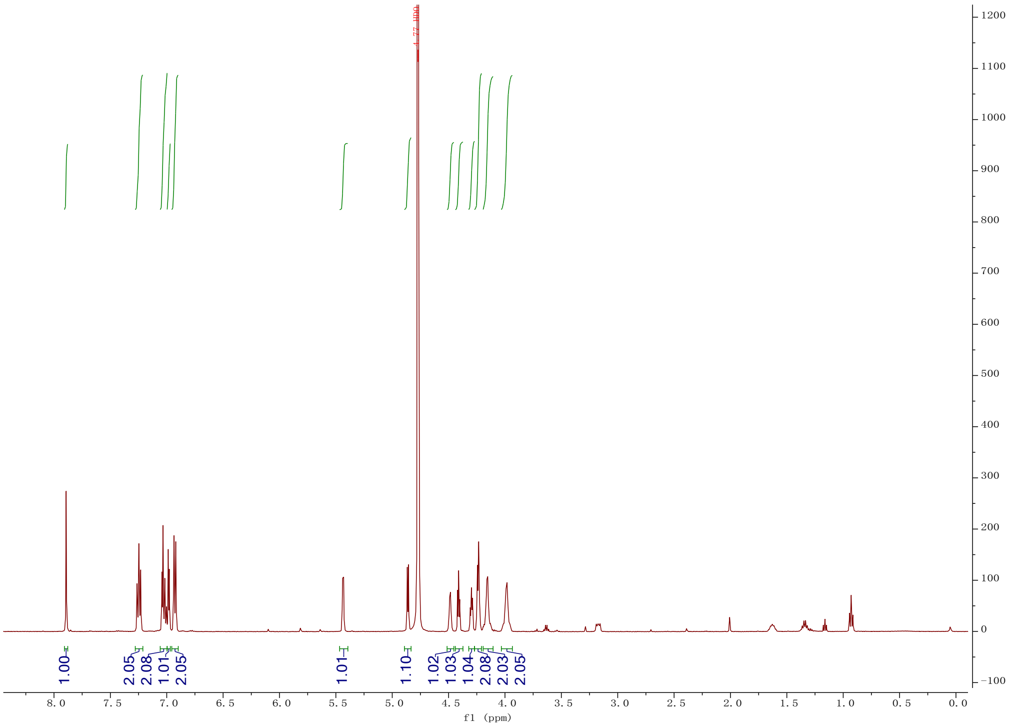
1.00
2.05
2.08
1.01
2.05
1.01
1.10
1.02
1.03
1.04
2.08
2.03
2.05
8.0 7.5 7.0 6.5 6.0 5.5 5.0 4.5 4.0 3.5 3.0 2.5 2.0 1.5 1.0 0.5 0.0
f1 (ppm)
1200 1100 1000 900 800 700 600 500 400 300 200 100 0 -100

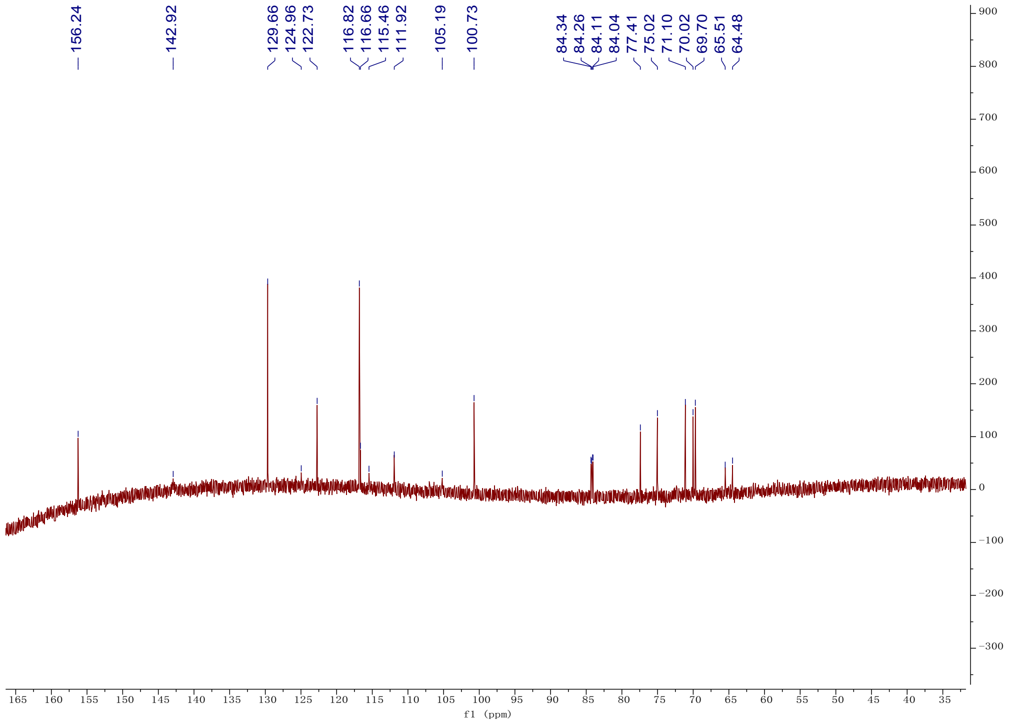
156.24
142.92
129.66
124.96
122.73
116.82
116.66
115.46
111.92
105.19
100.73
84.34
84.26
84.11
84.04
77.41
75.02
71.10
70.02
69.70
65.51
64.48
165 160 155 150 145 140 135 130 125 120 115 110 105 100 95 90 85 80 75 70 65 60 55 50 45 40 35
f1 (ppm)
900 800 700 600 500 400 300 200 100 0 -100 -200 -300

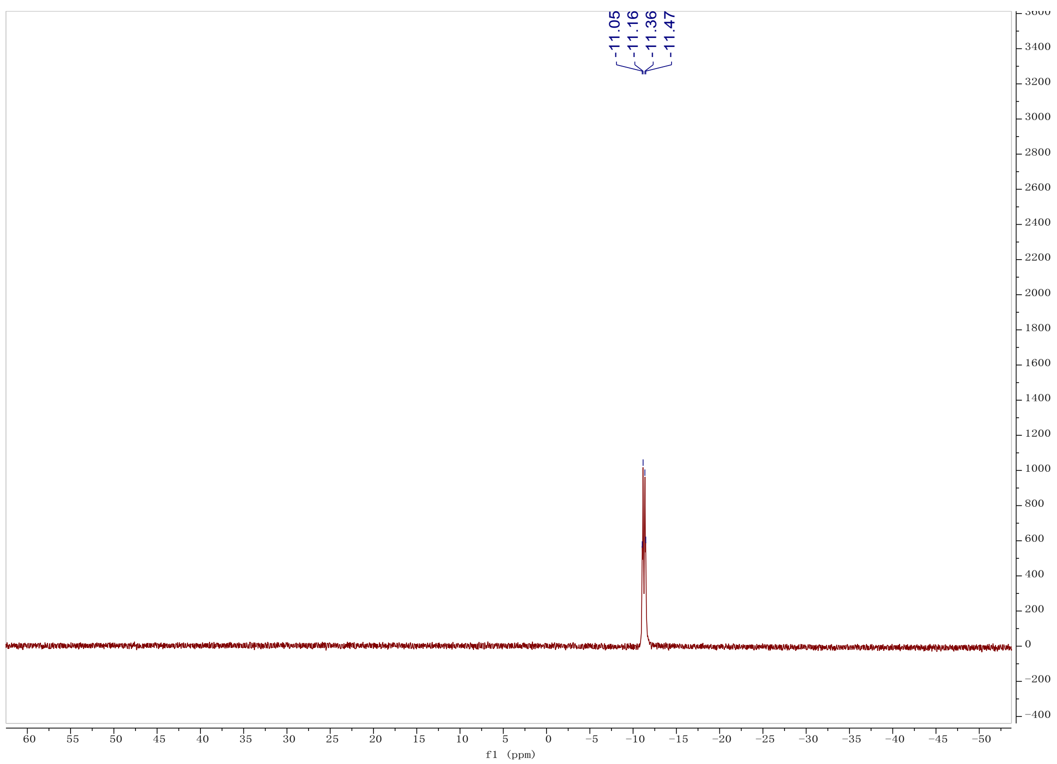
-11.05
-11.16
-11.36
-11.47
f1 (ppm)

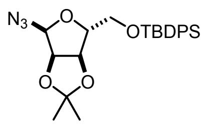
N3
O
OTBDPS
O
O

23

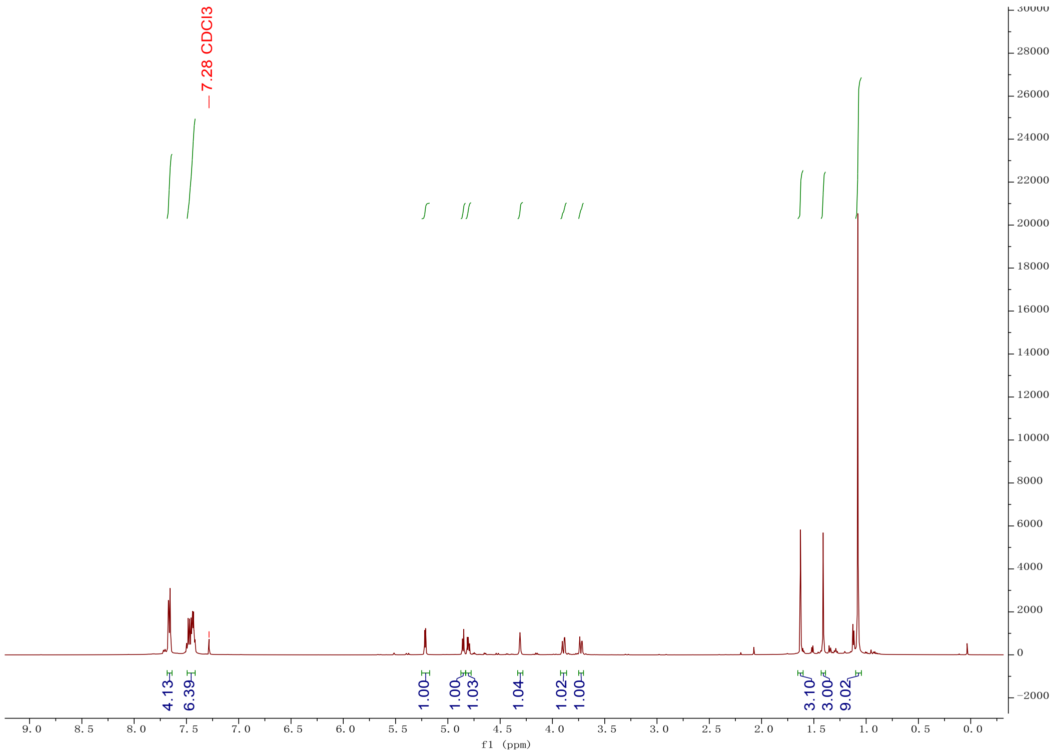
7.28 CDCl3
4.13
6.39
1.00
1.00
1.03
1.04
1.02
1.00
3.10
3.00
9.02
f1 (ppm)

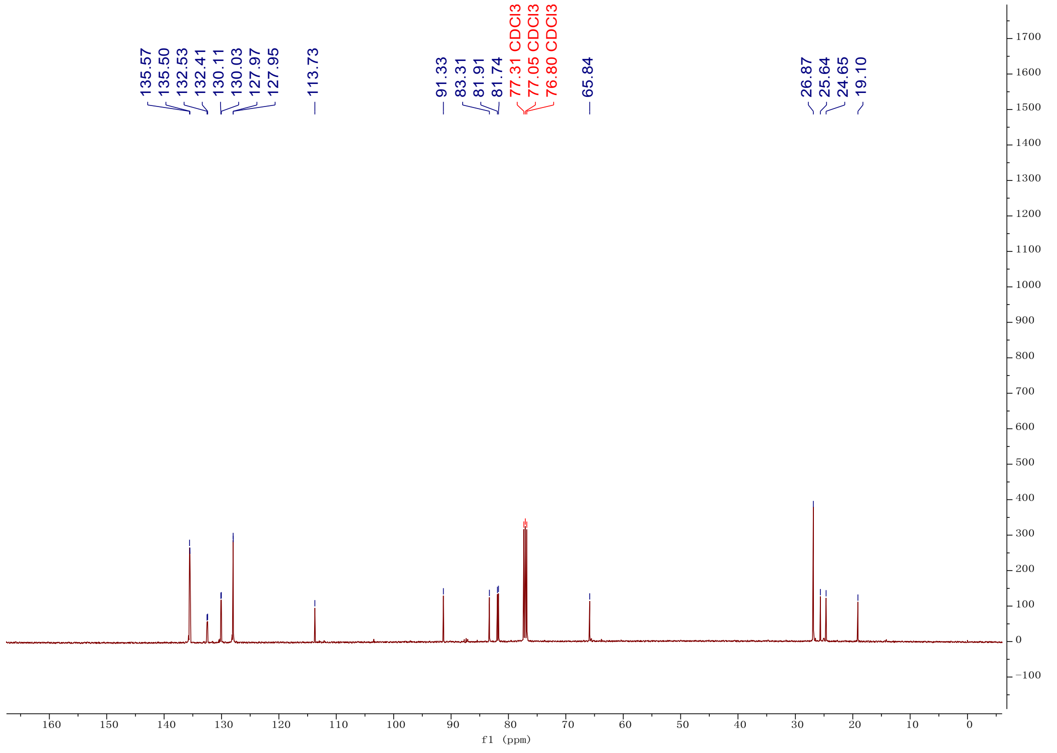
135.57
135.50
132.53
132.41
130.11
130.03
127.97
127.95
113.73
91.33
83.31
81.91
81.74
77.31 CDCl3
77.05 CDCl3
76.80 CDCl3
65.84
26.87
25.64
24.65
19.10
f1 (ppm)

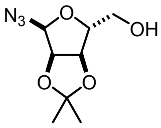

24

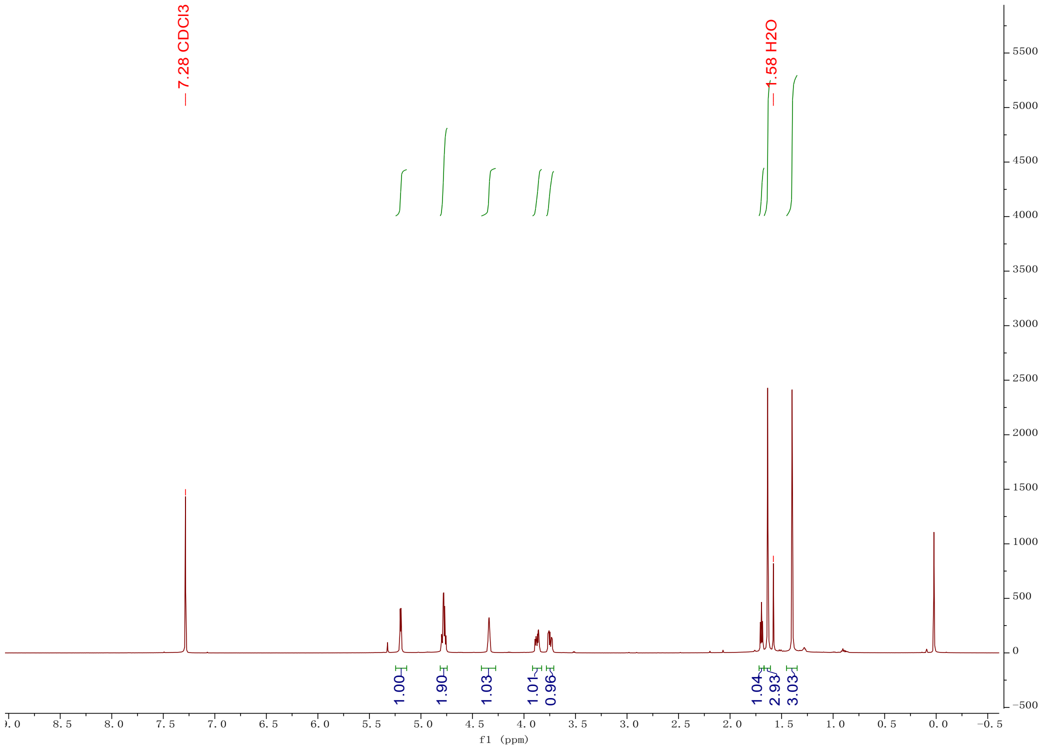

7.28 CDCl3
1.58 H2O
1.00
1.90
1.03
1.01
0.96
1.04
2.93
3.03
f1 (ppm)

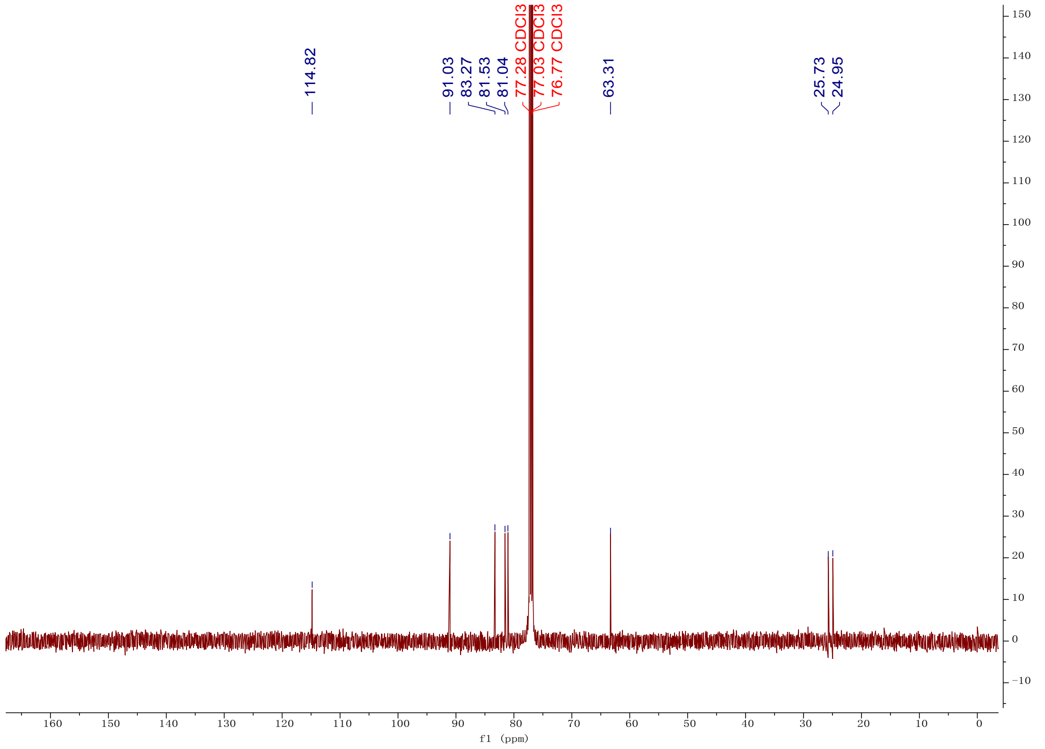

114.82
91.03
83.27
81.53
81.04
77.28 CDCl3
77.03 CDCl3
76.77 CDCl3
63.31
25.73
24.95
f1 (ppm)

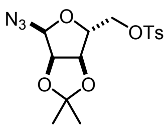

25

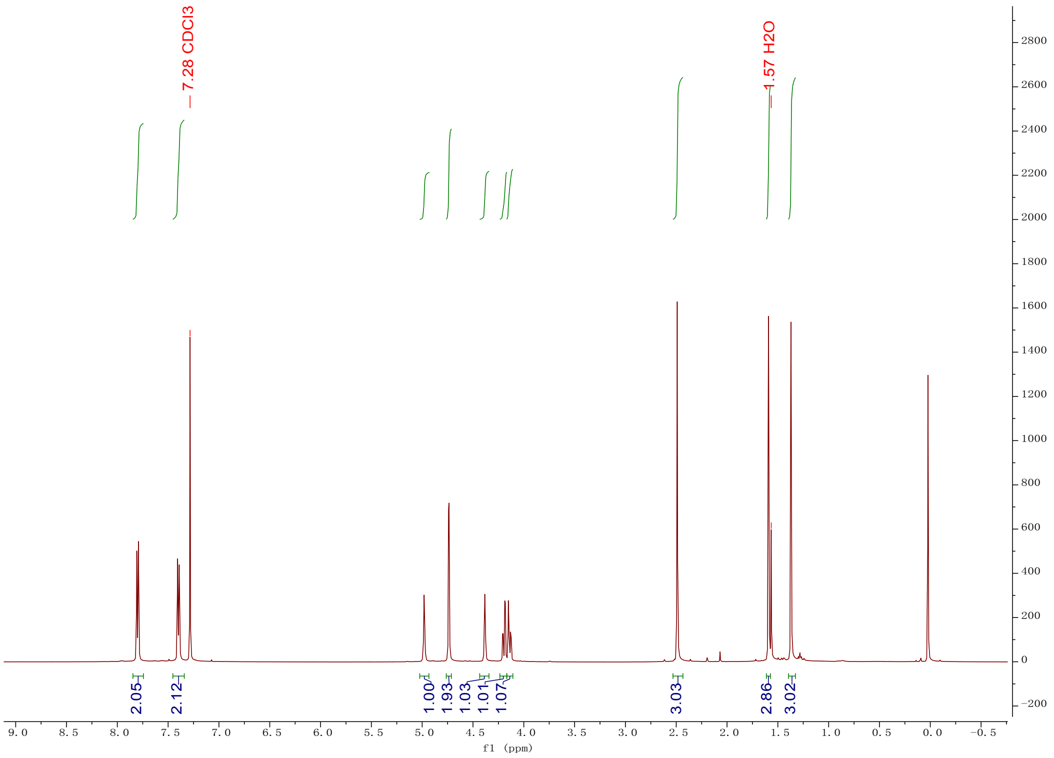
7.28 CDCl3
1.57 H2O
2.05
2.12
1.00
1.93
1.03
1.01
1.07
3.03
2.86
3.02
f1 (ppm)

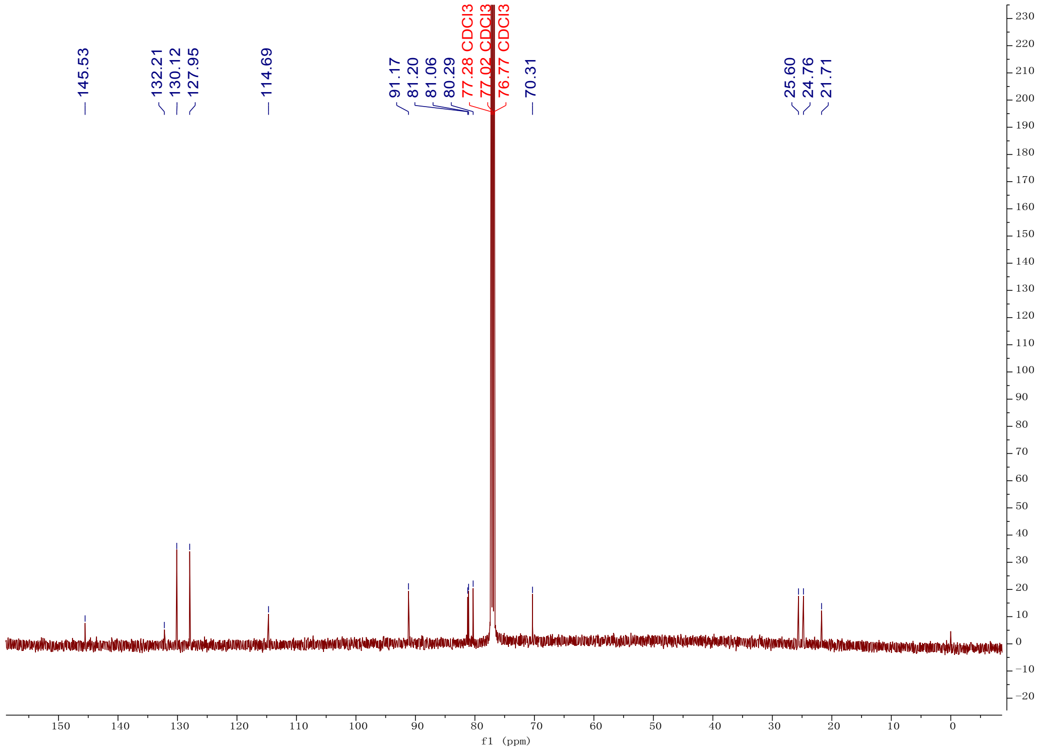
145.53
132.21
130.12
127.95
114.69
91.17
81.20
81.06
80.29
77.28 CDCl3
77.02 CDCl3
76.77 CDCl3
70.31
25.60
24.76
21.71
f1 (ppm)

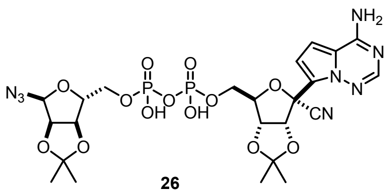

26

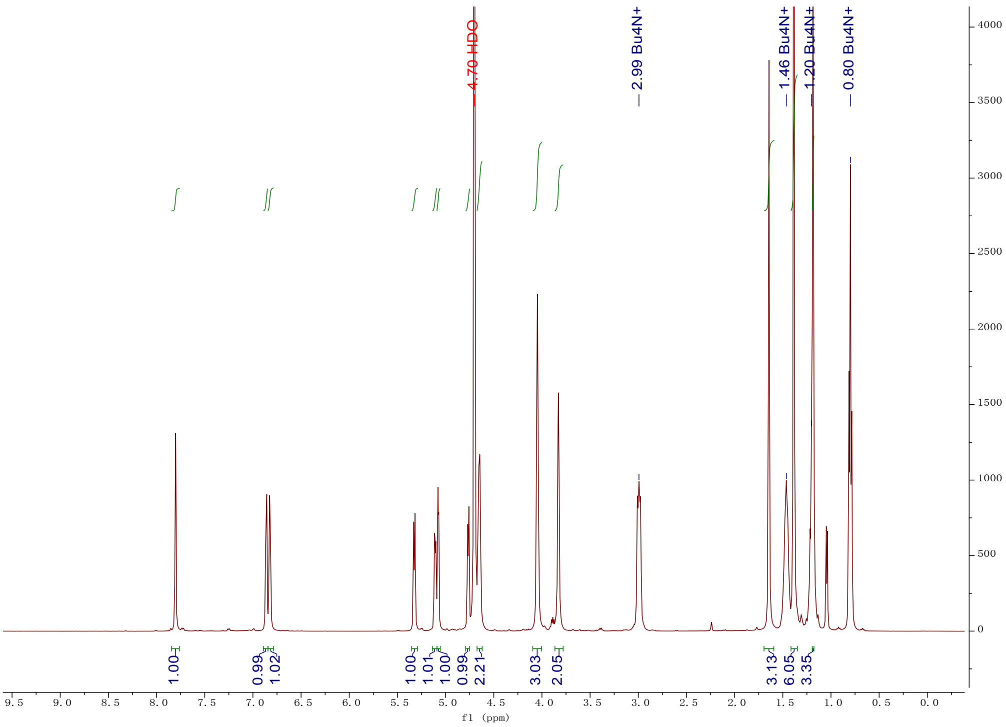
4.70 HDO
2.99 Bu4N+
1.46 Bu4N+
1.20 Bu4N+
0.80 Bu4N+
1.00
0.99
1.02
1.00
1.01
1.00
0.99
2.21
3.03
2.05
3.13
6.05
3.35
f1 (ppm)

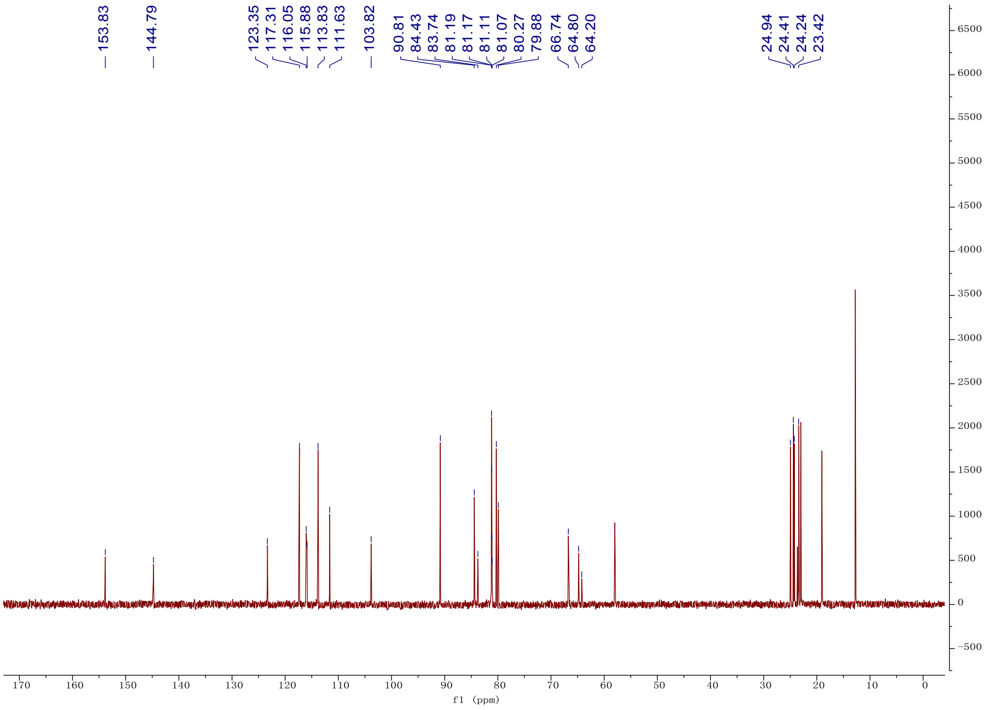
153.83
144.79
123.35
117.31
116.05
115.88
113.83
111.63
103.82
90.81
84.43
83.74
81.19
81.17
81.11
81.07
80.27
79.88
66.74
64.80
64.20
24.94
24.41
24.24
23.42
f1 (ppm)

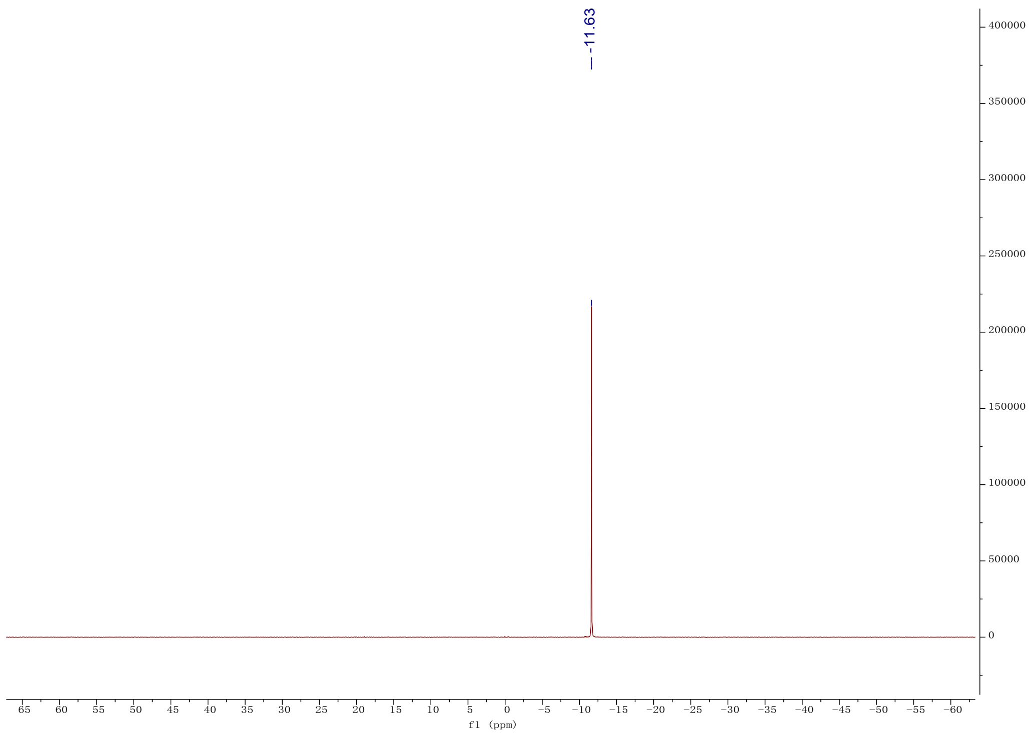

-11.63
400000
350000
300000
250000
200000
150000
100000
50000
0
65 60 55 50 45 40 35 30 25 20 15 10 5 0 -5 -10 -15 -20 -25 -30 -35 -40 -45 -50 -55 -60
f1 (ppm)

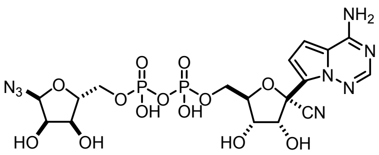

27

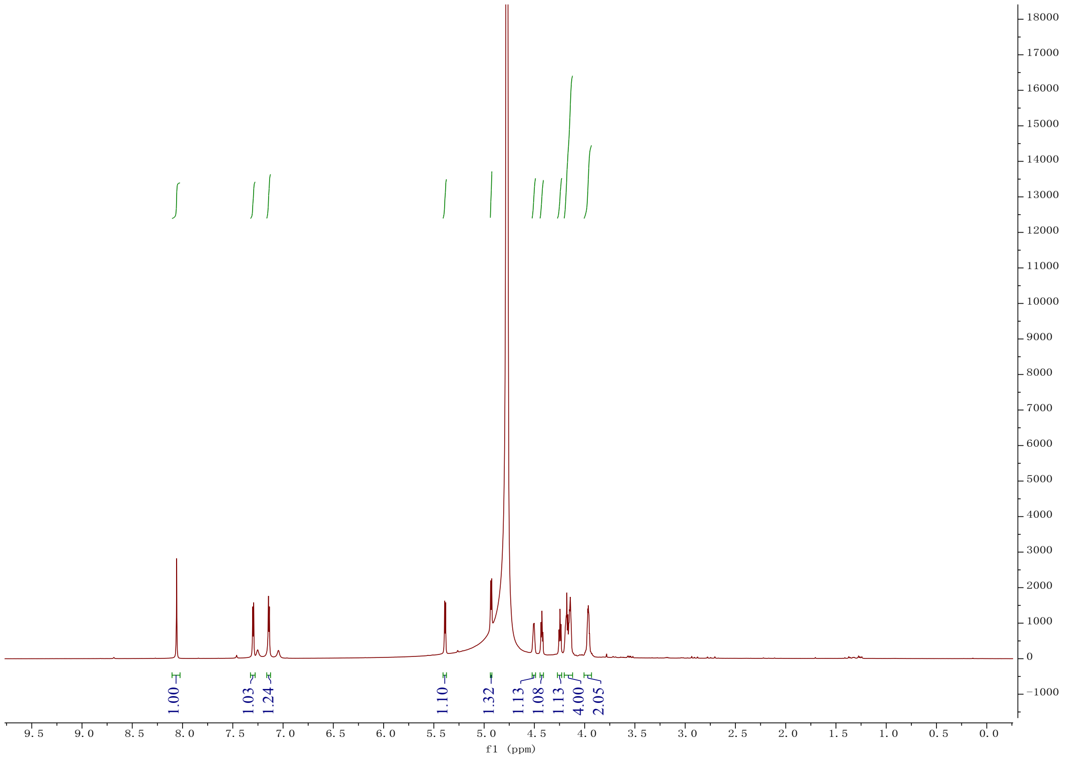
1.00
1.03
1.24
1.10
1.32
1.13
1.08
1.13
4.00
2.05
f1 (ppm)

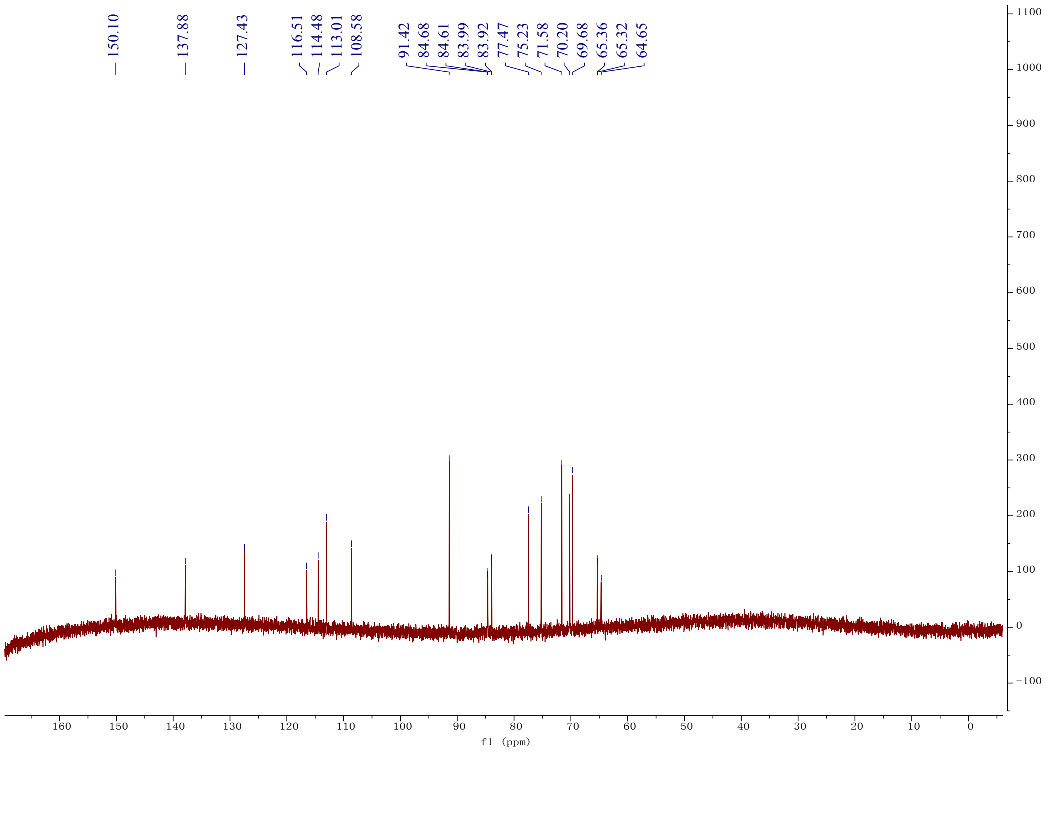
150.10
137.88
127.43
116.51
114.48
113.01
108.58
91.42
84.68
84.61
83.99
83.92
77.47
75.23
71.58
70.20
69.68
65.36
65.32
64.65
f1 (ppm)

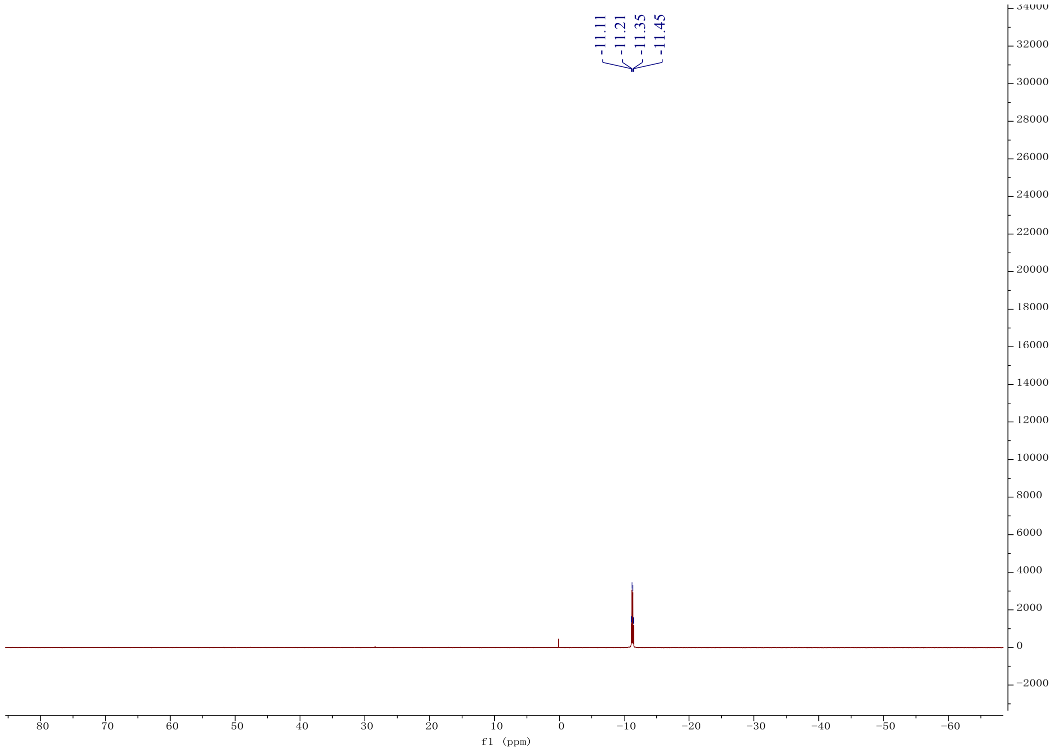

-11.11
-11.21
-11.35
-11.45
34000
32000
30000
28000
26000
24000
22000
20000
18000
16000
14000
12000
10000
8000
6000
4000
2000
0
-2000
80
70
60
50
40
30
20
10
0
-10
-20
-30
-40
-50
-60
f1 (ppm)

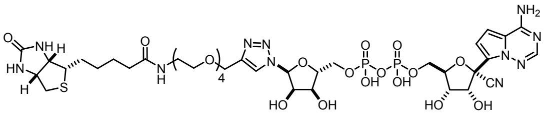

Biotin-NDPr
7

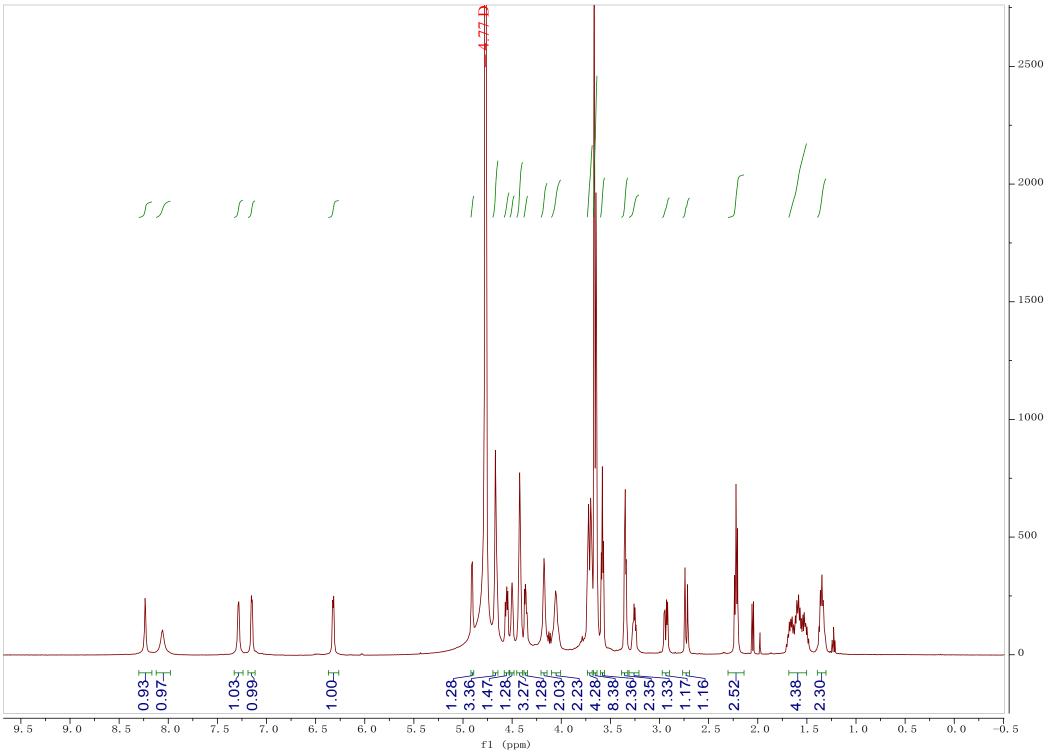

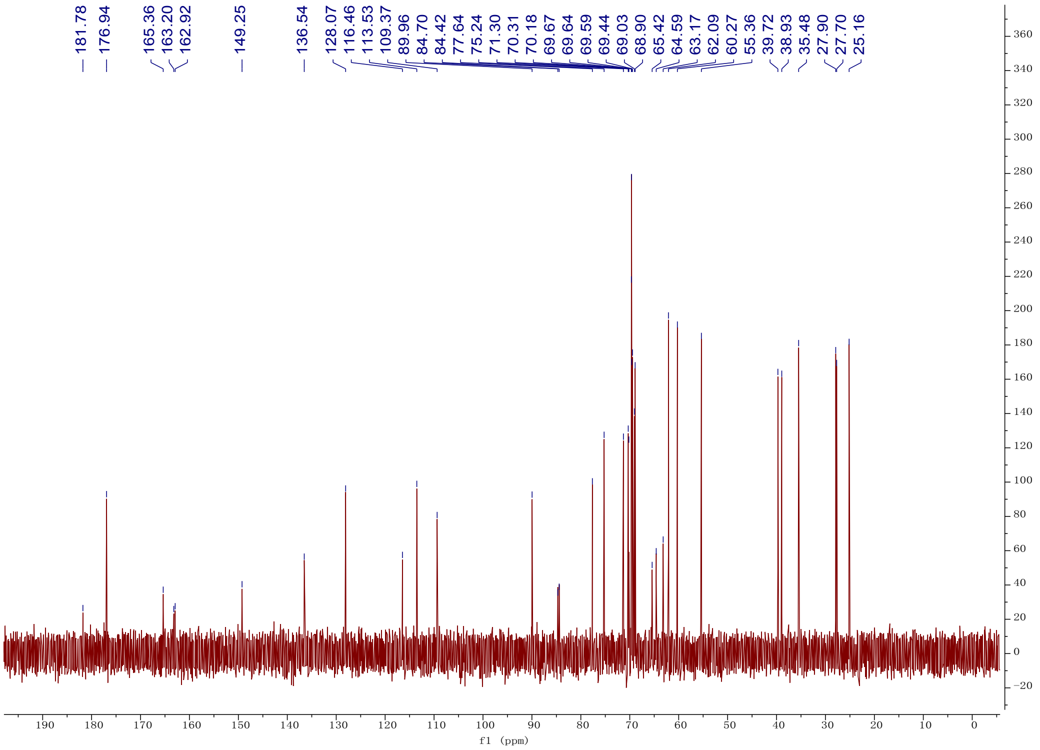

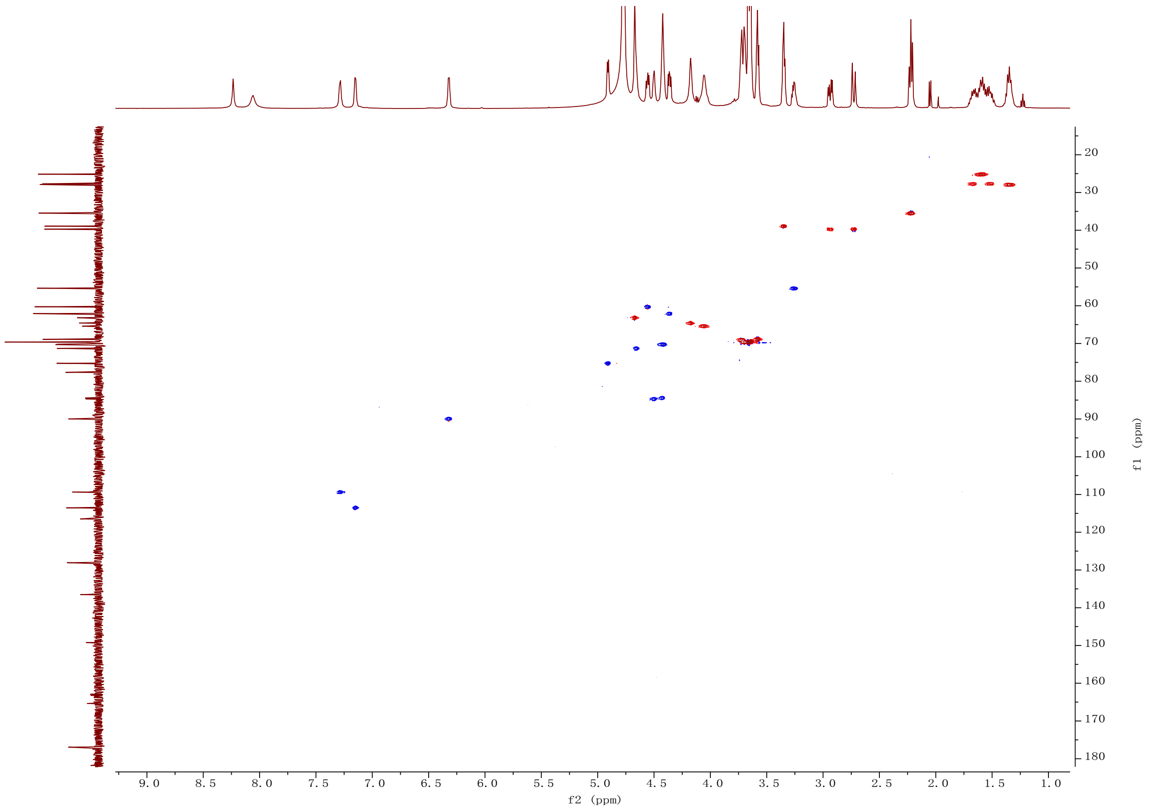
f1 (ppm)
f2 (ppm)

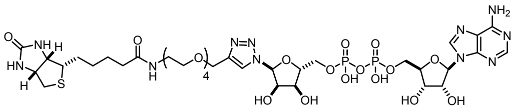

Biotin-ADPr

5

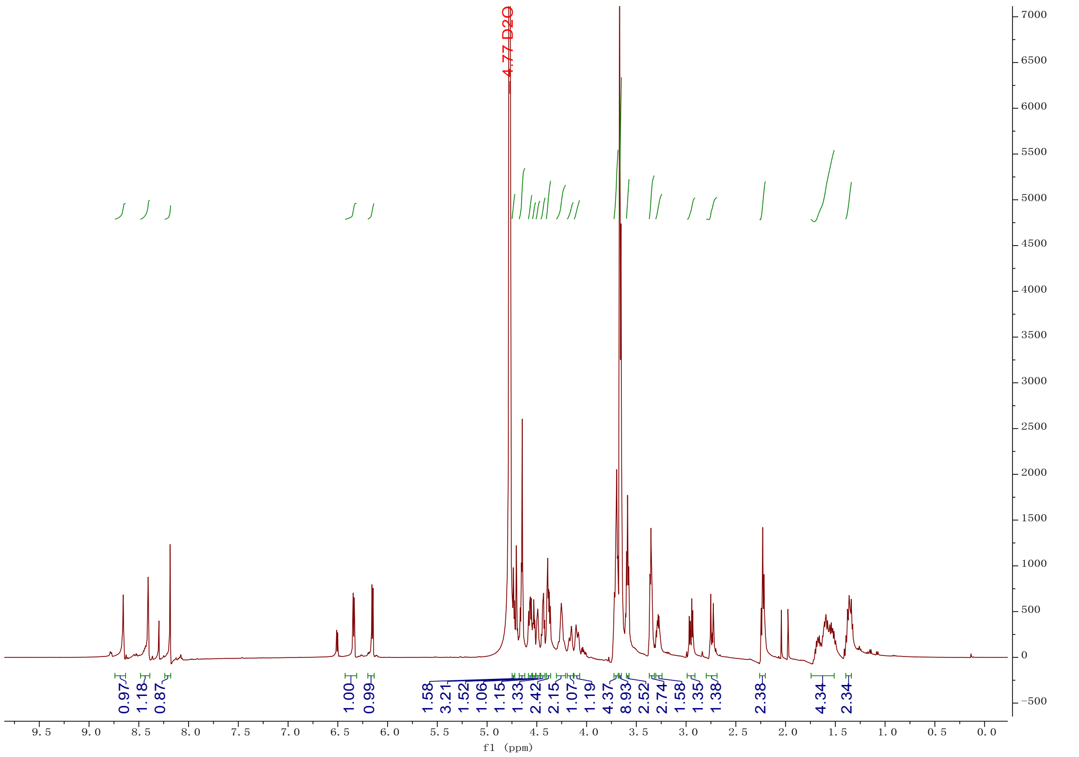

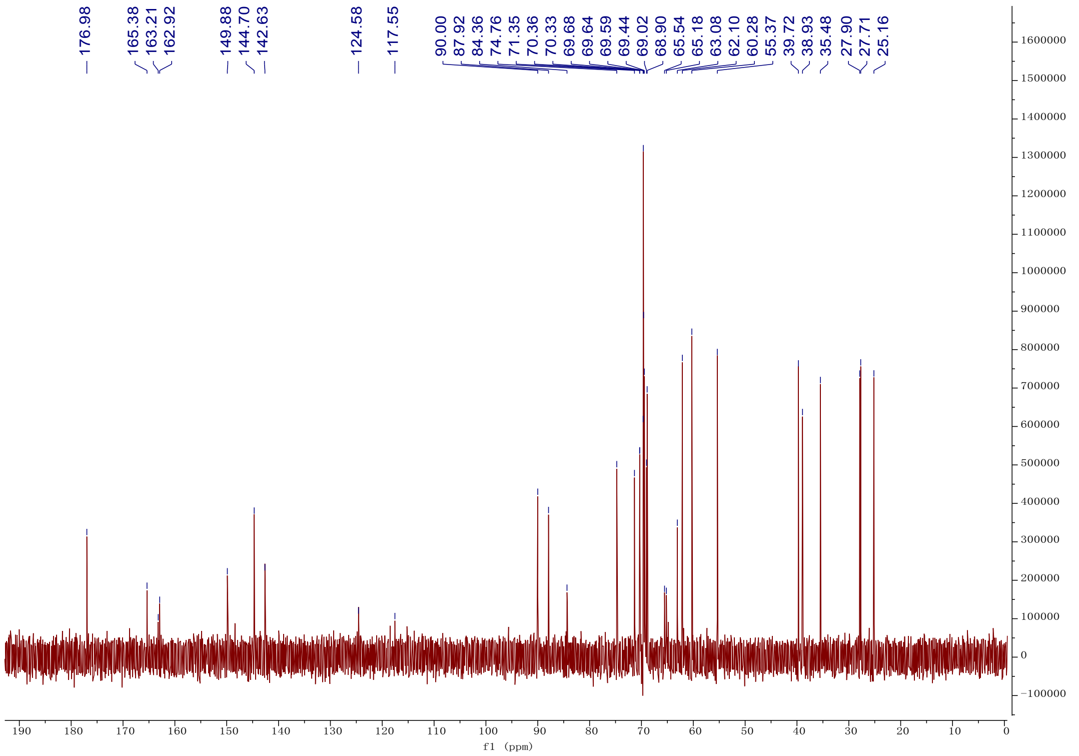

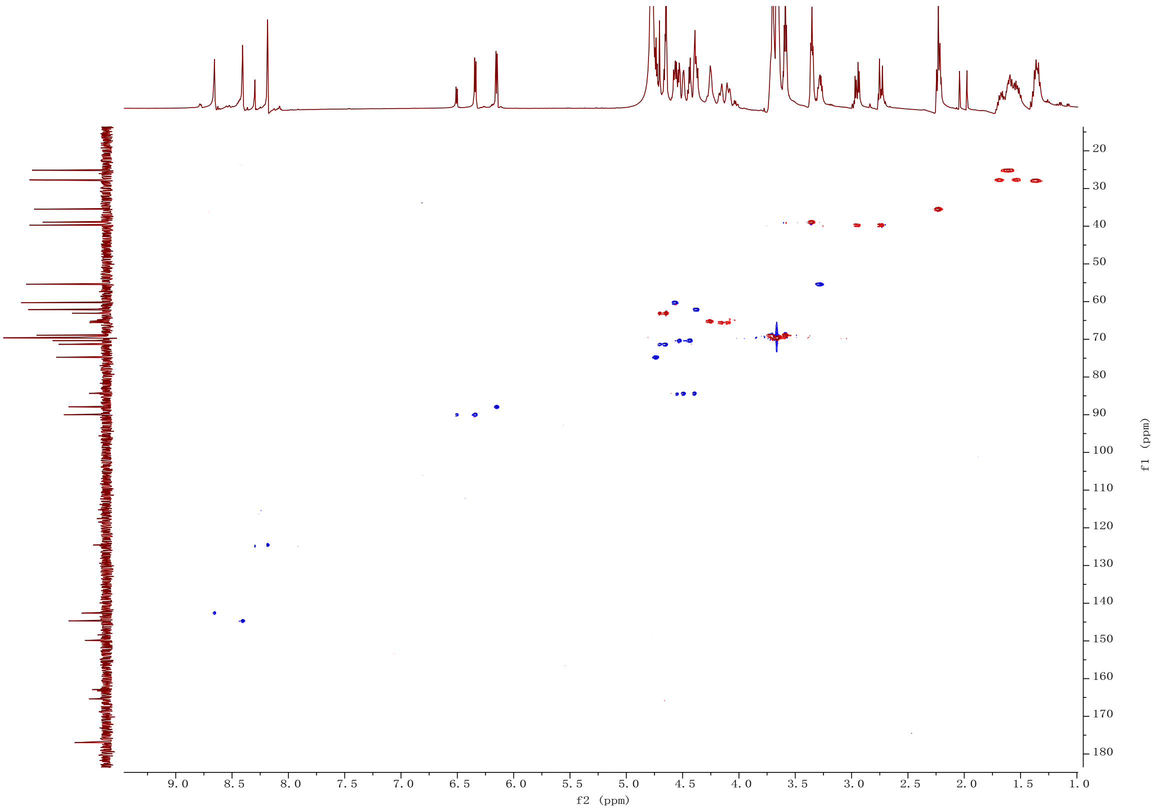
f2 (ppm)
f1 (ppm)

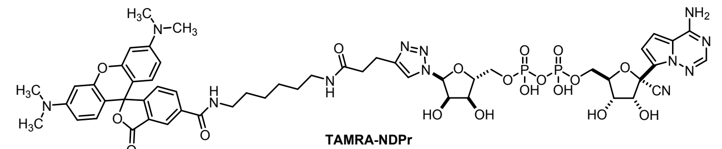
H3C
N−CH3
H3C
N
H3C
H
N
O
O
O
O
N
H
O
N=N
N
O
O
P
O
P
O
OH
OH
O
O
HO
OH
HO
OH
CN
NH2
N
N
N

TAMRA-NDPr

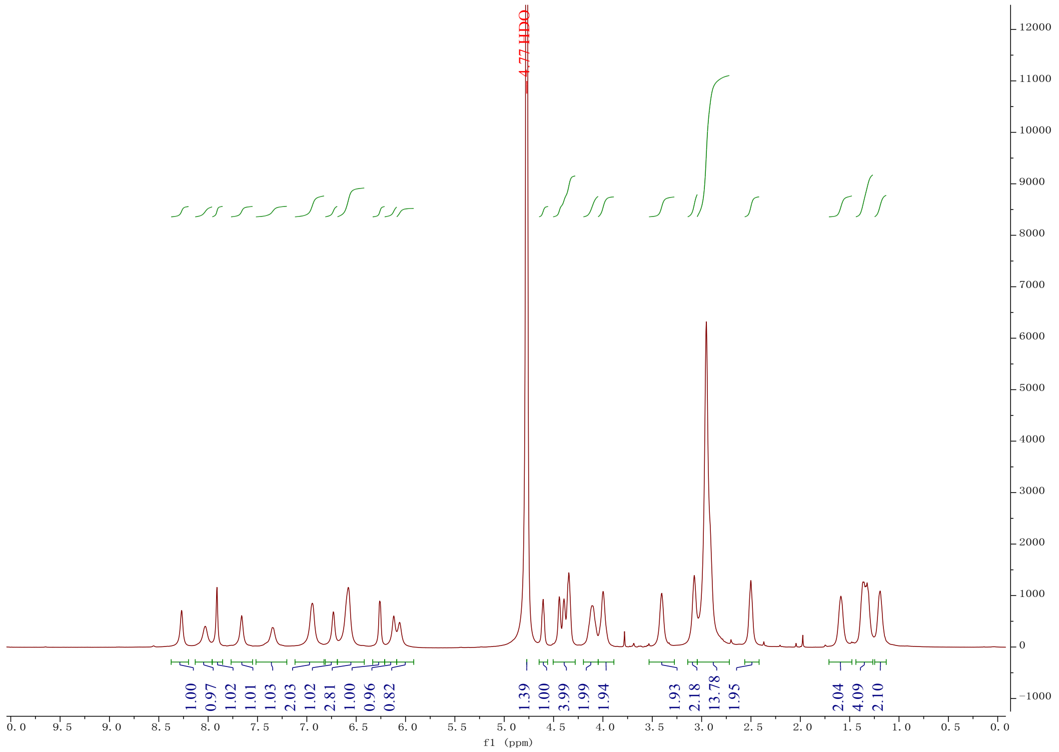
4.77 HDO
1.00
0.97
1.02
1.01
1.03
2.03
1.02
2.81
1.00
0.96
0.82
1.39
1.00
3.99
1.99
1.94
1.93
2.18
13.78
1.95
2.04
4.09
2.10
f1 (ppm)

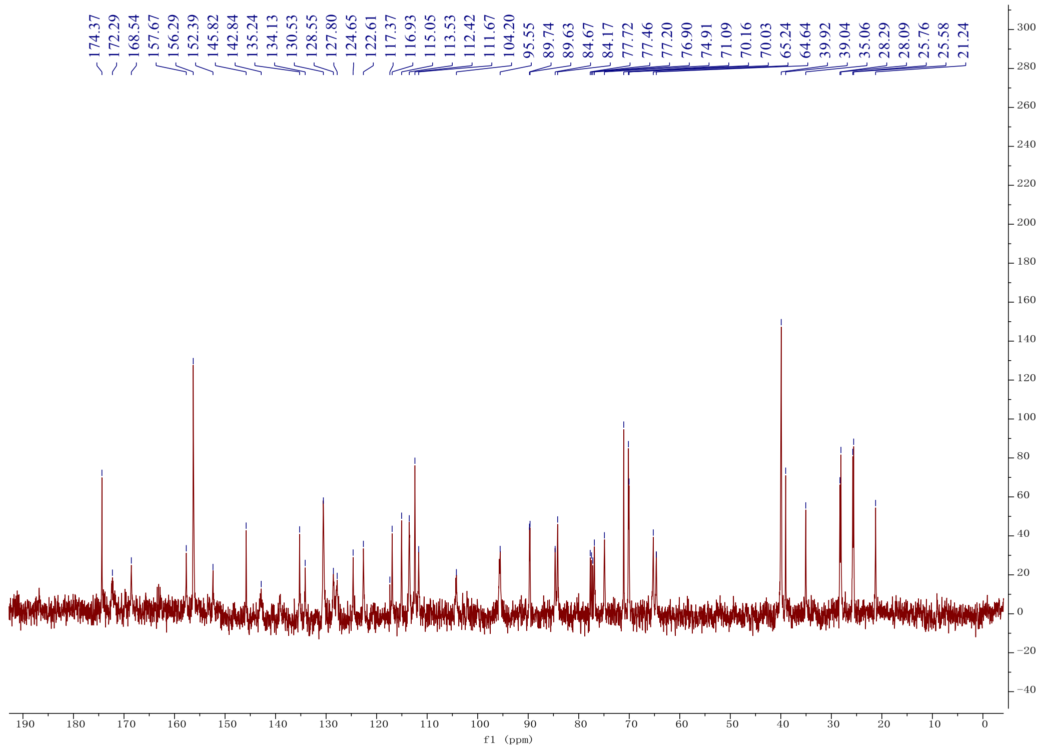
174.37
172.29
168.54
157.67
156.29
152.39
145.82
142.84
135.24
134.13
130.53
128.55
127.80
124.65
122.61
117.37
116.93
115.05
113.53
112.42
111.67
104.20
95.55
89.74
89.63
84.67
84.17
77.72
77.46
77.20
76.90
74.91
71.09
70.16
70.03
65.24
64.64
39.92
39.04
35.06
28.29
28.09
25.76
25.58
21.24
f1 (ppm)

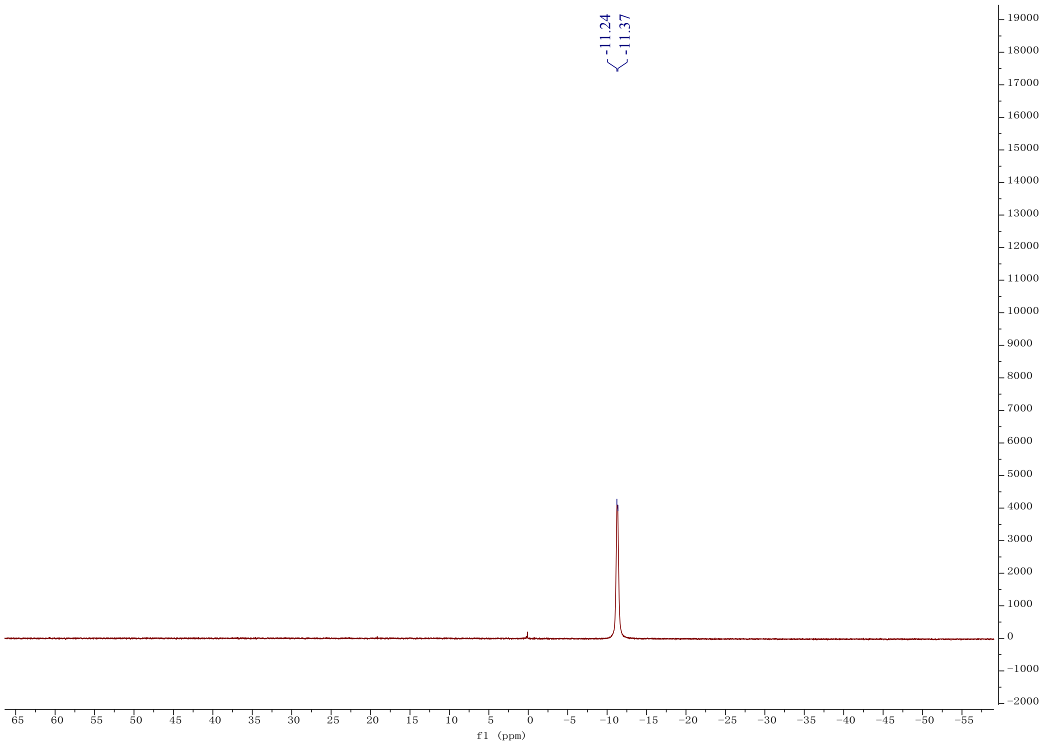
-11.24
-11.37
f1 (ppm)

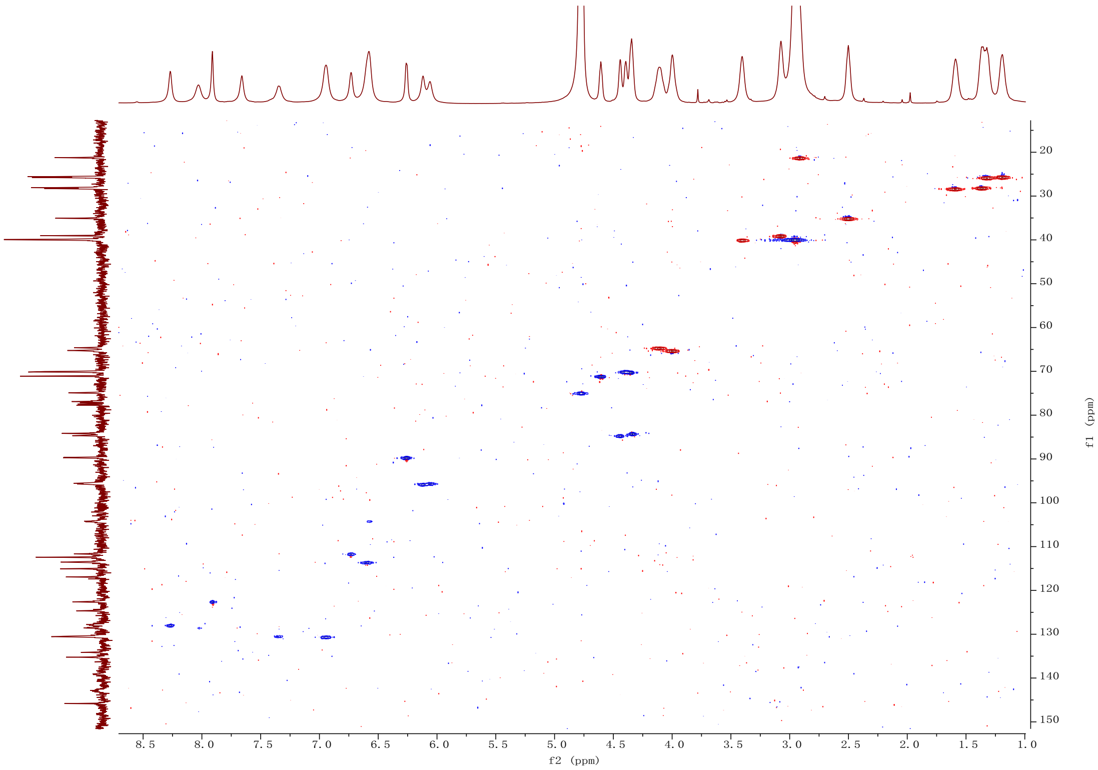
f2 (ppm)
f1 (ppm)

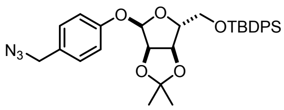

28

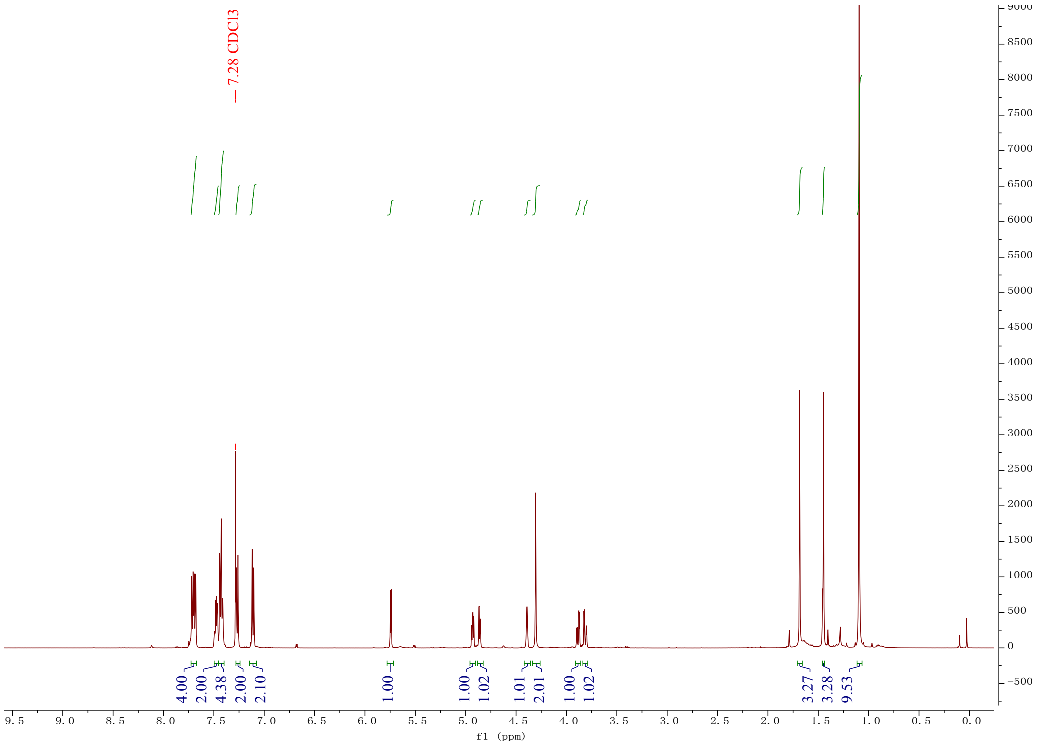

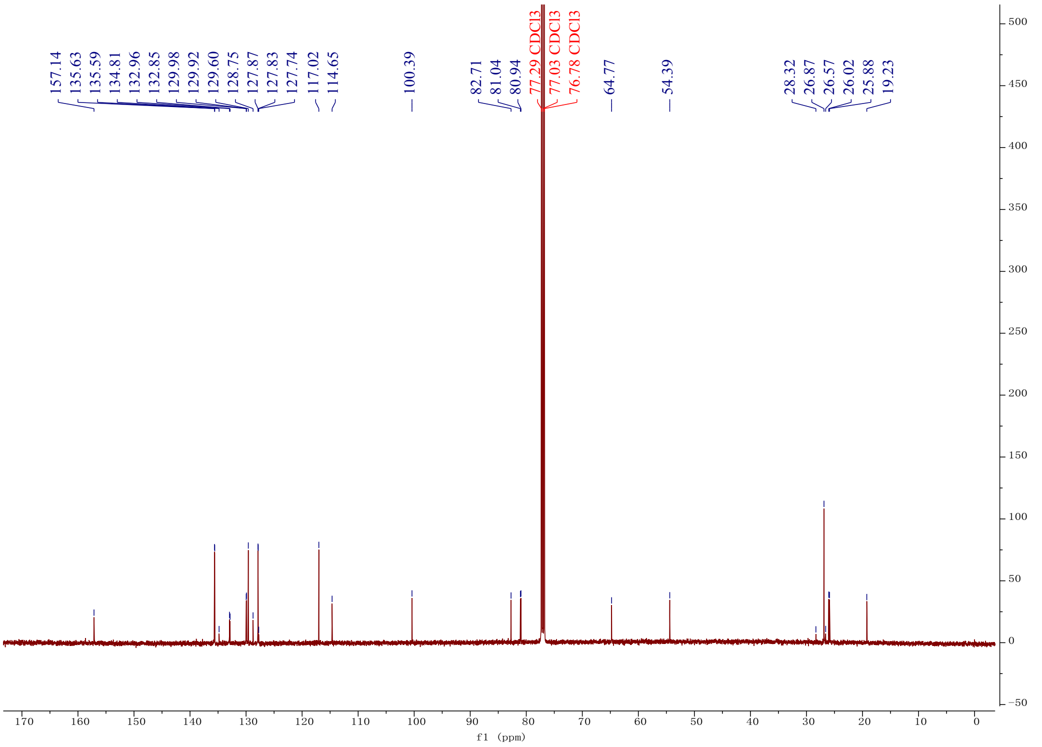

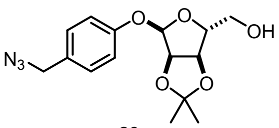

29

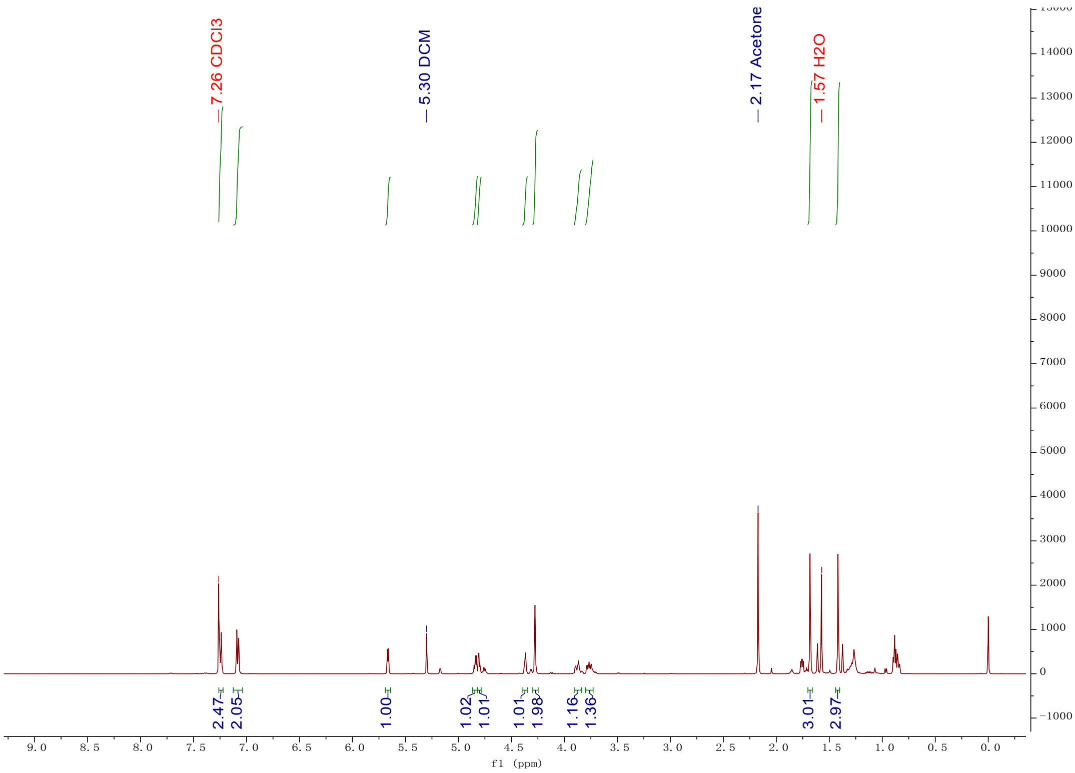

7.26 CDCl3
5.30 DCM
2.17 Acetone
1.57 H2O
2.47
2.05
1.00
1.02
1.01
1.01
1.98
1.16
1.36
3.01
2.97
f1 (ppm)

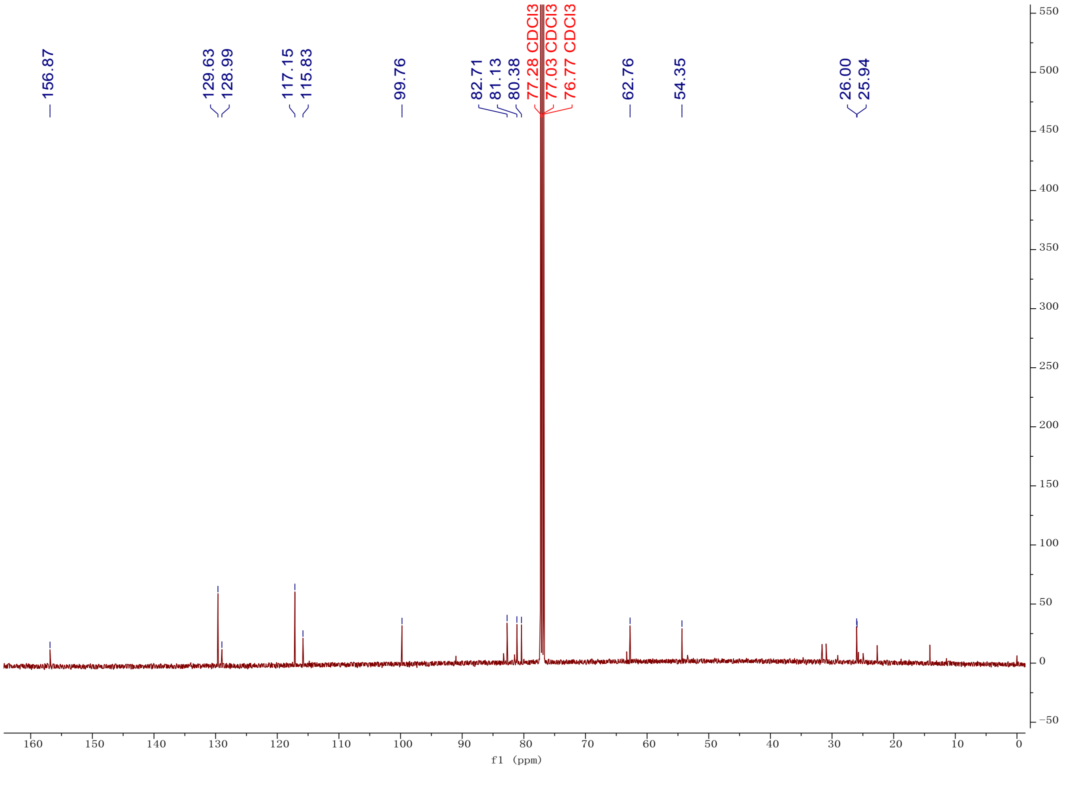

156.87
129.63
128.99
117.15
115.83
99.76
82.71
81.13
80.38
77.28 CDCl3
77.03 CDCl3
76.77 CDCl3
62.76
54.35
26.00
25.94
f1 (ppm)

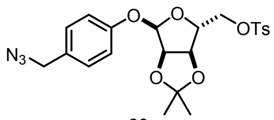
N3
O
O
OTs
O
O

30

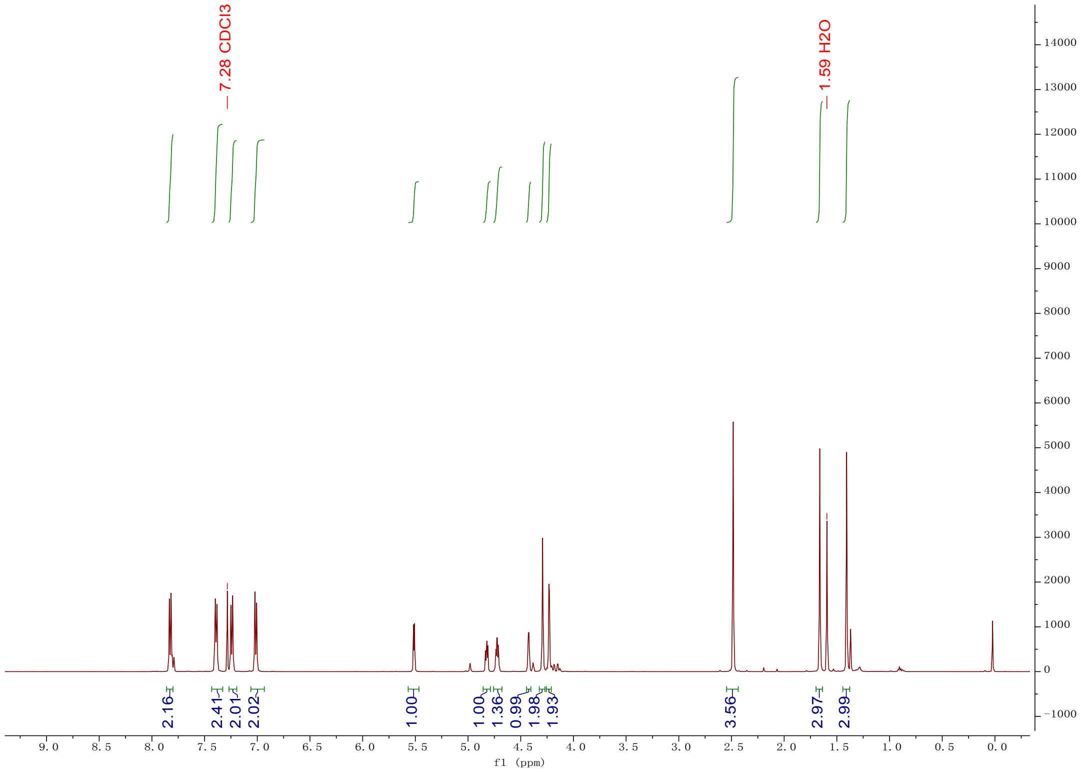
7.28 CDCl3
1.59 H2O
2.16
2.41
2.01
2.02
1.00
1.00
1.36
0.99
1.98
1.93
3.56
2.97
2.99
f1 (ppm)

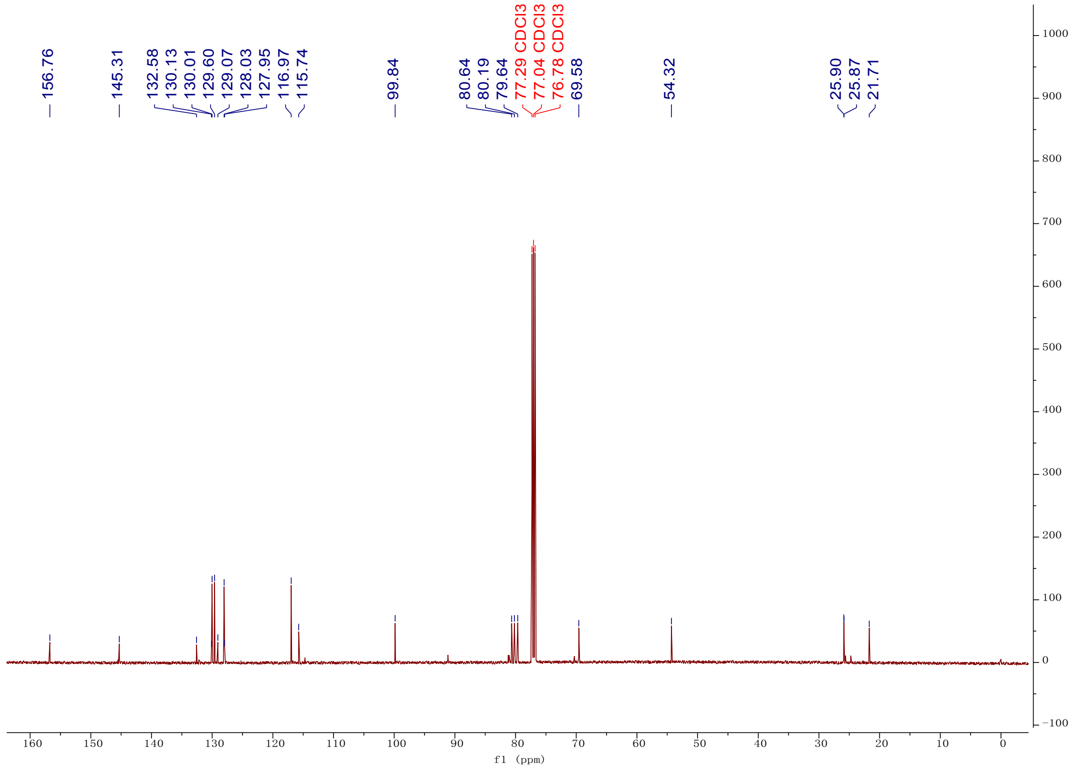
156.76
145.31
132.58
130.13
130.01
129.60
129.07
128.03
127.95
116.97
115.74
99.84
80.64
80.19
79.64
77.29 CDCl3
77.04 CDCl3
76.78 CDCl3
69.58
54.32
25.90
25.87
21.71
f1 (ppm)

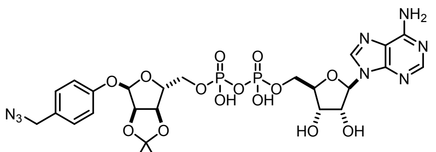

31

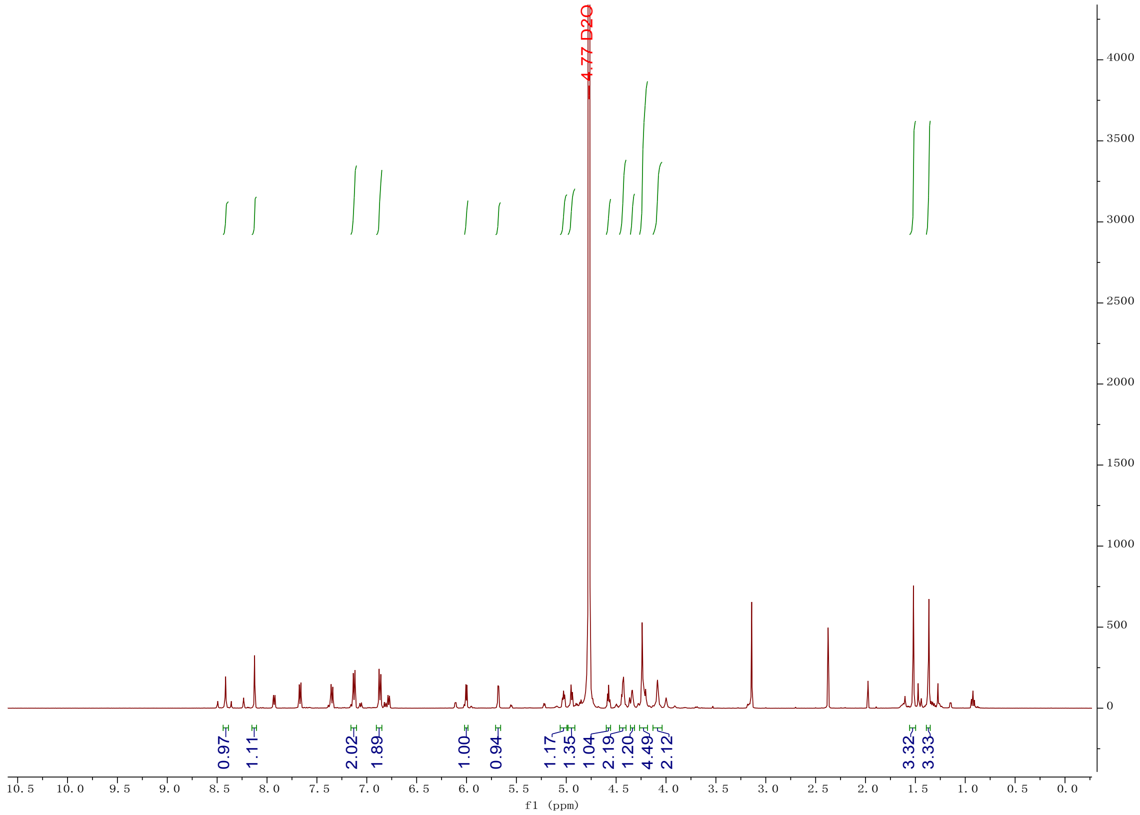
4.77 D2O
0.97
1.11
2.02
1.89
1.00
0.94
1.17
1.35
1.04
2.19
1.20
4.49
2.12
3.32
3.33
f1 (ppm)

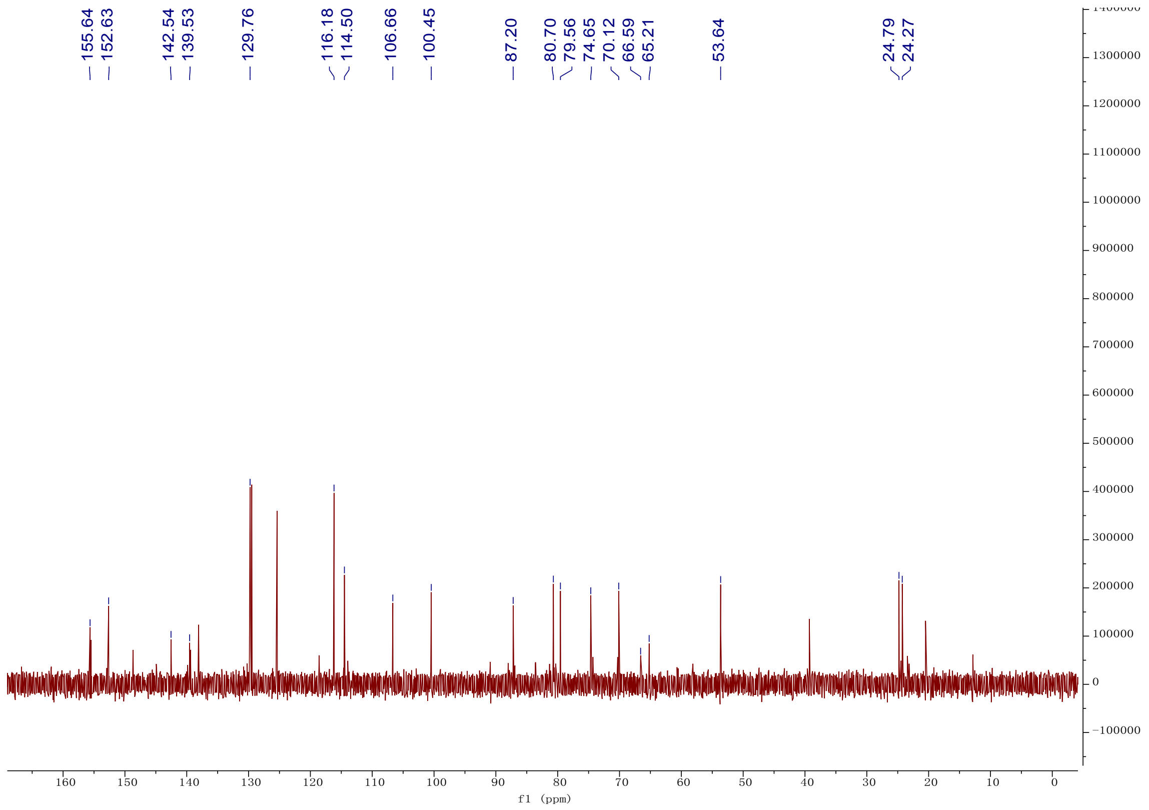
155.64
152.63
142.54
139.53
129.76
116.18
114.50
106.66
100.45
87.20
80.70
79.56
74.65
70.12
66.59
65.21
53.64
24.79
24.27
f1 (ppm)

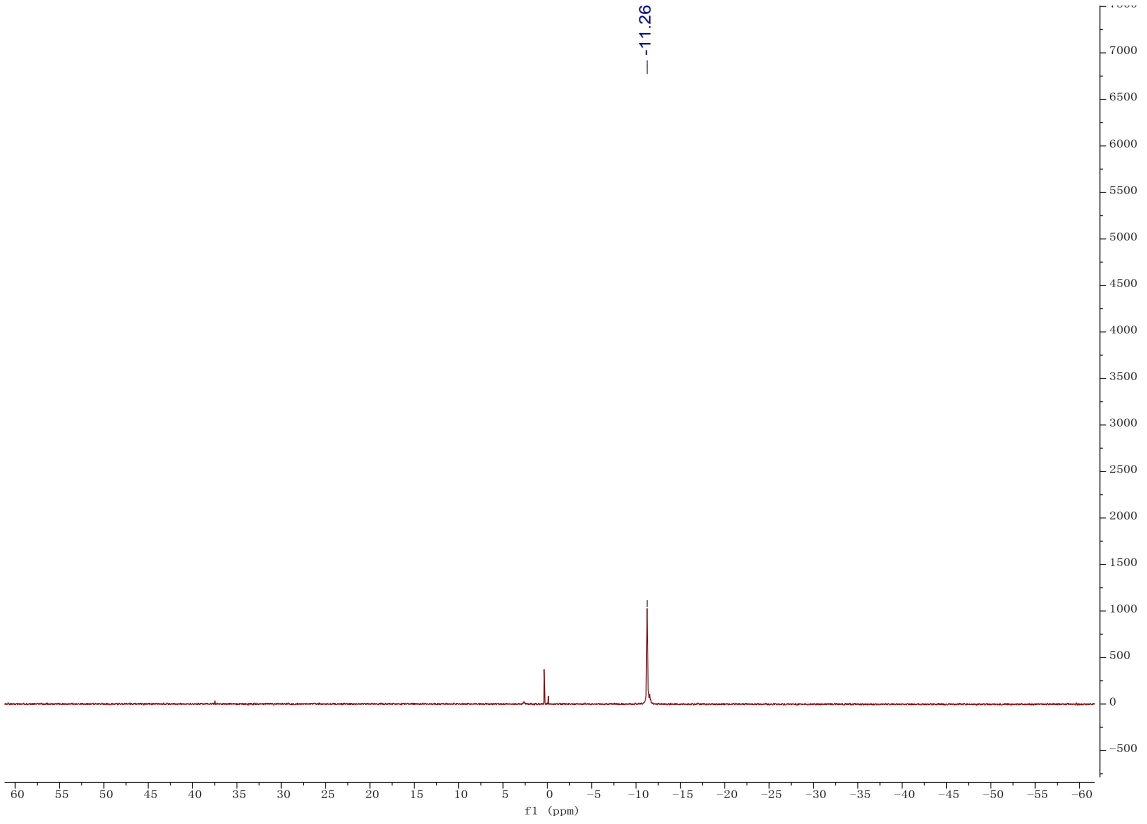
-11.26
f1 (ppm)

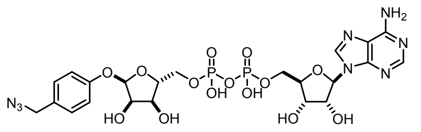
NH2
N
N
N
N
O
O
O
P
O
O
P
O
OH
OH
O
O
O
N3
HO
OH
HO
OH

32

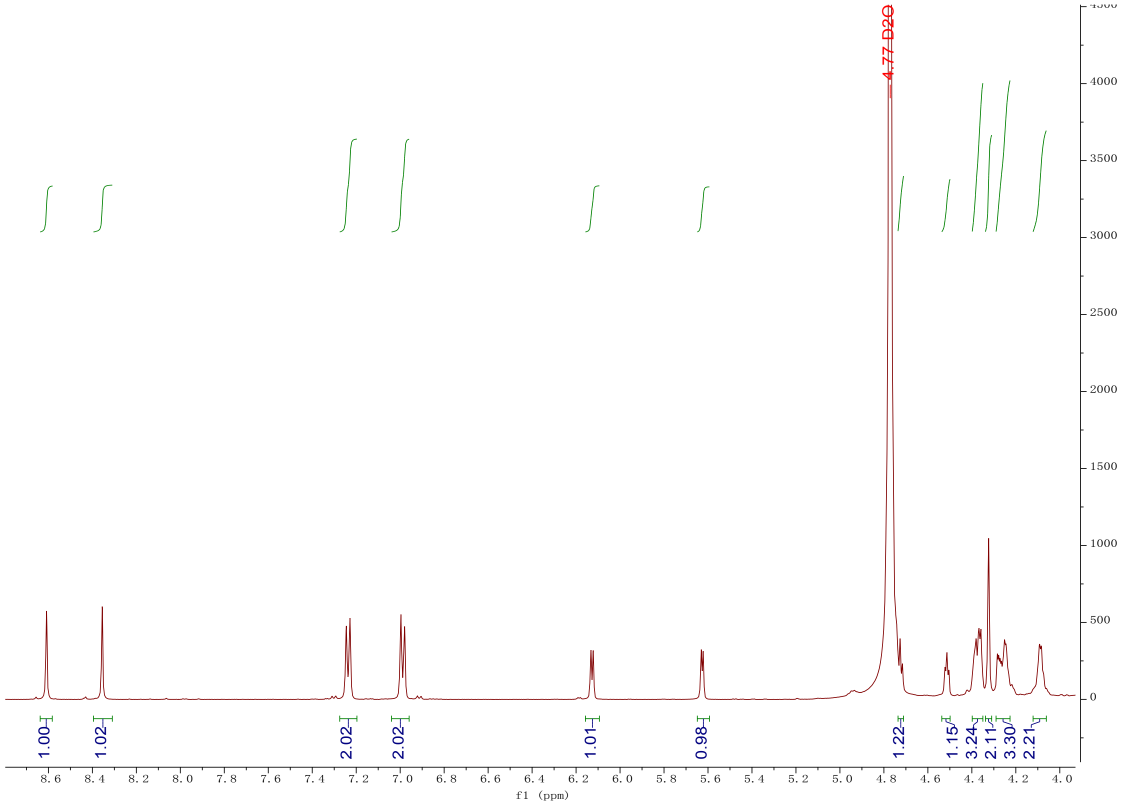
4.77 D2O
1.00
1.02
2.02
2.02
1.01
0.98
1.22
1.15
3.24
2.11
3.30
2.21
f1 (ppm)

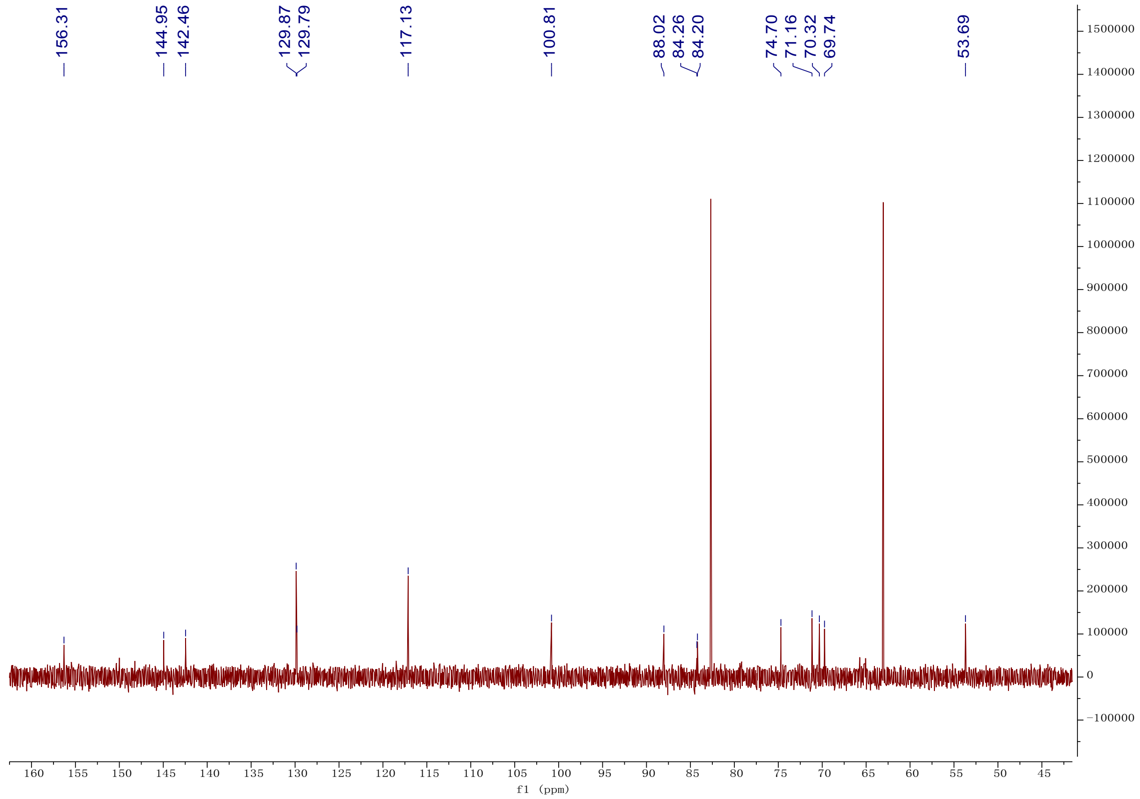
156.31
144.95
142.46
129.87
129.79
117.13
100.81
88.02
84.26
84.20
74.70
71.16
70.32
69.74
53.69
f1 (ppm)

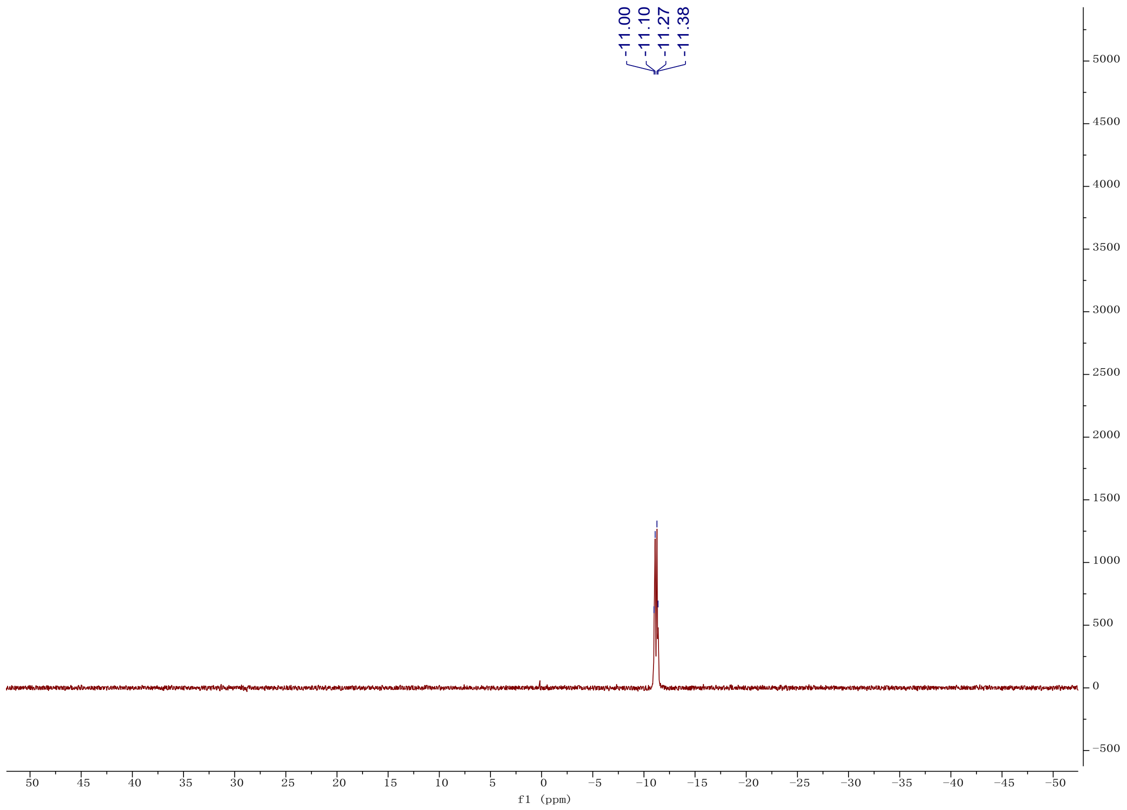

-11.00
-11.10
-11.27
-11.38
5000
4500
4000
3500
3000
2500
2000
1500
1000
500
0
-500
50 45 40 35 30 25 20 15 10 5 0 -5 -10 -15 -20 -25 -30 -35 -40 -45 -50
f1 (ppm)

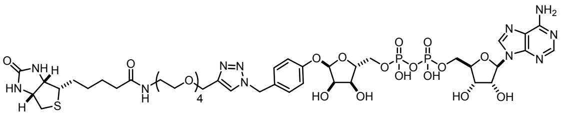

Biotin-Ph-ADPr
6

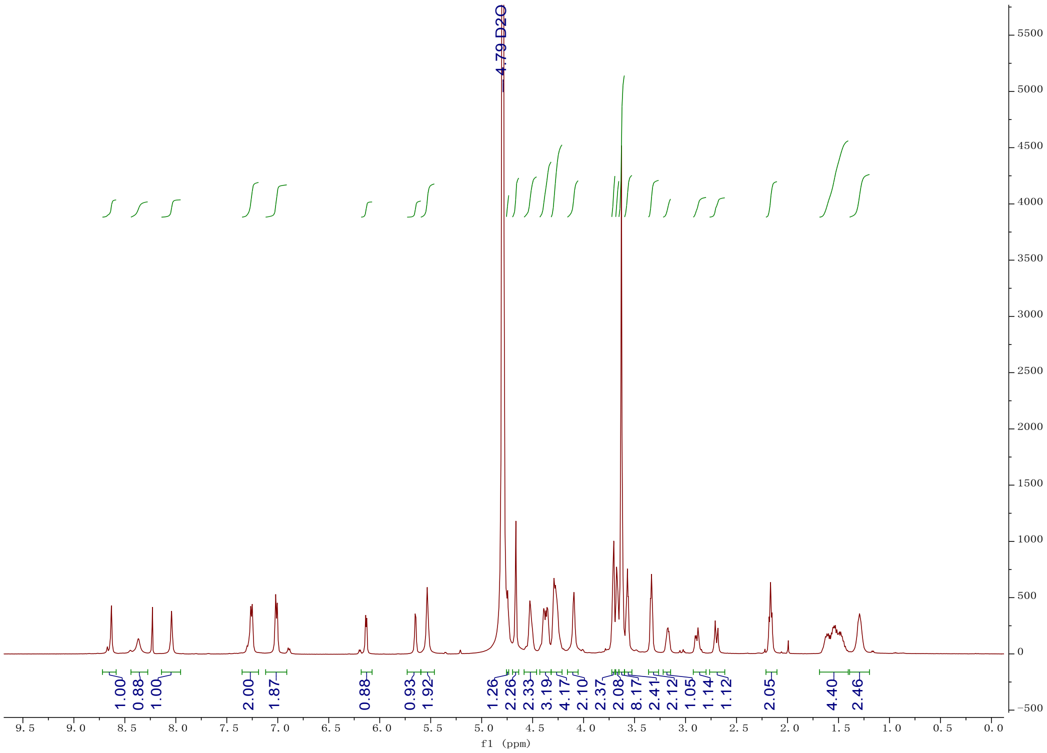

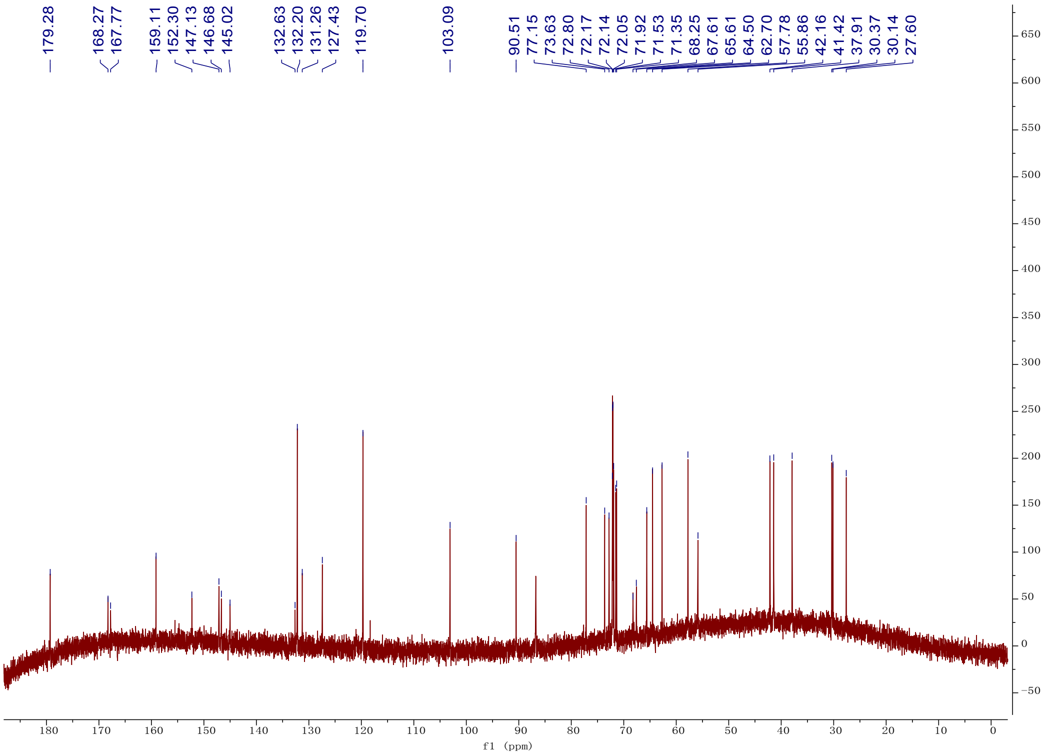

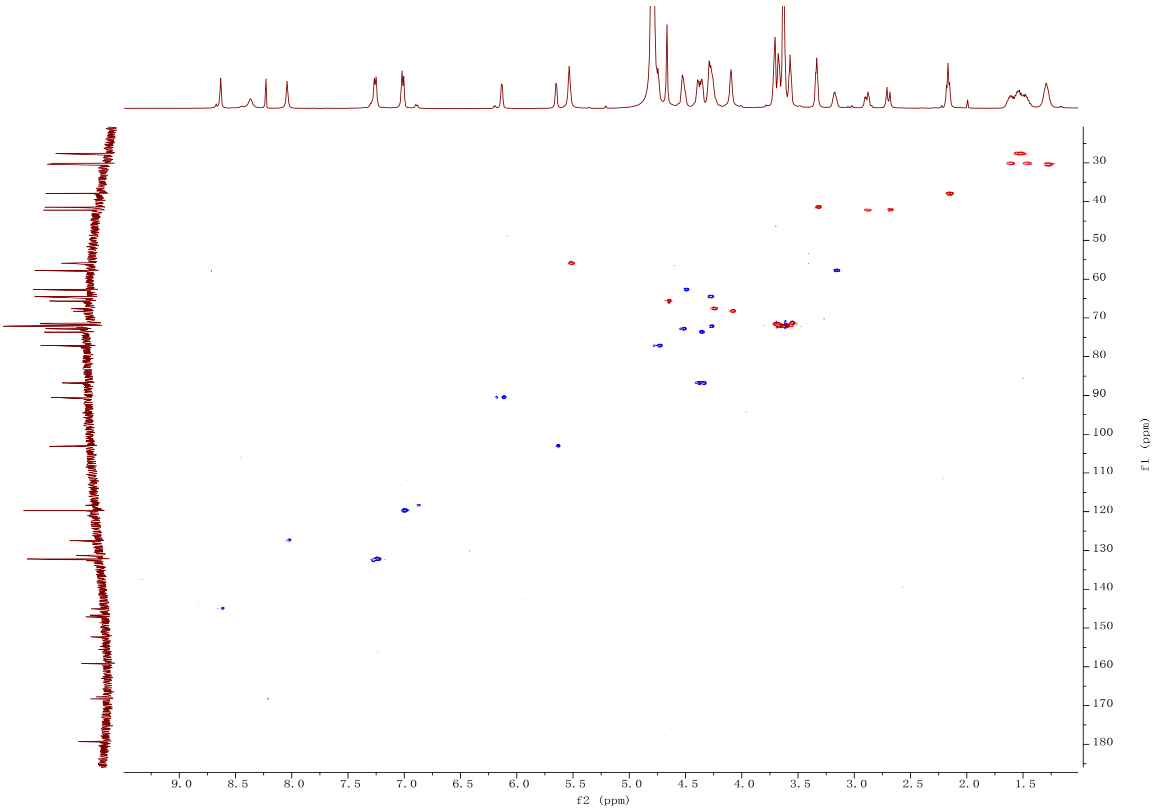

f2 (ppm)
f1 (ppm)

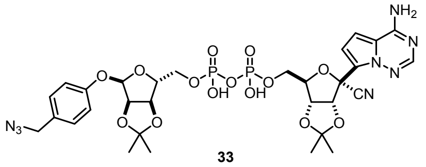
NH2
N3
O
P
OH
CN
N

33

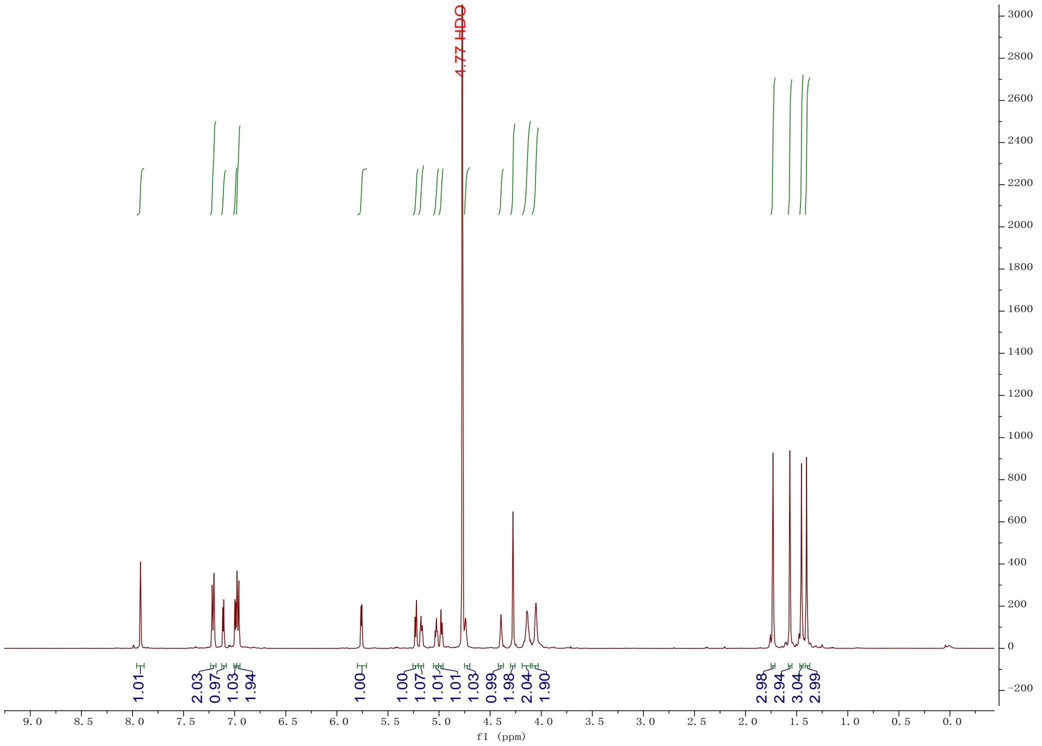
4.77 HDO
1.01
2.03
0.97
1.03
1.94
1.00
1.00
1.07
1.01
1.01
1.03
0.99
1.98
2.04
1.90
2.98
2.94
3.04
2.99
f1 (ppm)

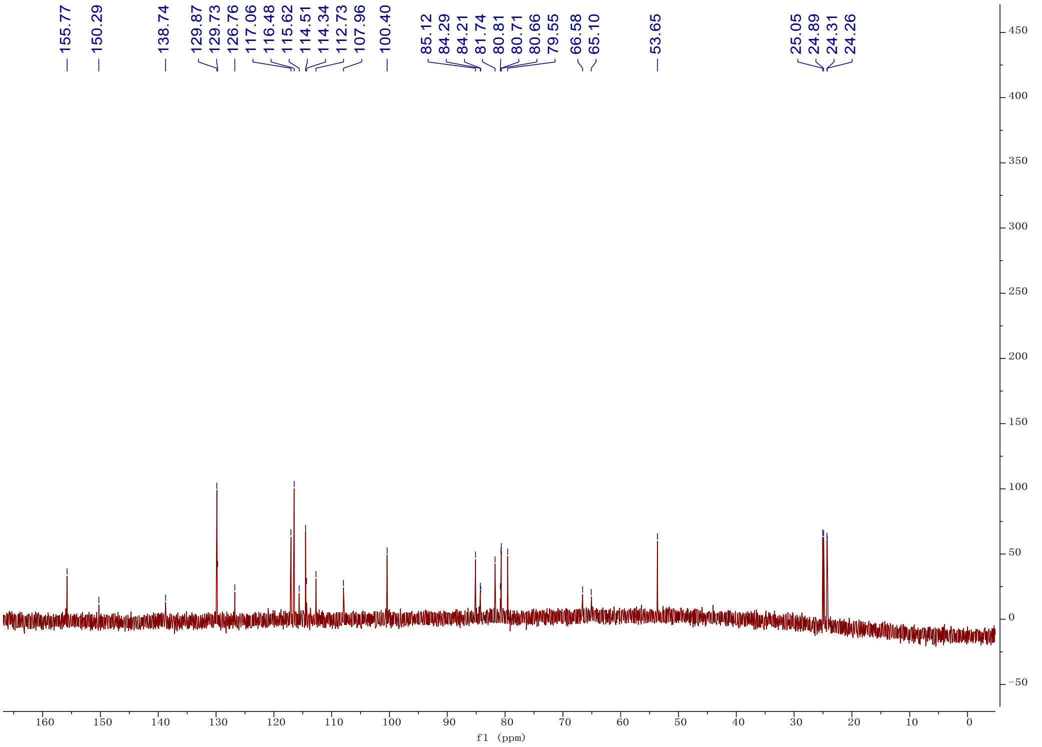
155.77
150.29
138.74
129.87
129.73
126.76
117.06
116.48
115.62
114.51
114.34
112.73
107.96
100.40
85.12
84.29
84.21
81.74
80.81
80.71
80.66
79.55
66.58
65.10
53.65
25.05
24.89
24.31
24.26
f1 (ppm)

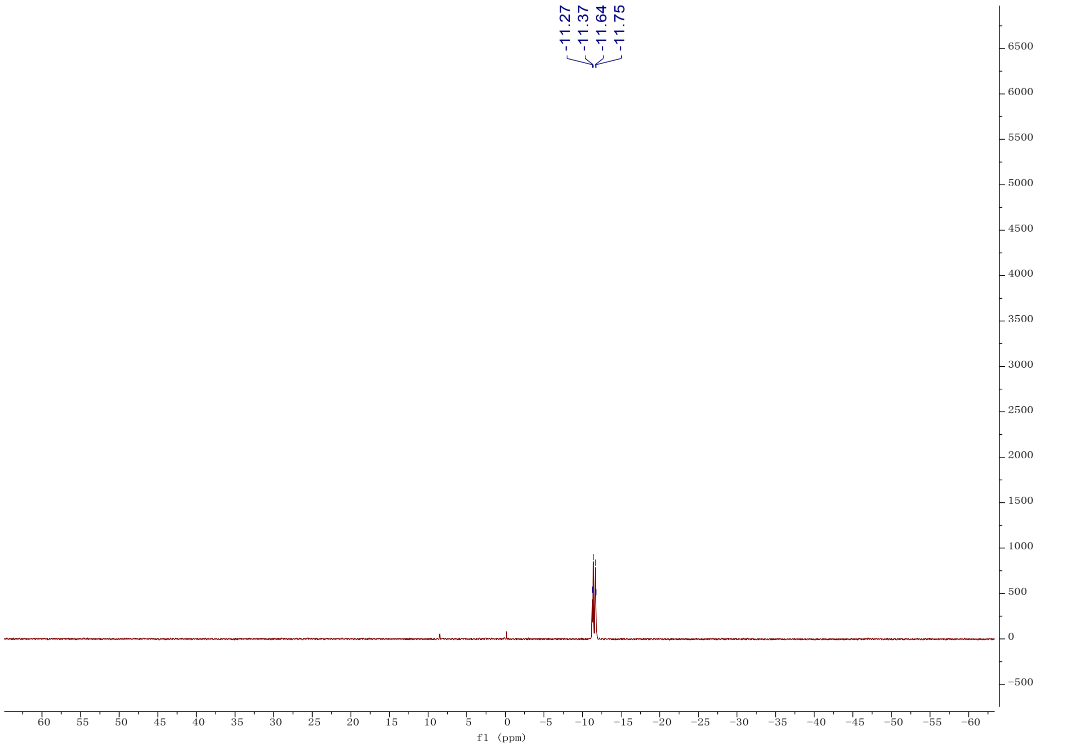

-11.27
-11.37
-11.64
-11.75
f1 (ppm)

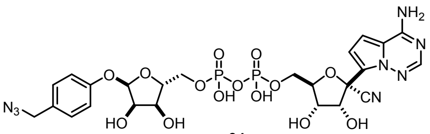
NH2
N3
HO
OH
CN

34

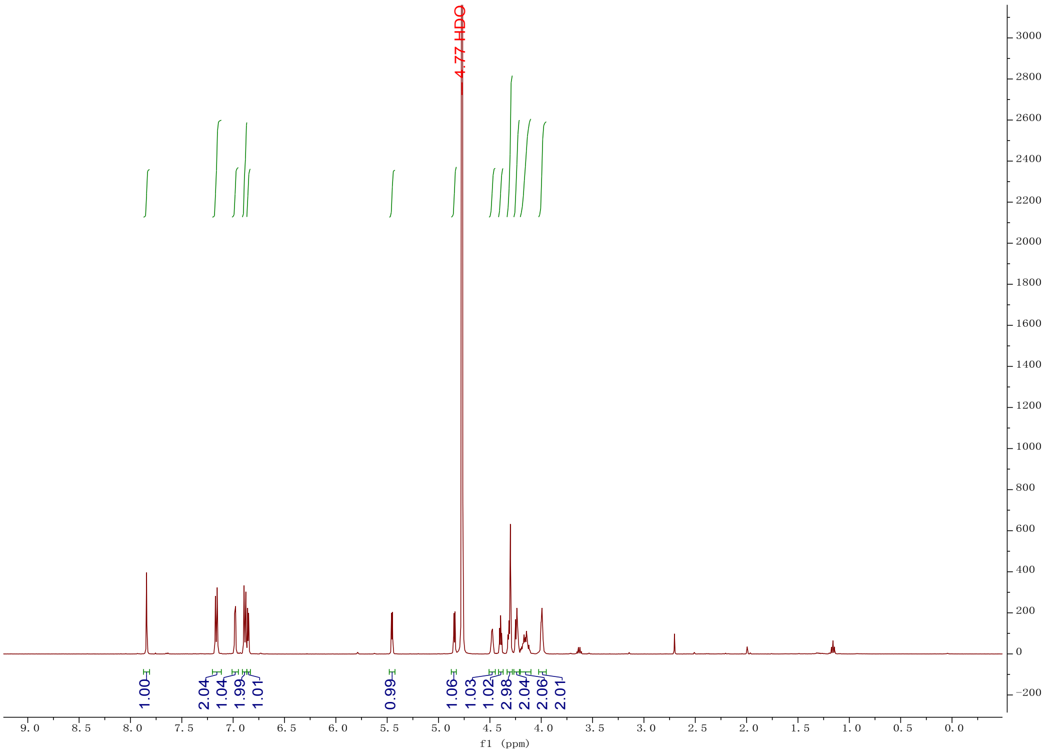
4.77 HDO
1.00
2.04
1.04
1.99
1.01
0.99
1.06
1.03
1.02
2.98
2.04
2.06
2.01
f1 (ppm)

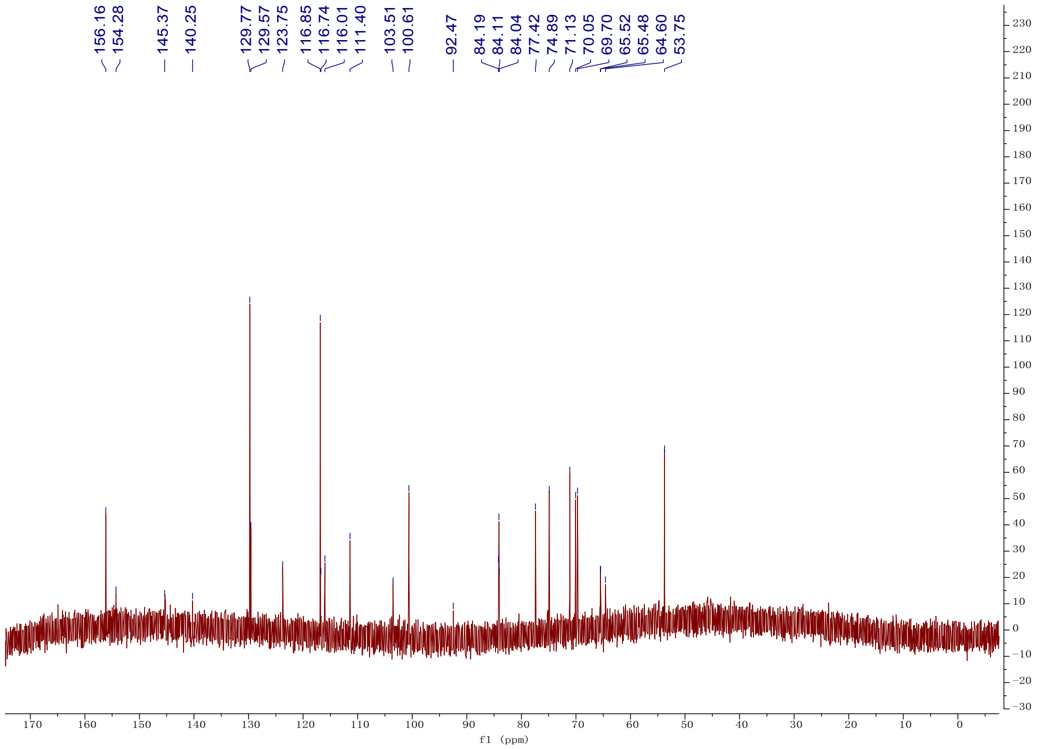
156.16
154.28
145.37
140.25
129.77
129.57
123.75
116.85
116.74
116.01
111.40
103.51
100.61
92.47
84.19
84.11
84.04
77.42
74.89
71.13
70.05
69.70
65.52
65.48
64.60
53.75
f1 (ppm)

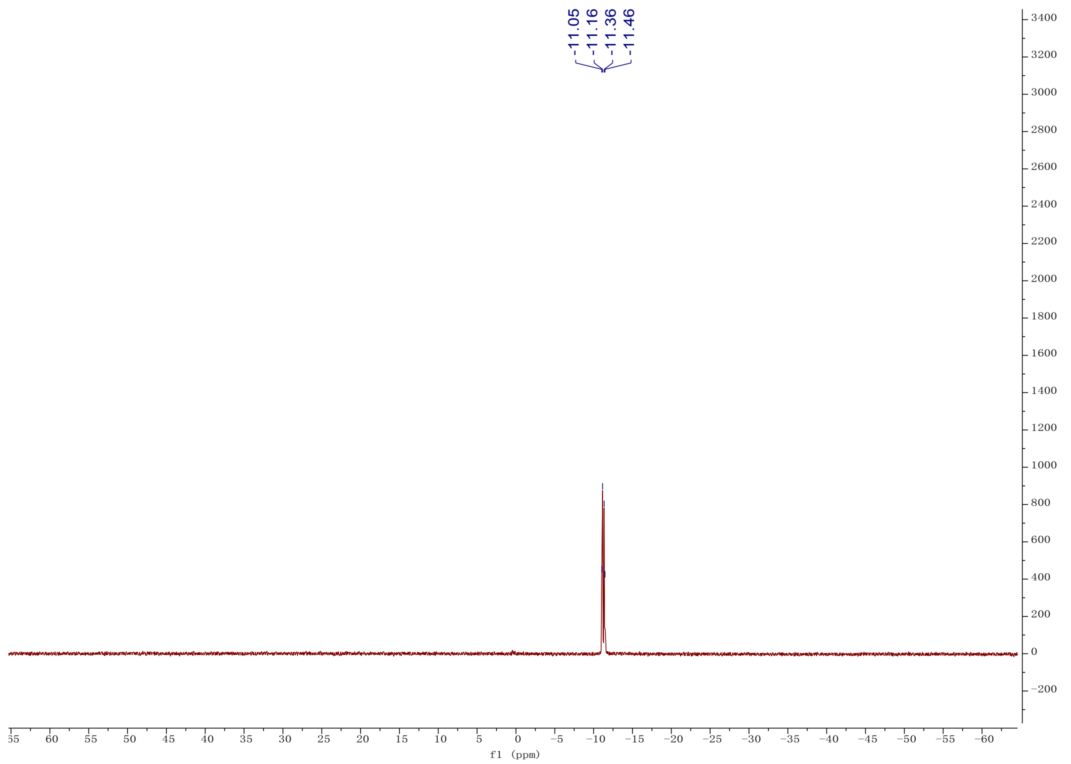
-11.05
-11.16
-11.36
-11.46
f1 (ppm)

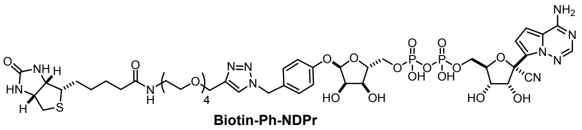

Biotin-Ph-NDPr
8

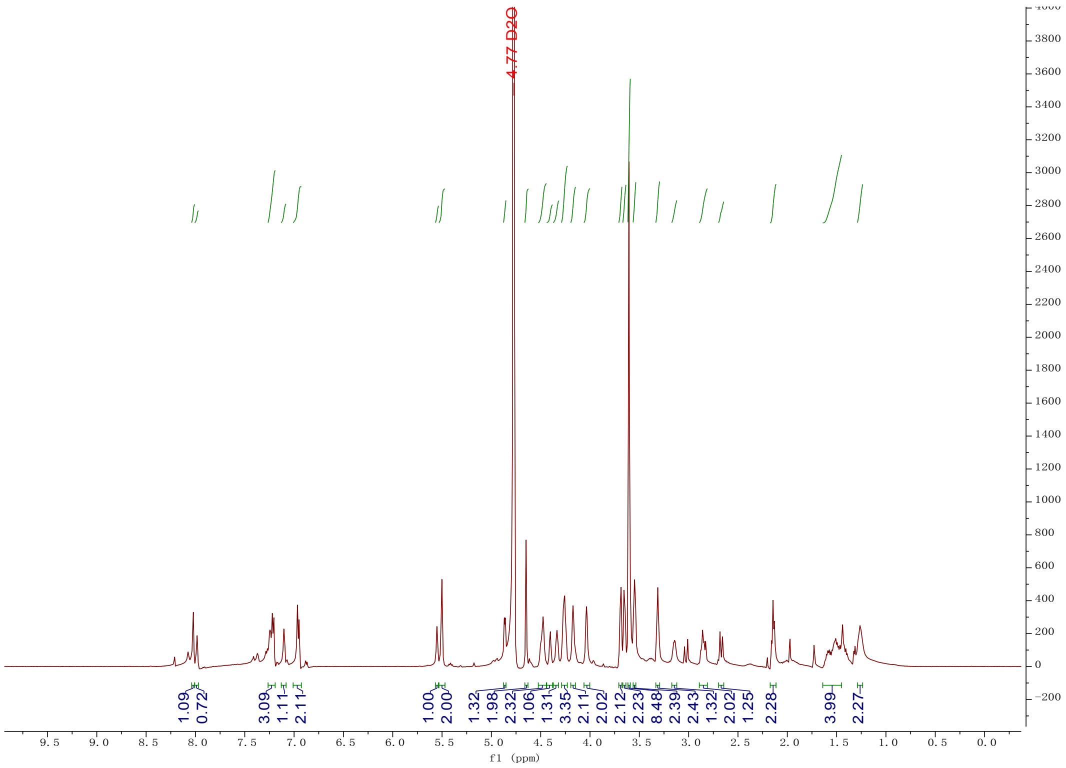

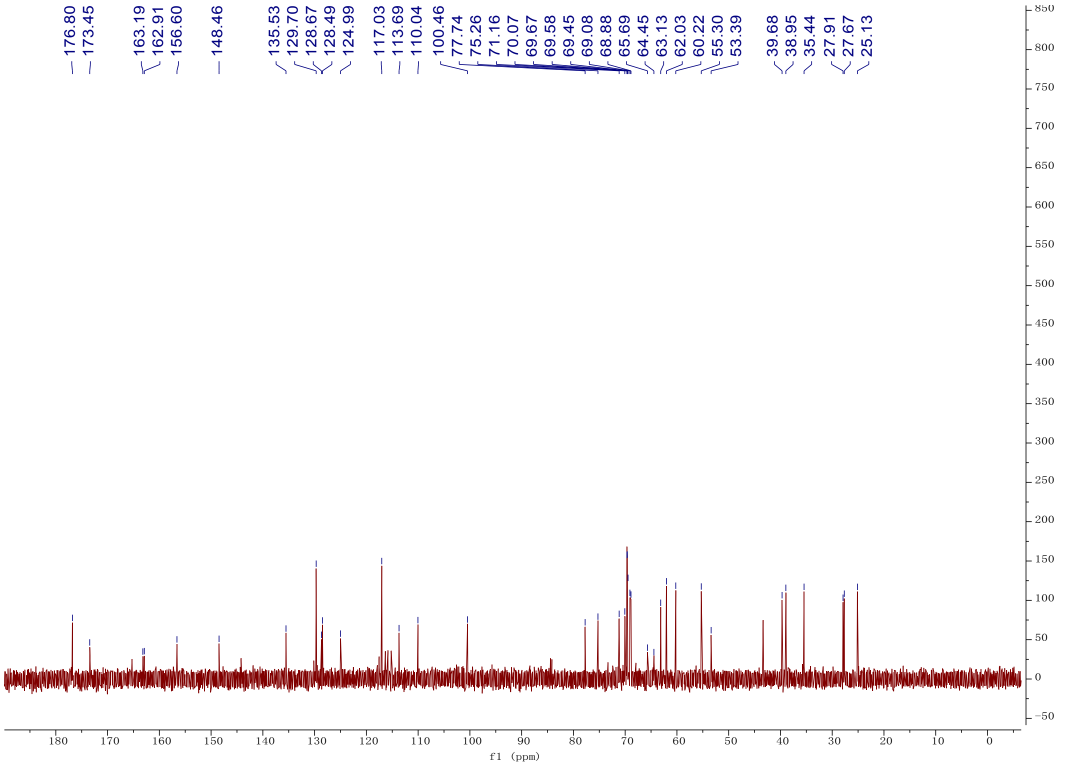

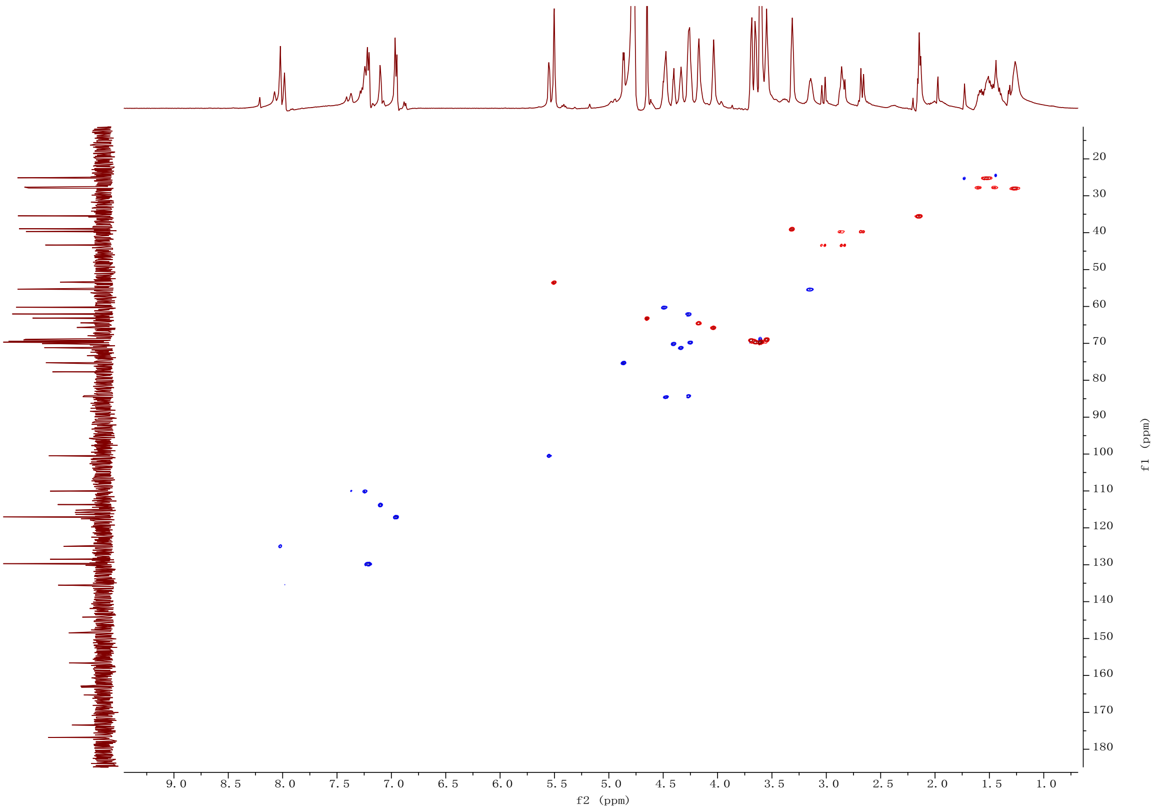

f2 (ppm)
f1 (ppm)

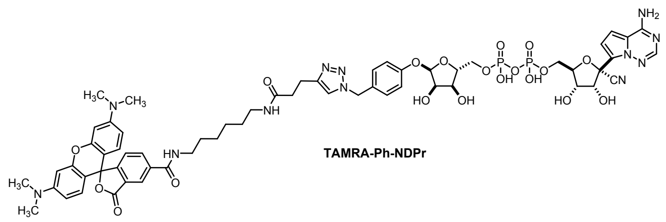

TAMRA-Ph-NDPr

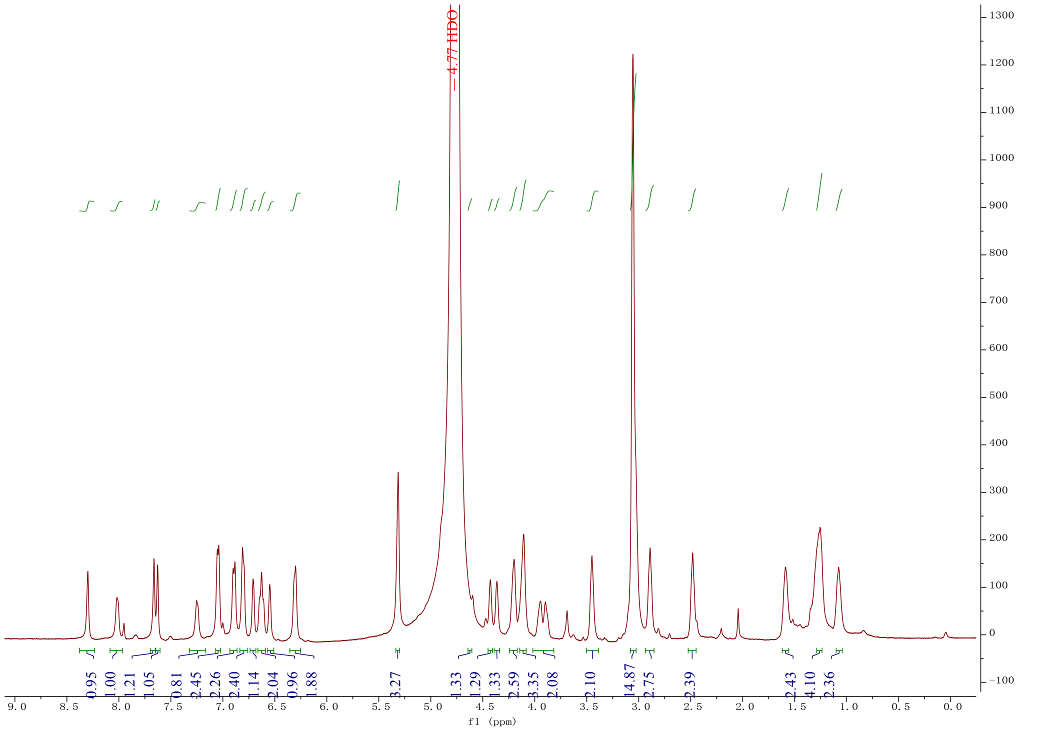

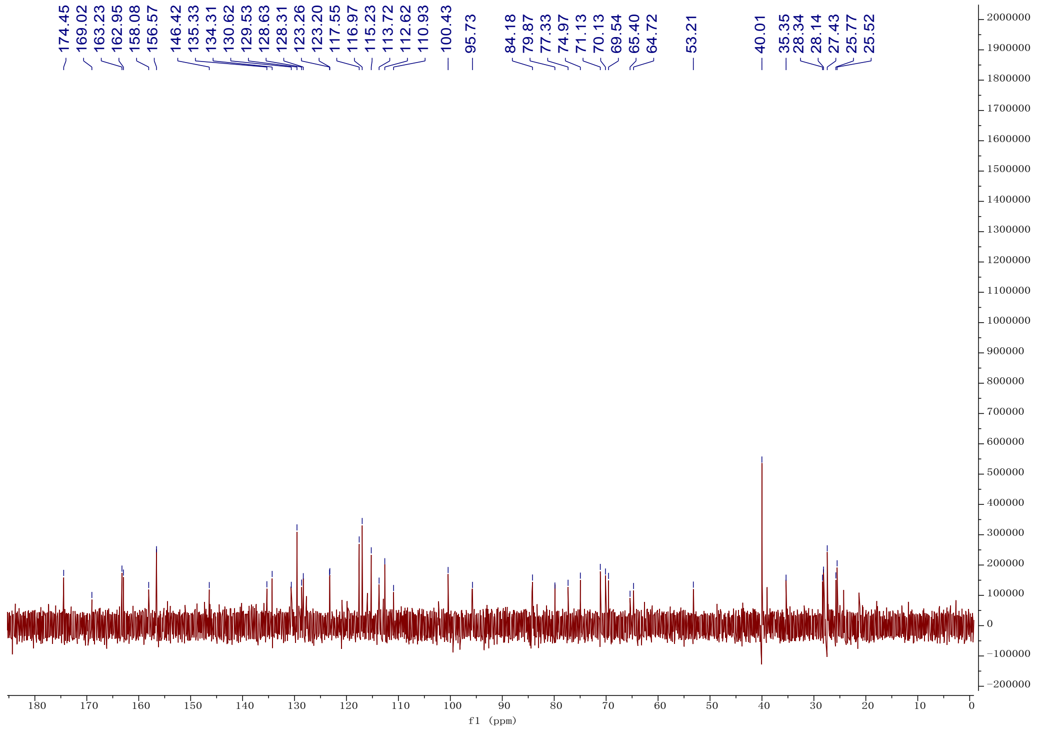

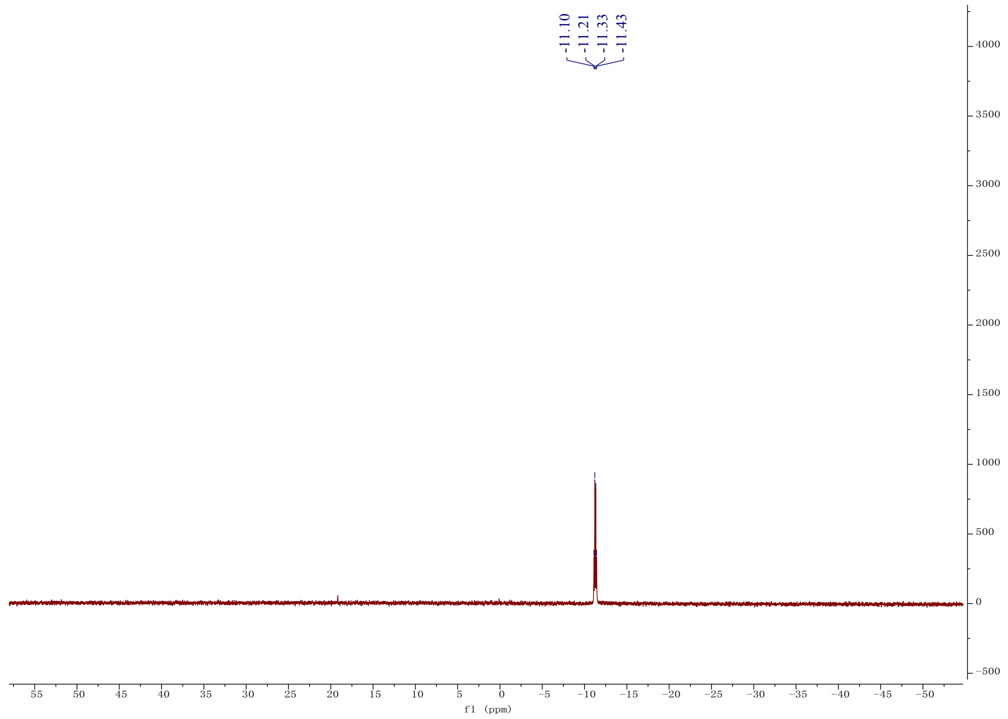

-11.10
-11.21
-11.33
-11.43
f1 (ppm)

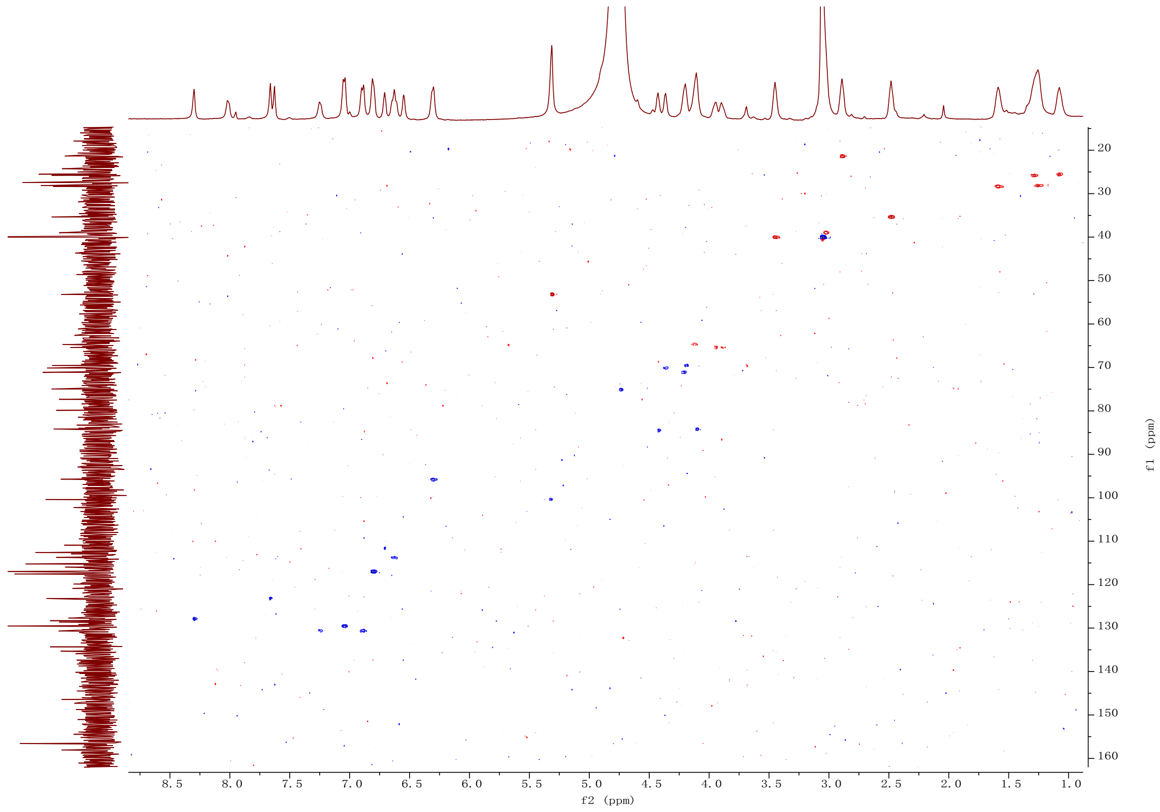

f2 (ppm)
f1 (ppm)
